# Loss of EZH2-like or SU(VAR)3–9-like proteins causes simultaneous perturbations in H3K27 and H3K9 tri-methylation and associated developmental defects in the fungus *Podospora anserina*

**DOI:** 10.1186/s13072-021-00395-7

**Published:** 2021-05-07

**Authors:** F. Carlier, M. Li, L. Maroc, R. Debuchy, C. Souaid, D. Noordermeer, P. Grognet, F. Malagnac

**Affiliations:** 1 Institute for Integrative Biology of the Cell (I2BC), Université Paris-Saclay, CEA, CNRS, 91198 Gif-sur-Yvette, France; 2 Génétique Quantitative et Évolution–Le Moulon, INRA–Université Paris-Saclay–CNRS-AgroParisTech, Batiment 400, UFR Des Sciences, 91405 Orsay CEDEX, France; 3 Present Address: Group Fungal Epigenomics, Department of Mycology, Institut Pasteur, Paris, France; 4 Present Address: Inserm, Theories and Approaches of Genomic Complexity (TAGC), UMR1090, Aix-Marseille University, 13288 Marseille, France

**Keywords:** Histone methylation, Sexual development, ChIP-seq, *Podospora anserina*

## Abstract

**Background:**

Selective gene silencing is key to development. It is generally accepted that H3K27me3-enriched heterochromatin maintains transcriptional repression established during early development and regulates cell fate. Conversely, H3K9me3-enriched heterochromatin prevents differentiation but constitutes protection against transposable elements. We exploited the fungus *Podospora anserina*, a valuable alternative to higher eukaryote models, to question the biological relevance and functional interplay of these two distinct heterochromatin conformations.

**Results:**

We established genome-wide patterns of H3K27me3 and H3K9me3 modifications, and found these marks mutually exclusive within gene-rich regions but not within repeats. We generated the corresponding histone methyltransferase null mutants and showed an interdependence of H3K9me3 and H3K27me3 marks. Indeed, removal of the PaKmt6 EZH2-like enzyme resulted not only in loss of H3K27me3 but also in significant H3K9me3 reduction. Similarly, removal of PaKmt1 SU(VAR)3–9-like enzyme caused loss of H3K9me3 and substantial decrease of H3K27me3. Removal of the H3K9me binding protein PaHP1 provided further support to the notion that each type of heterochromatin requires the presence of the other. We also established that *P. anserina* developmental programs require H3K27me3-mediated silencing, since loss of the PaKmt6 EZH2-like enzyme caused severe defects in most aspects of the life cycle including growth, differentiation processes and sexual reproduction, whereas loss of the PaKmt1 SU(VAR)3–9-like enzyme resulted only in marginal defects, similar to loss of PaHP1.

**Conclusions:**

Our findings support a conserved function of the PRC2 complex in fungal development. However, we uncovered an intriguing evolutionary fluidity in the repressive histone deposition machinery, which challenges canonical definitions of constitutive and facultative heterochromatin.

**Supplementary Information:**

The online version contains supplementary material available at 10.1186/s13072-021-00395-7.

## Background

Histones are subjected to a variety of post-translational covalent modifications [1] that may impact the overall degree of packing of the genome. In most organisms, the opened euchromatin is enriched in tri-methylation of lysine 4 and lysine 36 (H3K4me3 and H3K36me3), two concomitant modifications associated with active transcription [2]. In contrast, histones present in the compacted heterochromatin are enriched in either tri-methylated H3K27 (H3K27me3) or tri-methylated H3K9 (H3K9me3) [3]. The compact architecture of heterochromatin limits the accessibility of the transcription machinery to the embedded DNA, thereby silencing gene expression. Repeat-rich genomic regions enriched in H3K9me3 are referred to as ‘constitutive’ heterochromatin because the subsequent silencing may be constant across development [4]. In contrast, the ‘facultative’ heterochromatin corresponds to the deposition of H3K27me3 on gene-rich regions, whose silencing is transient and dynamic across developmental processes, allowing cell type-specific differentiation and rapid adaptation of gene expression [5].

H3K9me3 is catalyzed by the SET-domain SU(VAR)3–9 enzymes [6–9] and bound by the chromodomain of Heterochromatin Protein 1 (HP1) [10–12]. Subsequent HP1 oligomerization, which results in nucleosome binding [13] and phase separation [14–16], further enhances chromatin compaction and spreads this structure over large genomic compartments. When present, high levels of DNA cytosine methylation are found in H3K9me3-enriched regions. This modification is associated with genome stability by preventing either expression of transposable elements or mitotic recombination. As demonstrated in mouse and drosophila models, when H3K9me3 enzymatic activity is absent or reduced, the embryos die because of diverse developmental defects [17]. Accordingly, H3K9me3-dependent heterochromatin is considered as a barrier to cell fate changes, by preventing some transcription factors to bind DNA [18].

First identified in Drosophila, the conserved Polycomb group (Pc-G) protein complexes were shown to be both writers (Polycomb Repressive Complex 2, PRC2) and readers (Polycomb Repressive Complex 1, PRC1) of H3K27me3 [19–21]. The catalytic subunit of PRC2 is made of the SET-domain protein enhancer of zest homolog 2 (EZH2), which tri-methylates H3K27. The H3K27me3-polycomb modification is instrumental for maintaining transcription repression established during early development and, therefore, has been linked to biological functions related to cell fate, as diverse as X-inactivation [22] and hematopoiesis [23]. Conversely, when PCR2 functions are impaired, development of plants [24], insects [25] and mammals [26, 27] is disrupted, presumably because lineage-specific programs are no longer accurate. Mutations affecting EZH2 lead to cell proliferation in cancer [13], while others are associated with congenital disorders such as the Weaver syndrome [28] or Ataxia-telangiectasia disease [29].

In animals and flowering plants, H3K9me3 and H3K27me3 marks are almost always mutually exclusive [30], but the molecular basis for this exclusion is unknown. To further investigate the genomic distribution and functional interplay of these two marks, filamentous fungi of the Pezizomycotina clade are a valuable alternative to higher eukaryotic models. Unlike ascomycete yeasts [8], most of them display activity of both H3K9 and H3K27 histone methyltransferase enzymes [31]. The role of the H3K27 histone methyltransferase was investigated in the model fungus *Neurospora crassa* [32–40], the human pathogen *Cryptococcus neoformans* [41], the plant endophyte *Epichloë festucae* [42] and the plant pathogens *Fusarium graminearum* [43], *Fusarium fujikuroi* [44], *Zymoseptoria tritici* [45] and *Magnaporthe oryzae* [46]. As for metazoans and plants, H3K27me3 is essential for proper development of *F. graminearum* [43], *F. fujikuroi* [44] and *M. oryzae* [46] (including growth, sporulation, fertility, pathogenicity, and expression of mycotoxins, pigments, and other secondary metabolites). Paradoxically, deletion of the PRC2 enzymatic subunit of *N. crassa*, *C. neoformans* or *Z. tritici* does not result in obvious development defects. However, H3K9me3 methylation is required for *N. crassa* normal growth and full fertility [47, 48] and in the wheat pathogen *Z. tritici*, lack of H3K9me3 induces genomic rearrangements. H3K9me3 and H3K27me3 modifications do not overlap in these fungal genomes, a pattern also found in animals and plants. Interestingly, depletion of H3K9me3 in either *N. crassa* or *Z. tritici* leads to a massive redistribution of H3K27me3 in genomic compartments that were previously embedded in constitutive H3K9me3 heterochromatin [35, 45]. Altogether, these reports present a contrasted picture, suggesting that connections between heterochromatin features and regulation of gene expression need to be further investigated in fungi.

In this study, we used the model system *P. anserina* to establish the genome-wide distribution of H3K4me3, H3K9me3 and H3K27me3. Most of the H3K9me3 is found in repeat-rich regions also enriched in H3K27me3, while both the H3K9me3 and H3K27me3 are co-exclusive with the H3K4me3 marks. We demonstrated that absence of PaKmt1, the H3K9me3 methyltransferase, resulted in loss of H3K9me3 and in significant reduction of H3K27me3, but had a limited impact on *P. anserina* growth, differentiation and sexual reproduction. Likewise, removal of PaHP1 diminished H3K9me3 and H3K27me3 modifications, with impact on *P. anserina* physiology similar to that of the PaKmt1 mutation. Conversely, we observed that absence of PaKmt6, the H3K27me3 methyltransferase, resulted in drastic depletion of both H3K27me3 and H3K9me3 and caused severe defects in most aspects of the life cycle including growth, differentiation processes and sexual reproduction. These findings point towards an interdependence of the two types of heterochromatin within the repeated regions of the genome as well as a conserved function of Polycomb silencing in *P. anserina* development.

## Results

### Chromatin-modifying enzymes are present in the *P. anserina* genome and expressed throughout its life cycle

**Fig. 1:**
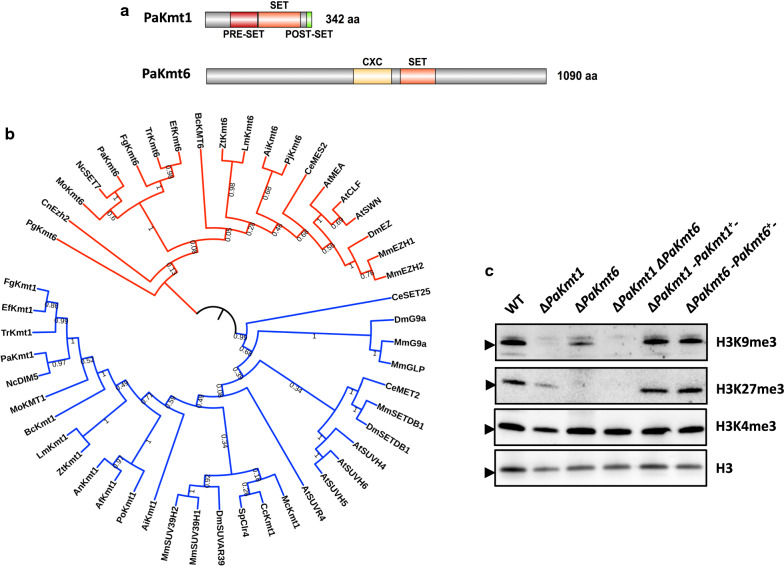
Structure and functions of the histone methyltransferases PaKmt1 and PaKmt6. **a** Domain structure of histone methyltransferases PaKmt1 and PaKmt6. Sizes in amino acid (aa) are given (right). PRE-SET (red, IPR007728), SET (orange, IPR001214) and POST-SET (green, IPR003616) conserved domains are required for H3K9 methyltransferase activity of KMT1 homolog proteins. CXC (yellow, IPR026489) is a cysteine-rich conserved domain located in the H3K27 methyltransferase catalytic domain of KMT6 homologs. **b** Phylogenetic analysis of KMT1 and KMT6 histone methyltransferase homologs. This tree regroups H3K9 (blue) and H3K27 (red) methyltransferase proteins from fungi, plants and metazoans. They cluster in two different clades of histone methyltransferases. Their evolutionary history is canonical since the topology of the two branches of the tree is consistent with the species tree. Bootstraps are given. Filamentous fungi: *Podospora anserina* (Pa), *Neurospora crassa* (Nc), *Fusarium graminearum* (Fg), *Trichoderma reesei* (Tr), *Epichloë festucae* (Ef) *Botrytis cinerea* (Bc), *Magnaporthe oryzae* (Mo), *Zymoseptoria tritici* (Zt), *Leptosphaeria maculans* (Lm), *Aspergillus nidulans* (An), *Aspergillus fumigatus* (Af), *Penicillium oxalicum* (Po), *Ascobolus immersus* (Ai), *Puccinia graminis* (Pg), *Pneumocystis jirovecii* (Pj), *Conidiobolus coronatus* (Cc), *Mucor circinelloides* (Mc); yeasts: *Schizosaccharomyces pombe* (Sp) and *Cryptococcus neoformans* (Cn); the worm *Caenorhabditis elegans* (Ce), the fruit-fly *Drosophila melanogaster* (Dm), the mouse *Mus musculus* (Mm) and the model plant *Arabidopsis thaliana* (At). Accession numbers for proteins used in alignments are listed in Additional file 24: Table S6. **c** Detection of histone post-translational modifications Western-immunoblot analysis of H3K9me3, H3K27me3 and H3K4me3 modifications in wild-type (WT); single mutants Δ*PaKmt1*, Δ*PaKmt6*; double mutant Δ*PaKmt1*Δ*PaKmt6*; complemented strains Δ*PaKmt1-PaKmt1*^+^ (*PaKmt1*, *PaKmt1-GFP-HA*), Δ*PaKmt6-PaKmt6*^+^ (*PaKmt6*, *PaKmt6-HA*). Nuclear protein samples were extracted from protoplasts. A total of 35 µg of nuclear protein was loaded in each lane. Antibodies were directed against native H3 histone (H3), H3K9me3, H3K27me3 and H3K4me3 modifications. We detected some marginal cross-reactivity between the anti-H3K9me3 antibody and the H3K27me3 modification (evaluated at 3%, see Additional file 7: Fig. S7). Expected size of histone 3 is 17 kDa (arrows indicated 15 kDa)

The *P. anserina* genome contains a single gene (*Pa_6_990*) encoding a putative H3K9me3 methyltransferase, named PaKmt1 (342 aa) and a single gene (*Pa_1_6940*) encoding a putative H3K27me3 methyltransferase, named PaKmt6 (1090 aa). Both of these highly conserved proteins show a canonical SET domain typical of *bona fide* histone methyltransferases (Fig. 1a and Additional file 1: Fig. S1A–C). PaKmt1 displays pre- and post-SET domains (PS50867 and PS50868) (Additional file 1: Fig. S1D). PaKmt6 contains a CXC domain (PS51633), a cysteine-rich motif frequently found in polycomb group (Pc-G) proteins, but no chromodomain. PaKmt1 and PaKmt6 cluster in two different clades of histone methyltransferase (Fig. 1b). Their evolutionary history is canonical since the topology of the two branches of the tree is consistent with the species tree.

RNA-seq experiments detected *PaKmt6* transcripts of predicted structure in both vegetative mycelium and fruiting bodies [49]. However, while annotation of the *PaKmt1* gene predicted that its ORF was made of two exons, RNA-seq experiments showed ambiguous profiles for *PaKmt1* transcripts [49]. Dedicated reverse-transcription polymerase chain reaction (RT-PCR) experiments revealed two distinct transcripts (Additional file 2: Fig. S2). From 1-day-old mycelium cDNA, we amplified a single 1108-bp fragment corresponding to the *PaKmt1* spliced transcript. However, from either 4-day-old mycelium RNA extracts or fruiting body RNA extracts (2-day and 4-day post-fertilization fruiting bodies), we amplified an additional 1170-bp fragment, which corresponds to a *PaKmt1* unspliced transcript. The translation of the 1170-bp fragment results in a truncated protein of 76 amino acids lacking the conserved functional motifs (pre-SET and SET domains). This observation suggests that, in addition to the functional full length PaKmt1 H3K9 methyltransferase, *P. anserina* may produce a truncated version of this protein at specific developmental stages of its life cycle.

### Genome-wide distribution of H3K4me3, H3K9me3 and H3K27me3 histone modifications in the *P. anserina* genome

**Fig. 2:**
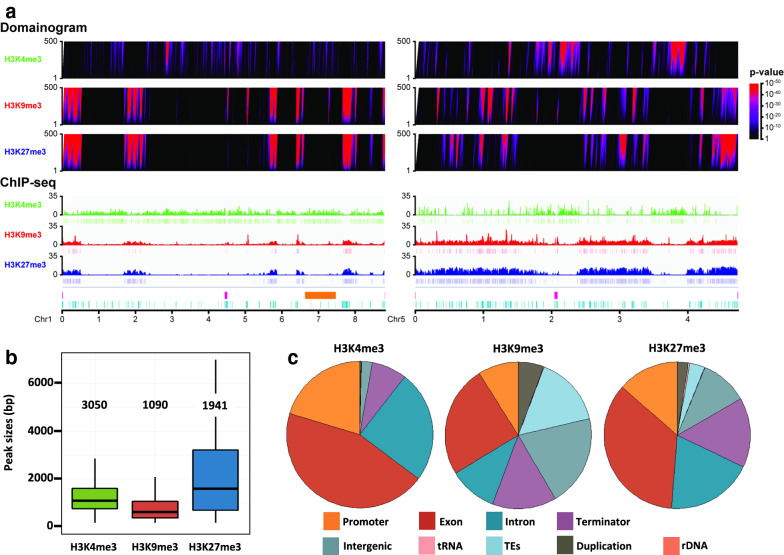
Distribution of histone marks in wild-type *P. anserina*. **a** Wild-type distribution of H3K4me3, H3K9me3 and H3K27me3 on chromosomes one and five. Domainograms (top) show significance of enrichment of H3K4me3, H3K9me3, H3K27me3 marks in windows of varying size. Color-coding of *p*-value is indicated (right). ChIP-seq patterns (bottom) display histone modification coverage and MACS2 detected peaks. Both patterns are represented with the same scale on chromosomes one and five of *P. anserina.* Both centromeres are depicted (pink). The “Mat region” (orange) corresponds to the 800 kbp region devoid of recombination and containing the mating-type locus [93]. Repeated sequences are depicted in light blue. **b** Peak size distribution in the wild-type *P. anserina*. Box plots showing sizes (in base pairs) of the MACS2-predicted peak of H3K4me3 (green), H3K9me3 (red) and H3K27me3 (blue). The box is delimited, bottom to top, by the first quartile, the median and the third quartile. Numbers above each box represent the number of peaks detected. Outliers are not shown. **c** Repartition of histone marks on annotated genome features in wild-type *P. anserina*. The pie charts represent the proportion of peaks that overlap the genomic features indicated in the legend panel

Using western-blotting, we first showed that H3K4me3, H3K9me3 and H3K27me3 marks were present in the *P. anserina* genome (Fig. 1c, WT). Then, to further characterize the genomic patterns of these three histone modifications, we performed ChIP-seq experiments on chromatin samples collected from vegetative mycelia grown 60 h at 27 °C. For mapping genome-wide patterns of histone modifications, we used two distinct methodologies (i) domainogram analysis [50] based on enrichment within multiscale windows and (ii) peak calling analysis using the MACS2 algorithm (Fig. 2a and Additional file 19: Table S1). Clustering analysis of histone marks in the wild-type background established that the biological replicates clustered together with high correlation coefficients (Additional file 3: Fig. S3A).

**Fig. 3:**
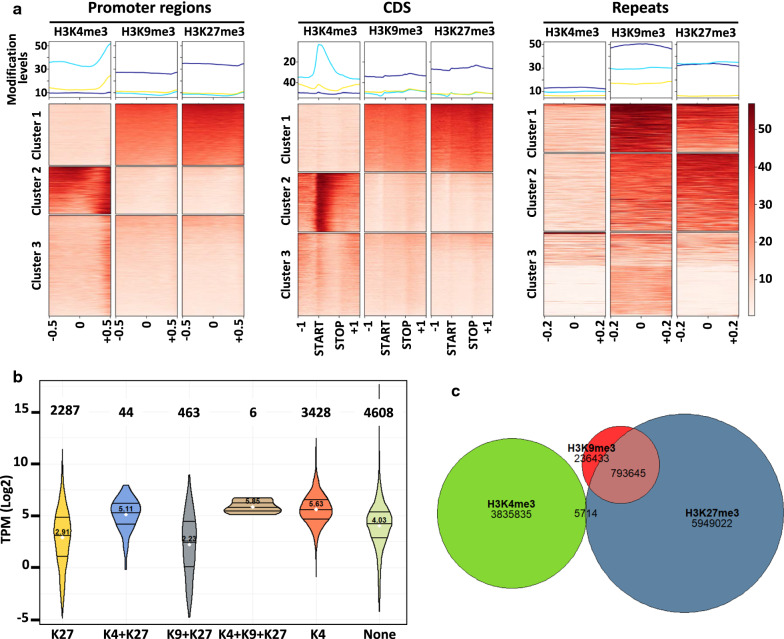
Presence of histone marks in promoters, genes and TEs in the wild-type strain. **a** Metaplots. Top panel: plots of averaged ChIP-seq signal. Bottom panel: heatmaps divided in K-means built clusters representing the association versus non-association of the indicated histone modifications with the specific genomic regions. Promoter regions were defined as 1 kbp 5′ of the start codon ± 0.5 kbp of surrounding sequence (*N* = 10,835 Additional file 20: Table S2); coding sequences or CDS were aligned by their two ends (indicated by START and STOP) ± 1 kbp of surrounding sequence (*N* = 10,839 Additional file 20: Table S2); repeats were defined as TE body and duplications and the rDNA array ± 0.2 kbp surrounding each region (*N* = 1680 Additional file 21: Table S3). Histone modification levels in the heatmaps were calculated for non-overlapping 10 bp windows within the specific genomic regions and sorted by average value of each row. **b** Violin plots of gene expression according to the histone mark. Gene expression was inferred from the Transcripts Per Kilobase Million (TPM) values calculated in [49]. Log2 of the TPM values was plotted according to the marks detected for the CDS. The three lines of the violin plot represent the first quartile, the median and the third quartile. The white dot and the number above represent the mean. The numbers on top represent the number of genes in each category. K4: H3K4me3, K9: H3K9me3, K27: H3K27me3. **C.** Euler diagram showing overlap between the different histone marks. H3K4me3, H3K9me3 and H3K27me3 are represented by the green, red and blue areas, respectively. The numbers indicate the number of base pairs covered by each mark, according to MACS2-predicted peaks. Overlap between areas shows the number of base pairs covered by two marks

The H3K4me3 modification (Fig. 2a, Additional file 3: Fig. S3B–D, Additional file 4: Fig. S4 and Additional file 19: Table S1, Additional file 20: Table S2 and Additional file 21: Table S3) covered nearly 11% of the *P. anserina* genome, with 3050 detected peaks. This mark appeared to be present in blocks of approximately 1.3 kb (Fig. 2b) in gene-rich regions, mostly located in coding sequences (CDS, Figs. 2c and 3a and Additional file 20: Table S2) and in 5′ UTR (Figs. 2c and 3a). As in other eukaryotes, H3K4me3 showed a positive correlation with active transcriptional activity (Fig. 3b). On rare instances, when H3K4me3 peaks appeared to be localized in repeats (Figs. 2c and 3a, Additional file 5: Fig. S5A, B and Additional file 21: Table S3), it mainly corresponded to transposable element (TE) relics or transposase genes inserted into coding sequences (49 out of the 63 H3K4me3 peaks found in repeats).

Conversely, H3K9me3 (Fig. 2a, Additional file 3: Fig. S3B–D and Additional file 19: Table S1, Additional file 20: Table S2 and Additional file 21: Table S3) was the least abundant mark found in *P. anserina* genome (1090 detected peaks, 2.94% of the genome), forming approximately 0.9-kb-long blocks (Fig. 2b). This mark is mostly found at repeat-rich regions, i.e., TE (728 peaks, Additional file 21: Table S3), centromeric regions and telomeric regions (Figs. 2c and 3a), so that the abundance of H3K9me3 modification in the *P. anserina* genome reflected its repeat content, omitting the rDNA cluster [51]. In accordance with its genomic distribution, the H3K9me3 modification showed a negative correlation with active transcriptional activity (Fig. 3b). Annotated TE families (i.e., Copia, Gypsy TEs, Tc1 mariner TEs and soloLTR relics) were marked with H3K9me3, and transcriptionally silenced, with the exception of MITEs (Additional file 5: Fig. S5A–C, Additional file 6: Fig. S6 and Additional file 21: Table S3). In the *P. anserina* genome, nearly all copies of TEs differ by polymorphisms generated by the repeat-induced point mutation (RIP) mechanism [51]. Originally described in *N. crassa* [52], RIP is a mutagenic process that occurs during the sexual dikaryotic stage of many filamentous fungi (for review see [53]). In addition of C–G to T–A transition accumulations, the RIPed repeats are embedded in H3K9me3-rich heterochromatin, which leads to transcriptional gene silencing. MITEs are an exception to this pattern because their short length (< 500 pb, Additional file 21: Table S3) makes them less prone to RIP.

The second most common mark found in the *P. anserina* genome in terms of peak numbers (1941 detected peaks, Additional file 19: Table S1), H3K27me3 nonetheless covered 18% of the genome (Fig. 2a, Additional file 3: Fig. S3B–D, Additional file 4: Fig. S4 and Additional file 21: Table S3), showing large blocks approximately 3.5 kb long (Fig. 2b). This mark was found enriched at sub-telomeric regions (Figs. 2c and 3a, Additional file 3: Fig. S3C, D and Additional file 4: Fig. S4), but also on about one third of the annotated coding sequences (2992 detected peaks, Additional file 20: Table S2). Most of these H3K27me3-marked coding sequences were likely not expressed during the vegetative growth phase (Fig. 3b). Importantly, H3K27me3 modification was also found on all TE families, including members of MITEs, soloLTR relics and unclassified TEs (Additional file 5: Fig. S5A, B, Additional file 6: Fig. S6 and Additional file 21: Table S3).

Zooming in on the *P. anserina* genomic patterns, it appeared that distribution of H3K4me3 was found to be mutually exclusive with the distributions of both H3K9me3 and H3K27me3 (Fig. 3c, Additional file 3: Fig. S3C, D, and Additional file 4: Fig. S4). This observation was further confirmed by both the domainograms, where domains with most significant enrichment of the H3K9me3 and H3K27me3 marks, but not the H3K4me3 mark, showed a large degree of overlap (Fig. 2a and Additional file 20: Table S2) and the Spearman correlation test of individual bins, where the H3K9me3 and H3K27me3 IPs were grouped in the same cluster and were negatively correlated with the H3K4me3 cluster (Additional file 3: Fig. S3A). On the same note, we noticed that chromosome five is significantly enriched in H3K9me3 and depleted in H3K4me3 (chi^2^ test, *p*-value < 2.2e−16), which is in line with its high TE and repeat content. However, we did find a few genes that harbored both H3K4me3 and H3H27me3 marks in these experimental conditions (Additional file 20: Table S2). Remarkably, this set was significantly enriched (*p*-value < 0.001) in genes encoding proteins involved in non-allelic heterokaryon incompatibility (HI), transcription and chromatin remodeling (Additional file 20: Table S2). More importantly, patterns of H3K27me3 and H3K9me3 modifications, determined either as peaks or by significant genomic coverage, were often found overlapping in gene-poor compartments scattered across the genome (Fig. 3c, Additional file 3: Fig. S3C, D, Additional file 4: Fig. S4 and Additional file 6: Fig. S6), especially on repeats, which included most of the TE families (Fig. 3a: cluster_1, and Additional file 21: Table S3). Indeed, we identified 954 H3K27me3 and H3K9me3 common peaks, representing 90% of the H3K9me3 peaks (Additional file 19: Table S1 and Additional file 21: Table S3). This observation was consistent with the Spearman clustering analysis showing that H3K9me3 and H3K27me3 IP samples belong to the same cluster (Additional file 3: Fig. S3A).

### PaKmt1 and PaKmt6 are histone methyltransferases and deletion of their genes impacts both H3K9me3 and H3K27me3 distribution

To determine the histone methyltransferase(s) required for the deposition of the heterochromatin marks, we constructed Δ*PaKmt1* and the Δ*PaKmt6* deletion mutants (Additional file 8: Fig. S8). Double Δ*PaKmt1*Δ*PaKmt6* mutant strains were obtained by crossing the corresponding single mutants. All three mutants were viable, demonstrating that neither *PaKmt1* nor *PaKmt6* are essential genes. Western-blotting showed that H3K9me3 was almost absent in proteins extracted from the Δ*PaKmt1* mutant (Fig. 1c), whereas the H3K27me3 was absent from the Δ*PaKmt6* sample. The faint remaining signal seen in the Δ*PaKmt1* sample could be due to the cross-reactivity between the anti-H3K9me3 antibody and the H3K27me3 modification, which was evaluated at 3% of the specific anti-H3K9me3/H3K9me3 immunogenic reaction (Additional file 7: Fig. S7). Importantly, neither mark was present in the double Δ*PaKmt1*Δ*PaKmt6* mutant, showing that no additional H3K9 or H3K27 methylation exists in *P. anserina*. As expected, H3K4me3 was detected in all samples. We concluded that PaKmt1 is required for H3K9me3, while PaKmt6 is required for H3K27me3.

**Fig. 4:**
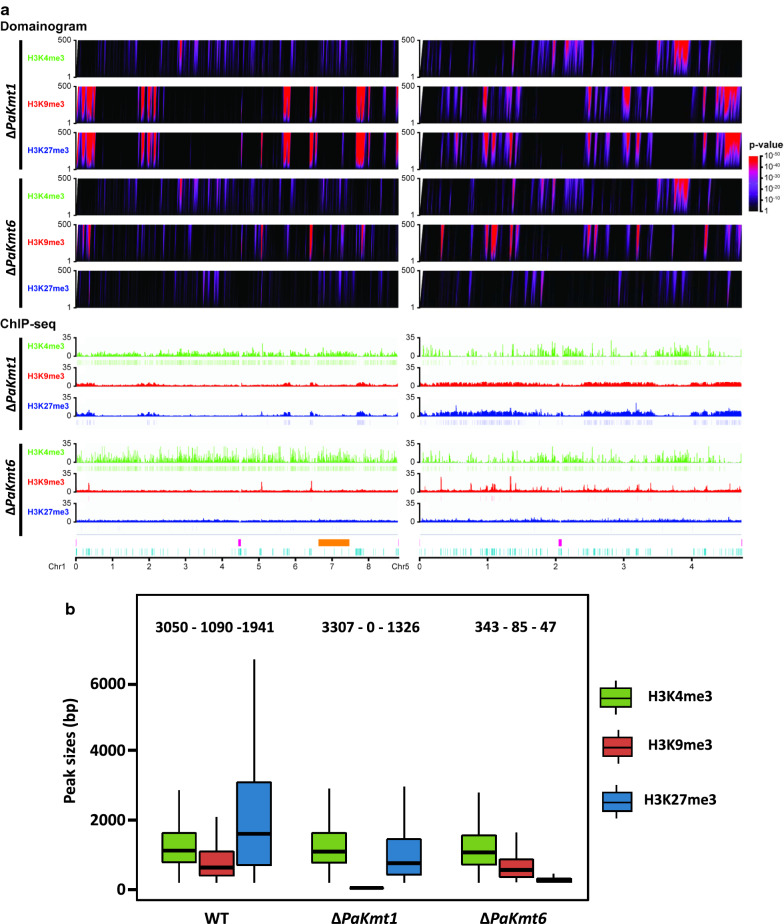
Distribution of histone marks in Δ*PaKmt6* and Δ*PaKmt1* strains. **a** Visualization of H3K4me3, H3K9me3 and H3K27me3 localization across the genome in wild-type and mutant backgrounds. Domainograms (top) show significance of enrichment of H3K4me3, H3K9me3 and H3K27me3 marks in windows of varying size, according to the indicated *p*-value (right). ChIP-seq patterns (bottom) display histone modification coverage and MACS2 detected peaks. Both patterns are represented with the same scale on chromosomes one and five of *P. anserina.* Both centromeres are depicted (pink). The “Mat region” (orange) corresponds to the 800 kbp region devoid of recombination and containing the mating-type locus [93]. Repeated sequences are depicted in light blue. **b** Peak size distribution in the Δ*PaKmt1* and Δ*PaKmt6* mutant strains. Box plots showing sizes in base pairs of the MACS2-predicted peaks of H3K4me3, H3K9me3 and H3K27me3, shown in green, red and blue, respectively. The box is delimited, bottom to top, by the first quartile, the median and the third quartile. Numbers on top of each box represent the number of peaks detected. Outliers are not shown

ChIP-seq experiments performed with either Δ*PaKmt1* mutants or Δ*PaKmt6* mutants showed that the H3K4me3 patterns remained unchanged in all the mutant genetic contexts, suggesting that loss of H3K27me3 or H3K9me3 has no impact on the H3K4 methyltransferase activity (Fig. 4a, b, Additional file 3: Fig. S3B–D, Additional file 4: Fig. S4 and Additional file 19: Table S1 and Additional file 20: Table S2).

According to domainogram analysis, ChIP-seq experiments performed in the Δ*PaKmt1* background revealed a nearly comparable span of H3K9me3 domains, as compared to WT (Fig. 4a, b, Additional file 3: Fig. S3B–D, Additional file 20: Table S2), while MACS2 analysis did not detect any significant peak enrichment (Fig. 4a, b, Additional file 19: Table S1, Additional file 20: Table S2 and Additional file 21: Table S3 and Additional file 4: Fig. S4). Because of differences in significance calling between domainograms and MACS peak calling, combined with a low degree of cross-reactivity of the anti-H3K9me3 antibody (Additional file 7: Fig. S7), this remaining signal could be due to H3K27me3 detection in this mutant genomic context devoid of H3K9me3. This would mean that PaKmt1 (i) is responsible for the wild-type distribution of H3K9me3 modifications at the specific locations described above and (ii) is the main *P. anserina* H3K9me3 methyltransferase. Unexpectedly, our data revealed variations of the H3K27me3 patterns in the Δ*PaKmt1* mutant background (Fig. 4a, b, Additional file 3: Fig. S3B–D, Additional file 4: Fig. S4 and Additional file 19: Table S1, Additional file 20: Table S2 and Additional file 21: Table S3). First, the average length of the H3K27me3 blocks was reduced by two thirds (Fig. 4b). Second, about 30% of the H3K27me3 peaks were missing, especially those located in the gene-rich blocks (Fig. 4a, Additional file 3: Fig. S3B–D, Additional file 4: Fig. S4 and Additional file 20: Table S2). Third, some the remaining H3K27me3 peaks were re-localized within TEs, where they replaced the missing H3K9me3 marks (Additional file 21: Table S3).

ChIP-seq experiments performed using the Δ*PaKmt6* mutant strains revealed that this deletion drastically reduced the overall H3K27me3 content (significant genome coverage dropped from 18.95 to 0.04%, (Fig. 4a, b, Additional file 3: Fig. S3B–D, Additional file 4: Fig. S4 and Additional file 19: Table S1, Additional file 20: Table S2 and Additional file 21: Table S3). To our surprise, we noticed that the loss of PaKmt6 severely impacted the amount of H3K9me3 marks, since in the Δ*PaKmt6* background, H3K9me3 genome coverage dropped from 2.94 to 0.18% (Fig. 4a, b, Additional file 3: Fig. S3B–D, Additional file 4: Fig. S4 and Additional file 19: Table S1 and Additional file 21: Table S3). This means that the diminution in H3K9me3 peaks was as strong as the diminution in H3K27me3 peaks in this mutant background. Nevertheless, the remaining H3K9me3 peaks in the Δ*PaKmt6* background were not displaced, nor were the missing H3K9me3 blocks replaced by H3K27me3 ones (Additional file 3: Fig. S3C, D, Additional file 9: Fig. S9 and Additional file 21: Table S3).

### Loss of H3K27me3 in Δ*PaKmt6* background modifies gene expression

**Fig. 5:**
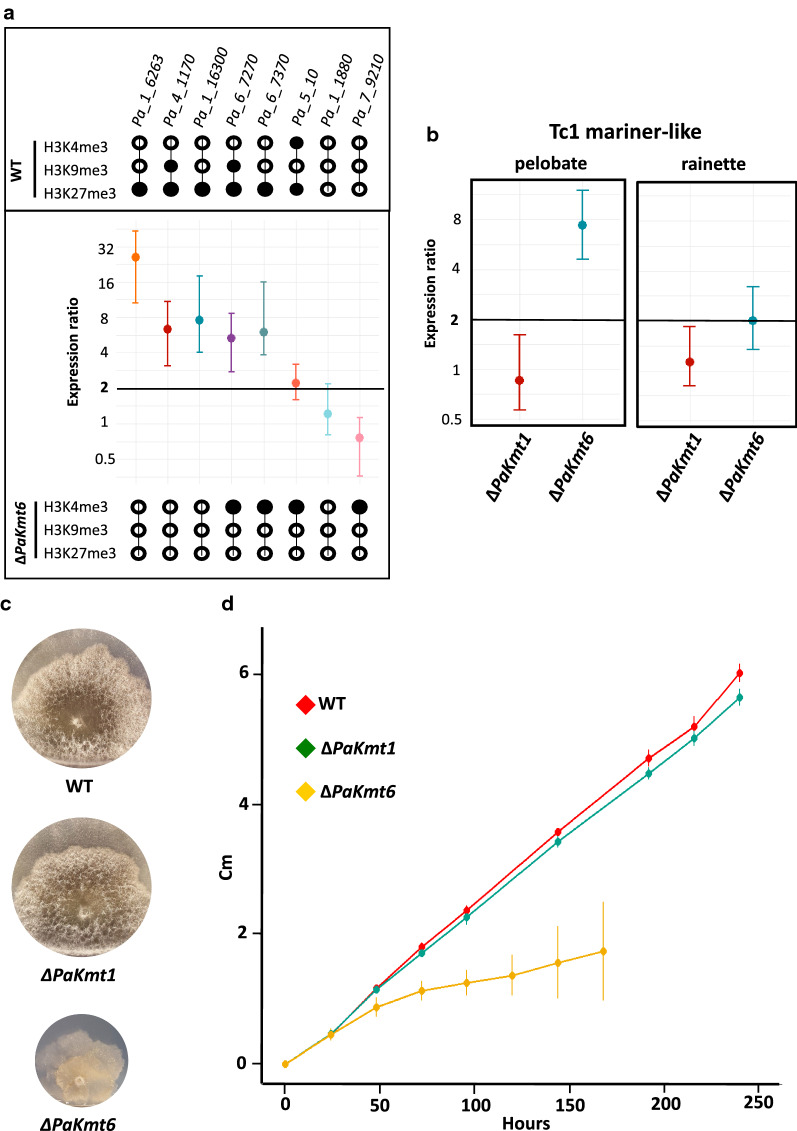
Gene expression in the Δ*PaKmt6* strain and growth features of the Δ*PaKmt6* and Δ*PaKmt1* strains. **a** Relative expression of selected genes in Δ*PaKmt6* mutant strains. Upper panel: histone marks in the wild-type strain. For each selected gene, a black dot shows the presence of the mark. Conversely a white dot shows its absence. The error bars represent the 95% confidence interval. *Pa_4_1170* and *Pa_1_16300* encode two putative Glycoside Hydrolases from families 7 and 61, respectively [51]. *Pa_6_7270* and *Pa_6_7370* belong to a secondary metabolite gene cluster located on a chromosome 7 sub-telomeric region. *Pa_5_10* encodes the meiotic drive element *Spok2* located on Chromosome 5 [56]. *Pa_1_1880* encodes the putative DNA repair RAD50 protein. *Pa_7_9210* encodes a putative exonuclease. They are both readily expressed at all stages of the life cycle and their expression remained unchanged in both of the two mutant backgrounds*.* Middle panel: normalized expression ratio of the selected genes relative to the wild-type strain. Overexpression (fold change ≥ 2, dark black line): *Pa_1_6263*: expression ratio = 25,987, *p*-value = 0.001; *Pa_4_1170*: expression ratio = 6375, *p*-value = 0.004; *Pa_1_16300*: expression ratio = 7588, *p*-value = 0.003; *Pa_6_7270*: expression ratio = 5280, *p*-value < 0001; *Pa_6_7370*: expression ratio = 6016, *p*-value = 0.002; *Pa_5_10*: expression ratio = 2212, *p*-value < 0.001; *Pa_1_1880*: expression ratio = 1.216, *p*-value = 0.192; Pa_7_9210: expression ratio = 0.756, *p*-value = 0.061. Three normalization genes (AS1, GPD and PDF2) were selected with geNorm [105] among eight housekeeping genes. Average expression stability of these three normalization genes is *M* = 0.230 and their pairwise variation is V3/4 = 0.071. See “Methods” for details and Additional file 22: Table S4 for Cq and selection of normalization genes and NRT-qPCR controls. Lower panel: histone marks in the Δ*PaKmt6* mutant strain. They are indicated as in the upper panel (see above). **b** Quantification of expression of two Tc1_mariner-like members in Δ*PaKmt1* and Δ*PaKmt6* mutant strains. Δ*PaKmt1* genetic context: no overexpression (fold change ≥ 2, dark black line). Δ*PaKmt6* genetic context: Tc1_mariner-like_pelobates family: expression ratio = 7.4, *p*-value = 0.009 in Δ*PaKmt6* genetic context and of Tc1_mariner-like_rainette family: expression ratio = 2.005, *p*-value = 0.004 in Δ*PaKmt6* genetic context. Normalization as described in **a**. **c** Phenotypic characterization of Δ*PaKmt1* and Δ*PaKmt6* single mutant strains. The *mat*+ strains of the indicated genotypes were grown on M2 minimal medium for 5 days at 27 °C. **d** Vegetative growth kinetics of Δ*PaKmt1* and Δ*PaKmt6* single mutant strains on M2 minimal medium. See “Methods” section for details

To assay the impact of H3K27me3 and/or H3K9me3 loss on gene expression, we performed RT-qPCR experiments on vegetative mycelia grown 60 h at 27 °C, for either the wild-type genotype (5 independent samples) or mutant genotypes (Δ*PaKmt1* and Δ*PaKmt6*, 5 independent samples each). We first focused on six genes that harbor H3K27me3 marks in wild-type background (Fig. 5a, Additional file 10: Fig. S10 and Additional file 20: Table S2). In the Δ*PaKmt6* background (Fig. 5a, Additional file 20: Table S2), all of them lost their H3K27me3 marks and were over-expressed (fold change ≥ 2). This result is consistent with H3K27me3 being involved in regulation of gene expression during development programs since *Pa_1_6263*, which showed the most dramatic up-regulation effect (fold change > 25, *p*-value = 0.001 Fig. 5a), is normally expressed only during the sexual phase [49]. Expression of genes encoding secondary metabolites (SM) is known to be sensitive to chromatin compaction [43, 44, 54, 55]. Indeed, when we assayed the expression of two such genes (*Pa_6_7270* and *Pa_6_7370*) in the Δ*PaKmt6* background, we found them both up-regulated (Fig. 5a, Additional file 20: Table S2). Notably, loss of PaKmt1 had no significant impact (fold change ≤ 2) on the expression of this set of genes, even if some of the H3K27me3 marks were lost in this mutant background (Additional file 10: Fig. S10 and Additional file 20: Table S2).

We also tested the expression of three TE families (Fig. 5b) in the wild-type background. The Copia_Ty1_nephelobates and the Tc1_mariner-like_pelobates are heavily RIPed and harbor both H3K9me3 and H3K27me3 marks (Additional file 21: Table S3 and Additional file 5: Fig. S5A, B). In consequence, their respective expression was silenced (Additional file 5: Fig. S5C and Additional file 22: Table S4). In contrast, the Tc1_mariner-like_rainette family shows fewer RIP mutations, along with H3K4me3 modifications but neither H3K9me3 nor H3K27me3 marks (Additional file 21: Table S3) and therefore was expressed (Additional file 22: Table S4). In the Δ*PaKmt6* background, expression of the Tc1_mariner-like_pelobates was significantly increased (fold change = 7.4, *p*-value = 0.009 Fig. 5b), while RT-qPCR was inconclusive for Copia_Ty1_nephelobates (i.e., RT-qPCR not different from NRT-qPCR, see “Methods” section). As expected, expression of the Tc1_mariner-like_rainette family was even further increased (fold change = 2.005 *p*-value = 0.004 Fig. 5b).

### Loss of PaKmt6 has stronger impact on vegetative growth than loss of PaKmt1 and results in male gamete over-production

We then assessed the impact of loss of either H3K27me3 or H3K9me3, or both, on the *P. anserina* life cycle.

Pigmentation, branching and the overall aspect of Δ*PaKmt1* mycelia were similar to those of wild-type strains, except for reduced production of aerial hyphae (Fig. 5c) and slight growth defects (Fig. 5d and Table 1), including an unusual failure to resume growth after it has been stopped for a few days (Additional file 11: Fig. S11A). We ruled out the possibility that these phenotypes result from activation of Crippled Growth (CG), an epigenetically triggered cell degeneration [57], by showing that the Δ*PaKmt1* mutation did not promote CG (Additional file 11: Fig. S11B) but behaved like the wild-type strain in that respect. This suggests that the reduced capability to resume growth may be indicative of less plasticity to perform an adaptive developmental program under changing environmental conditions.

**Table 1:** Mycelium growth at different temperatures

	11 °C	27 °C	35 °C
WT	100	100	100
ΔPaKmt1	72.4	94.2	91.8
ΔPaKmt1 PaKmt1^+^	104	100	99.7
ΔPaKmt6	ND	Irregular slow growth	Irregular slow growth
ΔPaKmt6 PaKmt6^+^	98.3	99.0	99.3
ΔPaKmt1 ΔPaKmt6	ND	Δ*PaKmt6* aggravated	Δ*PaKmt6*
ΔPaHP1	65.7	76.2	74.9
ΔPaHP1 PaHP1^+^	99.1	101	98.5
ΔPaKmt1 ΔPaPH1	60.7	89.9	80.7
ΔPaPH1 ΔPaKmt6	ND	Δ*PaKmt6* aggravated	Δ*PaKmt6*
ΔPaKmt1 ΔPaPH1 ΔPaKmt6	ND	Δ*PaKmt6* aggravated	Δ*PaKmt6*

Relative growth rates (WT %) at optimal temperature (27 °C) and sub-optimal temperature (11 °C and 35 °C) on M2 minimal medium. Δ*PaKmt6* mutant strains display irregular and slow growth at 27 °C and 35 °C, which cannot be quantified. At 11 °C, growth is too weak to be assayed (ND). At 27 °C, double and triple mutants containing the Δ*PaKmt6* allele display aggravated Δ*PaKmt6*-like growth defects

Pinkish pigmentation, crooked branching pattern and the overall aspects of Δ*PaKmt6* mycelia were clearly different from those of wild-type strains (Fig. 5c). First of all, Δ*PaKmt6* mutant growth rate was 43% lower than that of wild-type strains during the first 60 h of growth on M2 at 27 °C (Fig. 5d). After this time, progression of the Δ*PaKmt6* colony margins became so erratic and irregular that the standard assay consisting in thallus radius measurements was no longer accurate. Indeed, Δ*PaKmt6* mycelia showed “stop-restart” growth resulting in patchy mycelium sectors, which literally sprouted out of the colony margins like cauliflower inflorescences (Fig. 5c). These outgrowths are manifest in the increasingly large error bars along the corresponding growth curve (Fig. 5d). However, we were able to grow the Δ*PaKmt6* mutants in race tubes to determine that this mutation had no effect on senescence (Additional file 12: Fig. S12).

**Fig. 6:**
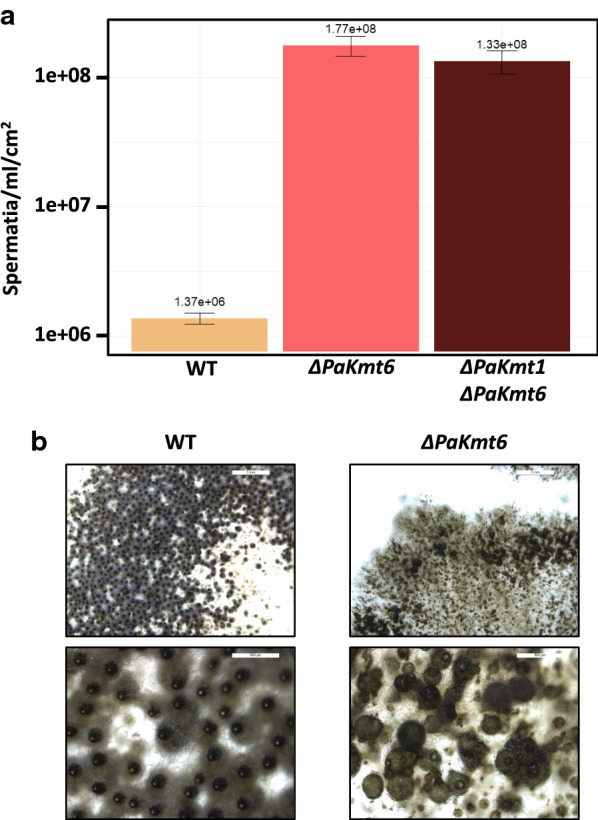
Sexual features of the Δ*PaKmt6* strain. **a** Spermatium production in wild-type and mutant backgrounds. See “Methods” section for details. **b** Δ*PaKmt6* homozygote crosses result in non-homogeneous distribution of late appearing fruiting bodies showing multiple morphological defects. These crippled structures could be due to lack of neck, lack of ostiols, lack of setae, or non-canonical pear-shape envelopes, supernumerary necks, misdirected orientation, upside down growth in agar, fusions of fruiting bodies, etc.

In addition to growth defects, the Δ*PaKmt6* mutant mycelium showed a powdery phenotype. At the microscopic scale, we observed that this corresponds to an over-production of male gametes (spermatia) (app. 130 times more than in the wild-type strains, Fig. 6a). We tested the ability of these Δ*PaKmt6* male gametes to fertilize wild-type female gametes (ascogonia). To do so, we sprayed a defined quantity of either mutant or wild-type male gametes onto wild-type female gametes of compatible mating type. Given that one fruiting body originates from one fertilization event, we established that the Δ*PaKmt6* male gametes were as efficient as the wild-type ones, since a similar number of fruiting bodies was obtained in each condition (4 independent experiments). This functional test indicates that the Δ*PaKmt6* mutant male gamete over-production had no obvious impact on their biological properties.

Finally, when we constructed the Δ*PaKmt1*Δ*PaKmt6* double mutant, the phenotypes generated by the Δ*PaKmt6* null allele were epistatic upon those generated by the Δ*PaKmt1* allele.

### The Δ*PaKmt6* mutant formed crippled fruiting bodies yielding a reduced number of ascospores

We then investigated the ability of the histone methyltransferase mutant strains to perform sexual reproduction. Sexual development and ascospore production of crosses involving two Δ*PaKmt1* parental strains were indistinguishable from those of wild-type strains. By contrast, when we performed homozygous Δ*PaKmt6* crosses, fruiting bodies were formed, which means that fertilization occurred, but their developmental time frame was delayed. They also displayed a large palette of morphological defects (e.g., lack of necks or extra necks, lack of ostioles, upward/downward mis-orientation, development into agar, fusion) and their ascospore production was significantly reduced (Fig. 6b).

Heterozygous crosses showed that when the Δ*PaKmt6* null allele was present in the female gamete genome and the wild-type allele was present in the male gamete genome, the fruiting bodies were crippled and the ascospore production reduced, as in the homozygous Δ*PaKmt6* crosses (Additional file 13: Fig. S13A). Conversely, heterozygous crosses involving female wild-type strains and male mutant strains produced well-formed and fully fertile fruiting bodies, indicating a Δ*PaKmt6* maternal effect.

To determine whether the Δ*PaKmt6* mutant’s reduced fertility resulted from either a fruiting body envelope defect or a zygotic tissue defect, we set up tricaryon crosses involving the Δ*mat* strain [58, 59]. The Δ*mat* strain lacks the genes required for fertilization, so it does not participate either as male or female in the sexual reproduction. However, the Δ*mat* mycelium is able to provide maternal hyphae required to build fruiting bodies. Consequently, the Δ*mat* strain can complement mutants defective for fruiting body tissue formation but not for zygotic tissue defect. In this study, we observed that the Δ*mat*; Δ*PaKmt6 mat*+; Δ*PaKmt6 mat*− tricaryon yielded few but fully matured and well-formed fruiting bodies that produced normal ascospores (Additional file 13: Fig. S13B). This experiment demonstrates that Δ*PaKmt6* mutation compromised the fruiting body envelope formation but had no impact on zygotic development. This result is in line with the observed Δ*PaKmt6* maternal effect (see the above paragraph).

Finally, crosses involving the double Δ*PaKmt1*Δ*PaKmt6* mutant strains displayed a typical Δ*PaKmt6* phenotype with respect to the perithecium number, morphology and ascospore production, demonstrating that the Δ*PaKmt6* null allele was epistatic. In addition, one third of the asci recovered from homozygous Δ*PaKmt1*Δ*PaKmt6* crosses showed fewer pigmented ascospores than asci from wild-type crosses (with a color panel spanning from white to green). In general, deficit in pigmentation is indicative of incomplete maturation during the ascospore formation process [60].

### The *PaKmt6* null allele reduces the ascospore germination efficiency

Homokaryotic ascospores originating from homozygous Δ*PaKmt1* crosses germinated like those of wild-type crosses (> 96% germination rate, *N* = 100, Table 2). However, about two thirds of normal looking ascospores produced by the homozygous Δ*PaKmt6* crosses did not germinate (3 independent experiments, *N* = 100 for each experiment, Table 2). The reduced germination efficiency was further enhanced in Δ*PaKmt1*Δ*PaKmt6* double mutants (Table 2). On germination medium, wild-type ascospore germination occurs at a germ pore located at the anterior side of the ascospore, by the extrusion of a spherical structure. This transient structure, called a germination peg, gives rise to a polarized hypha that develops into mycelium. In the Δ*PaKmt6* mutants, the ascospore germination process was stopped at an early stage, since germination pegs were not formed. However, the Δ*PaKmt6* defect was ascospore autonomous since ascospores from wild-type progeny obtained in WT × Δ*PaKmt6* crosses germinated normally, whereas those from Δ*PaKmt6* progeny did not. In addition, *PaKmt6*^+^/Δ*PaKmt6* dikaryotic ascospores had a wild-type germination rate showing that the Δ*PaKmt6* defect was recessive (2 independent experiments, *N* = 80 dikaryotic ascospores).

**Table 2:** Ascospore germination efficiency

	Germination efficiency
WT	92.8 ± 4.26
Δ*PaKmt1*	WT
Δ*PaKmt6*	29.3 ± 0.44
Δ*PaKmt6* *PaKmt6*^*+*^	WT
Δ*PaKmt1* Δ*PaKmt6*	11.4 ± 3.54
Δ*PaHP1*	WT
Δ*PaKmt1* Δ*PaHP1*	WT
Δ*PaHP1* Δ*PaKmt6*	25.2 ± 2.31
Δ*PaKmt1* Δ*PaHP1* Δ*PaKmt6*	21.1 ± 11.2

Number of germinated ascospores out of 100 issued from homozygote Δ*PaKmt6* crosses and transferred to appropriate germination medium. WT means wild-type germination efficiency (> 95%)

It was previously shown that unmelanized ascospores carrying the *pks1-193* mutant allele germinate spontaneously at high frequency (over 95%), even on non-inducing media [60, 61]. Crosses involving *pks1-193* Δ*PaKmt6 mat*- and *pks1*^+^ Δ*PaKmt6 mat*+ strains yielded asci made of two unmelanized (*pks1-193* Δ*PaKmt6*) and two melanized (*pks1*^+^ Δ*PaKmt6*) ascospores. As expected, about one third of the Δ*PaKmt6* melanized ascospores germinated on inducing medium. By contrast, almost all of the Δ*PaKmt6* unmelanized spores germinated, even on non-inducing medium. This result shows that the absence of melanin suppressed the Δ*PaKmt6* ascospore germination defect, as found for Δ*PaPls1* and Δ*PaNox2* mutants [60].

### Loss of PaHP1 leads to overall reduction and partial relocation of H3K9me3 modifications

**Fig. 7:**
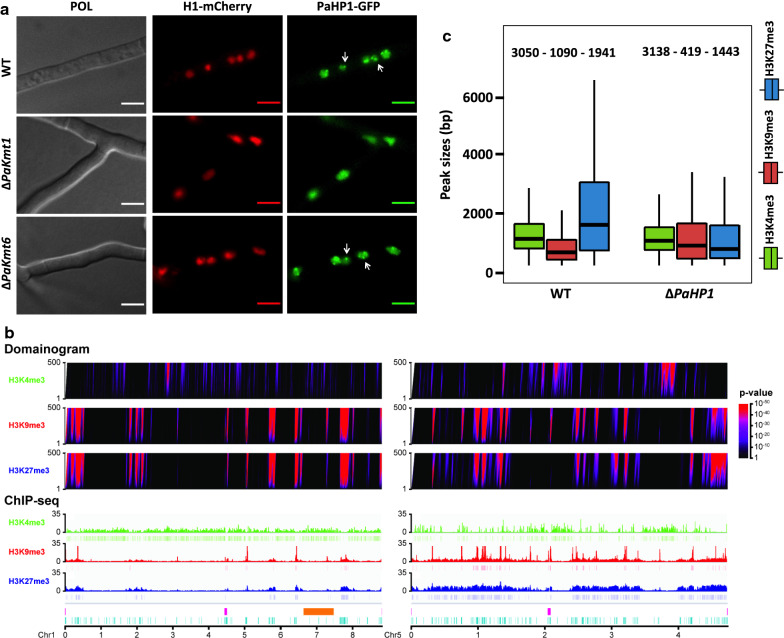
Chromatin modifications in the Δ*PaHP1* strain. **a** Subcellular localization of PaHP1. Fluorescence microscopy pictures show PaHP1-GFP chimeric protein during vegetative growth in mycelium. Nuclei are marked using the *PaH1-mCherry* allele that encodes histone H1 protein tagged with mCherry fluorophore (red). Indicated scale is the same for all the pictures (5 µm). PaHP1-GFP displays nuclear localization and forms foci that likely correspond to heterochromatin domains (arrows). This specific localization is disturbed only in Δ*PaKmt1* mutants. **b** Visualization of histone mark localization across the genome in the Δ*PaHP1 strain*. Domainograms (top) show significance of enrichment of H3K4me3, H3K9me3 and H3K27me3 marks in windows of varying size, according to the indicated *p*-value (right). ChIP-seq patterns (bottom) display histone modification coverage and MACS2 detected peaks. Both patterns are represented with the same scale on chromosomes one and five of *P. anserina.* Both centromeres are depicted (pink). The “Mat region” (orange) corresponds to the 800 kbp region devoid of recombination and containing the mating-type locus [93]. Repeated sequences are depicted in light blue. **c** Peak size distribution in the Δ*PaHP1* mutant strain. Box plots showing sizes in base pair of the MACS2-predicted peak of H3K4, H3K9 and H3K27 trimethylation are shown in green, red and blue, respectively. The box is delimited, bottom to top, by the first quartile, the median and the third quartile. Numbers on top of each box represents the number of peaks detected. Outliers are not shown

We searched the *P. anserina* genome for a homolog of heterochromatin protein 1 (HP1, [62]). This protein is known not only to bind the H3K9me3 marks of the constitutive heterochromatin, but also to perform a wide variety of functions ranging from cohesion of sister chromatids for the maintenance of telomeres, DNA repair and replication, cell cycle control and even RNA splicing [63]. We identified one gene (*Pa_4_7200*) encoding the protein PaHP1 (252 aa, 57% identity, e-value = 4e^−78^) (Additional file 14: Fig. S14A). It displays conserved chromo (PS00598) and chromo-shadow (PS50013) domains (Additional file 14: Fig. S14A, B and Additional file 15: Fig. S15A). By self-aggregation, chromo-shadow domain-containing proteins can bring together nucleosomes and, thus, condense the corresponding chromatin region [13]. RNA-seq data showed that the *PaHP1* gene is expressed in both vegetative mycelium and fruiting body tissues [49]. We also explored PaHP1-GFP cellular localization in 2-day-old growing mycelium. In wild-type PaHP1-GFP-expressing strains, the fluorescence signal was nuclear and punctate (Fig. 7a). However, when the *PaHP1-GFP* allele was expressed in a Δ*PaKmt1* background, the GFP fluorescence was still nuclear but no longer punctate (Fig. 7a), as already observed in *N. crassa* [48]. On the contrary, when expressed in a Δ*PaKmt6* background, the PaHP1-GFP signal appeared punctate as in wild-type nuclei.

To explore potential role(s) of PaHP1, we deleted the corresponding *Pa_4_7200* gene (Additional file 15: Fig. S15B, C). ChIP-seq experiments performed in the Δ*PaHP1* background showed that the H3K4me3 pattern was similar to that of wild-type strains (Fig. 7b, c, Additional file 3: Fig. S3B–D, Additional file 4: Fig. S4 and Additional file 19: Table S1 and Additional file 20: Table S2), while both H3K9me3 and H3K27me3 marks were reduced. The H3K9me3 genome coverage was reduced by half (Fig. 7c, Additional file 3: Fig. S3B–D, Additional file 4: Fig. S4 and Additional file 19: Table S1, Additional file 20: Table S2 and Additional file 21: Table S3), while the H3K27me3 loss of coverage was similar to that of Δ*PaKmt1* strains. Interestingly, on TEs, we detected replacement of some of the missing H3K27me3 by H3K9me3 modifications. This was especially noticeable for the soloLTR relics, although this was also the case for all the annotated TE families, albeit to a lesser extent (Additional file 21: Table S3). In addition, we observed both focalized spreads of H3K9me3 marks on TEs (Additional file 3: Fig. S3C, D, Additional file 21: Table S3) and massive H3K9 methylation on the rDNA cluster (Additional file 16: Fig. S16). These observations may account for the H3K9me3 block widening in the Δ*PaKmt1* genetic context. Altogether these results suggest that, in the absence of the recruitment platform mediated by the binding of PaHP1 to the constitutive heterochromatin, the compaction may be partly relaxed and the histone modifications lost, but also that the boundaries between heterochromatin and euchromatin may be less clear-cut. However, in this mutant context, the H3K9me3 reduction has no impact on gene expression (Additional file 17: Fig. S17A).

### Loss of PaHP1 has no more impact than that of PaKmt1 on the *P. anserina* life cycle

Throughout all the *P. anserina* life cycle, the Δ*PaHP1* mutants behave similarly to the Δ*PaKmt1* ones (Table 1, Additional file 17: Fig. S17B–D). Importantly, the double Δ*PaKmt1*Δ*PaHP1* mutants did not show any additional or more severe phenotypes (Table 1, Additional file 17: Fig. S17A). When the Δ*PaHP1* allele was associated with the Δ*PaKmt6* one, all the phenotypes displayed by the single Δ*PaKmt6* mutants were epistatic upon those of the Δ*PaHP1* mutants. The only exception is related to the germination efficiency of the triple Δ*PaKmt1*Δ*PaHP1*Δ*PaKmt6*, which was similar to that of the double Δ*PaHP1*Δ*PaKmt6* rather than the double Δ*PaKmt1*Δ*PaKmt6* mutant (Table 2). This may be due to a slightly reduced pigmentation of the ascospores produced by the homozygous Δ*PaKmt1*Δ*PaHP1*Δ*PaKmt6* crosses. Indeed, even if only the darker wild-type looking ones were used in this test, slightly reduced pigmentation facilitates germination.

## Discussion

In eukaryotes, the deposition of histone modifications is essential to chromatin features, which impact cell fate through regulation of gene expression. In this study, we aimed at understanding how heterochromatic territories are assembled within the *P. anserina* genome and how this genome organization influences the *P. anserina* life cycle.

### H3K27me3 and H3K9me3 modifications are not mutually exclusive

To get the first hints of *P. anserina*’s genome organization, we took advantage of ChIP-seq experiments to uncover genome-wide chromatin landscapes in a wild-type background. Our results highlighted two uncommon features of both the constitutive and the facultative heterochromatin. Firstly, in most animals and plants, the H3K9me3 modification covers fairly large domains, while the H3K27me3 modification forms shorter ones. But for the *P. anserina* genome, we observed the opposite, since H3K27me3 blocks were on average three times longer than H3K9me3 blocks. Second, if as expected the H3K9me3 modification was restricted to repeat-rich regions, mostly located near telomeres and centromeres [64], the H3K27me3 modification encompasses both annotated CDS and H3K9me3 marked repeats. Co-occurrence of H3K9me3 and H3K27me3 at same genomic loci is of particular interest because these two heterochromatic marks were long considered mutually exclusive in flowering plants, mammals, and fungi [30, 65]. Yet recently, this dogma has been challenged. Mass spectrometry experiments demonstrated that H3K27me3 and H3K9me3 can coexist within the same histone octamer in embryonic stem cells [35, 66]. Co-occurrence of H3K9me3 and H3K27me3 was found at TE loci of the ciliate *Paramecium tetraurelia* [67], the nematode *Caenorhabditis elegans* [68] and the bryophyte *Marchantia polymorpha* [69]. In mammals, these two marks were found together in rare instances, along with DNA methylation, either at a subset of developmentally regulated genes in mouse extra-embryonic early embryo lineages [70] or in some human cancer cells [71–73]. Likewise, such H3K9me3 and H3K27me3 overlap, exclusively associated with DNA methylation, has also been described for *A. thaliana* TE loci [74].

In the basidiomycete yeast *C. neoformans*, removal of the Enhancer-of-zeste-like protein EZH eliminates H3K27me3, while loss of its reader, the chromodomain Ccc1 protein, redistributes H3K27me3 to new genomic locations that coincide with H3K9me2 heterochromatin domains [41]. Further disruption of *C. neoformans* H3K9 methyltransferase Clr4 causes loss of both H3K9me2 and redistributed H3K27me3 modifications [41]. These findings suggest that co-factors, such as readers, are responsible for anchoring the H3K27 methyltransferase to its specific facultative heterochromatin compartments, that otherwise can be recruited to H3K9me constitutive heterochromatin, resulting in H3K9me/H3K27me co-occurrence. *P. anserina* has neither Ccc1 homologs nor a PRC1 complex. If such recognition modules were lost, it would explain why PaKmt6 is recruited by both facultative and constitutive heterochromatin. Interestingly, H3K9me3 and H3K27me3 modifications also overlap in the sub-telomeric regions of some other filamentous fungi, such as *Z. tritici*, *Fusarium oxysporum* and *Leptosphaeria maculans* ‘brassicae’ [32, 33, 38, 75–77].

### Are bivalent-like genes present in fungi?

In this study, we showed that H3K4me3 modification does not overlap with either of the H3 repressive marks at a genome-wide scale. However, we identified 50 genes (Additional file 20: Table S2) that make an exception to this rule by harboring both H3K4me3 and H3H27me3 marks. In mammals, this pattern is typical of bivalent genes found in totipotent embryonic stem cells [78], which play a central role in developmental and lineage specification processes. Unlike those of animals, fungal nuclei remain totipotent throughout the entire life cycle [79, 80]. Yet the molecular basis of this peculiar feature is not understood and it will be of interest to explore the function of the H3K4me3 and H3K27me3 dual-marked genes identified in the *P. anserina* genome (Additional file 20: Table S2). So far, we noted that a sizeable portion of them belongs to the *het* gene family. In filamentous fungi, the HET proteins trigger a cell death reaction as a consequence of a non-self-recognition process known as heterokaryon incompatibility [81]. It has been proposed that these *het* genes could be components of a fungal innate immune system. Since immune response must be quick and massive upon non-self-contacts, marking these genes with both repressive and activator modifications may be a way to fine tune their expression. Recently, a large number of genes associated with both H3K27me3 and H3K4me2 were identified in *L. maculans* ‘brassicae’ [77], indicating that bivalent domains occur in other fungi.

### PaKmt1 and PaKmt6 are not independent chromatin-modifying enzymes

ChIP-seq experiments carried out on mutants allowed us to test for potential cross-talk between constitutive and facultative heterochromatin. Our data established that *P. anserina’s* constitutive and facultative heterochromatin are inter-dependent since removal of either PaKmt1 or PaKmt6 impacted both marks although not to the same extent. This is in contrast with the corresponding mutant phenotypes reported so far in *N. crassa* [33, 35], *Z. tritici* [45] and *C. neoformans* [41] where H3K9me3 content is not affected by loss of H3K27me3 and conversely (Table 3). In addition, H3K27me3 reduction in Δ*PaKmt1* was not associated with any substantial genomic redistribution in contrast to what has been described in the corresponding *N. crassa* and *Z. tritici* mutants [33, 35, 45] (Table 3). Lack of PaHP1 further supported the observation that either type of heterochromatin requires the presence of the other, since in this mutant background, we saw fewer H3K9me3 and H3K27me3 marks but also reduced size of facultative heterochromatin domains. These concomitant observations suggest that initiation and propagation of the deposition of chromatin marks by the PRC2 complex are inter-dependent processes in *P. anserina*. Alternatively, if, as shown in *A. thaliana* for the FLC locus [89], that initiation and propagation of the deposition of chromatin marks by the PRC2 complex were independent in *P. anserina*, both of them would be impaired when the H3K9me3–heterochromatin pathway is damaged.

**Table 3:** Phenotypes associated with either loss of KMT1, KMT6 or HP1 in fungal species

Species	KMT1 mutant	KMT6 mutant	HP1 mutant	References
*Podospora anserina*				
Growth and development	Reduced production of aerial hyphae Slight growth defect Delay in resuming vegetative growth	Very poor growth Less pigmentation Overproduction of conidia Misshapped fructification Defect in spore germination	Reduced production of aerial hyphae Slight growth defect	This study
Chromatin and regulation ChIP-seq	Loss of H3K9me3	Loss of H3K27me3 and H3K9me3	Broader H3K9me3 domains	
*Neurospora crassa*				
Growth and development	Reduced growth Affected in sexual development	None	Reduced growth Affected in sexual development	Basenko et al. [34]; Freitag et al. [48]; Jamieson et al. [33, 35]; Klocko et al. [36]
Chromatin and regulation ChIP-seq and Hi-C	Affected in DNA methylation Relocalization of H3K27me3 Loss of H3K9me3 H3K27me3 relocation and diminution	Loss of H3K27me2/3 Altered in chromosome conformation	Affected in DNA methylation relocation of H3K27me2/3, superimposition with H3K9me3	
*Fusarium verticilloides*				
Growth and development	Defects in conidiation, perithecium production and fungal virulence Overpigmentation	N/A	N/A	Gu et al. [82]
Chromatin and regulation	N/A	N/A	N/A	
*Fusarium graminearum*				
Growth and development	N/A	Growth defects Sterility Overpigmentation Reduced virulence	Overpigmentation	Connolly et al. [43]; Reyes-Dominguez et al. [83]
Chromatin and regulation ChIP-seq	N/A	Loss of H3K27me3 Constitutive expression of genes encoding mycotoxins pigments-producing enzymes and other SM	Enhanced activity of activity of biosynthetic cluster genes	
*Epichloë festucae*				
Growth and development	Reduced vegetative growth Deregulation of symbiosis	Deregulation of symbiosis	Deregulation of symbiosis	Chujo and Scott [42]; Chujo et al. [84]
Chromatin and regulation ChIP-qPCR	Derepression of symbiosis-specific alkaloid genes clusters	Derepression of symbiosis-specific alkaloid genes clusters	Derepression of symbiosis-specific alkaloid genes clusters	
*Magnaporthe oryzae*				
Growth and development	Reduced vegetative growth Reduced virulence	Reduced vegetative growth Severe reduction in conidiation Slight defects in appressorium formation Reduction in pathogenicity	N/A	Pham et al. [46]
Chromatin and regulation WB	Loss of H3K9me3	Loss of H3K27me3	N/A	
*Zymoseptoria tritici*				
Growth and development	Reduced vegetative growth Reduced virulence	Reduced virulence	N/A	Möller et al. [45]
Chromatin and regulation ChIP-seq	Loss of H3K9me3 TE activation and genomic rearrangements H3K27me3 relocation Reduction of H3K27me3	Loss of H3K27me3 Increased stability of accessory chromosomes	N/A	
*Aspergillus nidulans*				
Growth and development	No developmental phenotype	N/A	No developmental phenotype	Reyes-Dominguez et al. [85]
Chromatin and regulation	Enhanced expression of a subset of secondary metabolite (SM) clusters	N/A	Enhanced expression of a subset of secondary metabolite (SM) clusters	
*Aspergillus fumigatus*				
Growth and development	Reduced vegetative growth delayed conidiation	N/A	Reduced vegetative growth and delayed conidiation	Palmer et al. [86]
Chromatin and regulation	N/A	N/A	N/A	
*Leptosphaeria maculans*				
Growth and development	Reduced vegetative growth Reduced virulence	N/A	Reduced vegetative growth	Soyer et al. [87]
Chromatin and regulation ChIP-seq (WT only)	Up-regulation of pathogenicity-related genes	N/A	Up-regulation of pathogenicity-related genes	
*Penicillium oxalicum*				
Growth and development	N/A	N/A	Reduced vegetative growth Delayed conidiation	Zhang et al. [88]
Chromatin and regulation	N/A	N/A	N/A	

Growth and development: macroscopic major defects caused by the specified mutant allele. Chromatin and regulation: H3K9me3 or H3K27me3 modification status in the mutant background and major impact on regulation of gene expression. Techniques used to characterize chromatin status are listed. ChIP-seq: chromatin immune-precipitation followed by sequencing, Hi-C: genome-wide chromosome conformation capture followed by sequencing, WB: Western blot

In general, two distinct enzymes catalyze methylation of either H3K9 or H3K27 residues. However, the ciliate *P. tetraurelia* is endowed with only one class of histone methlytransferase, the Enhancer-of-zeste-like protein Ezl1, which methylates both H3K9 and H3K27 residues [67]. This observation suggests that eukaryotes initially acquired a single, versatile, methyltransferase that duplicated and became sub-functionalized in the course of evolution. Thus many taxa have two enzymes, with distinct substrate specificities. This ancestral versatility might be partially restored (or unmasked) by the lack of competition when one of these enzymes is missing as in the Δ*PaKmt1* and Δ*PaKmt6* mutant genetic contexts. Under this hypothesis, we can propose that (i) the *P. anserina* SU(VAR)3-9-like homolog (PaKmt1) may have a residual H3K27 histone-methyltransferase activity, that would account for the remaining H3K27me3 marks in the Δ*PaKmt6* mutants, whereas (ii) SET-domain Enhancer-of-zeste-like PaKmt6 cannot be a catalytic substitute for PaKmt1, since H3K9me3 peaks were no longer detected in the Δ*PaKmt1* mutants.

### Constitutive chromatin is dispensable whereas facultative chromatin is required for most *P. anserina *developmental programs

Assessing the phenotype of Δ*PaKmt1*, Δ*PaHP1*, and Δ*PaKmt6* single mutants, we established that the loss of both PaKmt1 and PaHP1 has almost no effect on the *P. anserina* life cycle, whereas the absence of PaKmt6 drastically impairs every developmental step. To recapitulate, the Δ*PaKmt6* inactivation resulted in (i) slow, irregular, and thermo-sensitive mycelium growth, (ii) absence of aerial hyphae, (iii) striking over-production of male gametes, (iv) reduced fertility of the female gametes and/or of fertilization efficiency, (v) formation of crippled and mis-orientated fruiting bodies, (vi) reduced yields of ascospores and (vii) reduced germination efficiency of ascospores (Table 3). This means that almost every aspect of the life cycle is impacted by the loss of most of H3K27me3 marks and consequently of the facultative heterochromatin (see below). By contrast, the single Δ*PaKmt1* and Δ*PaHP1* mutants showed only marginal defects on aerial hyphae production and resumption of growth after a period of arrest (Table 3). More importantly, the single Δ*PaKmt1* and Δ*PaHP1* and double Δ*PaKmt1* Δ*PaHP1* mutants are fully fertile. These phenotypes contrast with those of *N. crassa*, as well as most filamentous fungi studied to date, i.e., *Aspergillus fumigatus*, *E. festucae*, *L. maculans*,* Penicillium oxalicum*, and *Z. tritici* (Table 3). Conversely, for *F. graminearum* and *F. fujikuroi*, Enhancer-of-zeste-like enzymes are key for their developmental integrity, as in *P. anserina* (Table 3). *F. graminearum kmt6* mutants show aberrant germination patterns, over-pigmented mycelium, restricted growth, reduced pathogenicity, over-production of several SM clusters, and sterility, while loss of Kmt1 or HP1 orthologues does not lead to any noticeable phenotypes [43]. Similarly, *F. fujikuroi kmt6* mutants form stunted mycelia that produce a reduced yield of conidia [44].

## Conclusions

Recent studies (Table 3) suggest that the canonical definitions of facultative and constitutive heterochromatin may be challenged in fungi. Further contributing to this complex picture, in this study we showed that besides the H3K9me3 modifications that are likely installed by RIP [90], *P. anserina* repeats also harbor H3K27me3 modifications. It has already been postulated that the ancestral role of Enhancer-of-zeste enzymes, along with the SET-domain SU(VAR)3–9 enzymes, may be to silence TEs, through the concerted action of both H3K27me3 and H3K9me3 [67, 69, 91]. The H3K9me3/H3K27me3 overlap we have observed could therefore be a relic of the concerted action of these two classes of histone methyltransferases. Recently, a BAH-PHD protein mediating Polycomb silencing [40] and an ISWI chromatin remodeling factor were identified in *N. crassa* [92]. Interestingly, the phylogenetic distribution of this BAH-PHD protein suggests that it belongs to an ancestral Polycomb repression system which still exists in Fungi and early emerging eukaryotes but no longer in animals. The search for *P. anserina* PRC2 associated proteins will help us to understand Polycomb silencing in the absence of canonical PRC1, as well as functional cross-talk between facultative and constitutive heterochromatin.

## Methods

### Strains and culture conditions

The strains used in this study derived from the “S” wild-type strain of *P. anserina* that was used for sequencing [51, 93]. Standard culture conditions, media and genetic methods for *P. anserina* have been described [94, 95]. Most recent protocols can be accessed at either [96] or http://podospora.i2bc.paris-saclay.fr. Mycelium growth is performed on M2 minimal medium, in which carbon is supplied as dextrin and nitrogen as urea. Ascospores germinate on a specific germination medium (G medium) containing ammonium acetate. The methods used for nucleic acid extraction and manipulation have been described [97, 98]. Transformations of *P. anserina* protoplasts were carried out as described previously [99].

### Identification and deletions of the *PaKmt1*, *PaKmt6* and *PaHP1* genes

*PaKmt1*, *PaKmt6 and PaHP1* genes were identified by searching the complete genome of *P. anserina* with tblastn [100], using the *N. crassa* proteins DIM-5 (NCU04402), SET-7 (NCU07496) and HP1 (NCU04017). We identified three genes: *Pa_6_990*, *Pa_1_6940* and *Pa_4_7200* renamed *PaKmt1*, *PaKmt6 and PaHP1,* respectively. To confirm gene annotation, *PaKmt1* transcripts were amplified by RT-PCR experiments performed on total RNA extracted from growing mycelia (1-day- and 4-day-old mycelium) and developing fruiting bodies (2 and 4 days post-fertilization) using primers 5s-DIM5 and 3s-DIM5 (Additional file 23: Table S5). Functional annotation was performed using InterProScan analysis (http://www.ebi.ac.uk/interpro/search/sequence-search), Panther Classification System (http://www.pantherdb.org/panther/), PFAM (http://pfam.xfam.org/) and Prosite (http://prosite.expasy.org/).

Deletions were performed on a Δ*mus51::bleoR* strain lacking the mus-51 subunit of the complex involved in end-joining of DNA double strand breaks as described in [60]. In this strain, DNA integration mainly proceeds through homologous recombination. Replacement of the *PaKmt1* wild-type allele with hygromycin-B resistance marker generated viable mutants carrying the Δ*PaKmt1* null allele. Replacement of the *PaKmt6* wild-type allele with a nourseothricin resistance marker generated viable but severely impaired mutants. Replacement of the *PaHP1* wild-type allele with a hygromycin-B resistance marker also generated viable mutants carrying the Δ*PaHP1* null allele. All deletions were verified by Southern blot (Additional file 8: Fig. S8 and Additional file 12: Fig. S12A).

### Construction of fluorescent-HA-tagged chimeric proteins *PaHP1-GFP-HA, PaKmt1-mCherry-HA, PaKmt6-GFP-HA*

The pAKS106 and pAKS120 plasmids were described in [101]. All the cloned inserts were sequenced before transformation.

To gain insight into PaHP1 localization, we constructed a PaHP1-GFP-HA fusion chimeric protein. To this end, the *PaHP1* gene was PCR amplified from the S strain genomic DNA using primers FC3-BamH1 and FC4-GFP (Additional file 23: Table S5). The resulting amplicon harbors 520 bp of promoter sequence followed by *PaHP1* CDS cleared from its stop codon. In parallel, *eGFP* CDS was amplified from peGFP plasmid using FC5-HP1 and FC6-HindIII primers. The two DNA segments were fused by PCR using primers FC3-BamH1 and FC6-BamH1. This chimeric DNA fragment was digested with BamHI and HindIII and cloned into the pAKS106 plasmid linearized with the same enzymes. When transformed into Δ*PaHP1* mutant strain, the *PaHP1-GFP-HA* allele is able to restore a wild-type phenotype (growth rate and aerial mycelium), showing that the tagged version of PaHP1 protein is functional.

*PaKmt1-mCherry-HA* allele construction followed the same experimental design used for *PaHP1-GFP-HA* construction. First, *PaKmt1* was PCR amplified using Dim5mChFBamH1 and Dim5mChR and resulted in a DNA amplicon composed of 454 bp of promoter followed by the *PaKmt1* CDS. The *mCherry* CDS was amplified from the pmCherry plasmid using mChDim5F and mChDim5RHindIII primers. Fragments were fused by PCR, digested and cloned in pAKS106. The *Kmt1-mCherry-HA* allele was able to restore wild-type growth phenotype when transformed in Δ*PaKmt1* mutant strains, indicating that this chimeric protein is functional.

*PaKmt6* (*Pa_1_6940*) was PCR amplified from S genomic DNA using FC73-GA1KMT6 and FC74-GA1KMT6 primers, resulting in 4232 bp DNA amplicons composed of 912 bp of promoter followed by *PaKmt6* CDS cleared from its stop codon. In parallel, the *GFP* allele was amplified from peGFP plasmid using FC75-GA1GFP and FC76-GA1GFP. Both fragments were cloned together in one step in the pAKS-106 plasmid previously digested with NotI and BamHI restriction enzymes. This cloning step was performed using Gibson Assembly kit (New England Biolabs). This yielded the chimeric *PaKmt6-GFP-HA* allele. An additional *PaKmt6-HA* allele was generated by PCR amplification from the wild-type strain DNA of *PaKmt6* CDS along with its own promoter, using FC65-NotI and FC72-BamHI primers. These amplicons were digested and cloned in pAKS106 plasmid using NotI and BamHI restriction enzymes. When transformed into Δ*PaKmt6* mutant strain, both *PaKmt6-GFP-HA* and *PaKmt6-HA* alleles were able to restore wild-type growth and sexual development, suggesting that this chimeric version of the PaKmt6 protein is functional. However, no GFP fluorescence signal was detected in the Δ*PaKmt6*, *PaKmt6-GFP-HA* complemented strains.

To overexpress *PaHP1*, the endogenous promoter were replaced by the *AS4* promoter that drives high and constitutive expression throughout the life cycle [102]. *PaHP1-GFP* allele was PCR amplified from pAKS106-*HP1-GFP* (this study) using primers FC21-BamH1 and FC6-HindIII. The resulting 1877 bp amplicons harbor the chimeric construction cleared from its stop codon and promoter. The PCR product was digested with BamHI and HindIII and then cloned in pAKS120 previously digested with the same enzymes. The *pAS4-PaHP1-GFP-HA* resulting allele was introduced in Δ*PaHP1* strains by transformation.

*pAS4-PaKmt1-mCherry-HA* over-expressed allele construction followed the same experimental procedures as the one used for *pAS4-HP1-GFP-HA* construction. *PaKmt1-mCherry* CDS was PCR amplified using FC22-BamHI and mChDim5R-HindIII using *pAKS106-PaKmt1-mCherry-HA* plasmid as DNA template. Amplified DNA fragments were then digested and cloned in pAKS120 plasmid using HindIII and BamHI restriction enzymes. This allele was transformed in Δ*PaKmt1* strain.

### Complementation experiments with GFP-tagged proteins

To perform complementation experiments, the *PaKmt1*-*mCherry* allele was transformed into the Δ*PaKmt1* strain. Because the *PaKmt1*-*mCherry* allele was linked to a phleomycin marker, 55 phleomycin-resistant transformants were recovered. Among those, five wild-type looking independent transgenic strains were selected and crossed to a wild-type strain. In the progeny, all the phleomycin- and hygromycin-resistant haploid strains behaved similarly to the wild-type strain. Two of these independent transformants were selected for further phenotypic characterization. To ask whether a constitutively over-expressed *PaKmt1* allele could have an impact on the physiology of *P. anserina*, we introduced the *AS4-PaKmt1*-*mCherry* allele into the Δ*PaKmt1* strain. All the phleomycin-resistant transformants looked like a wild-type strain (*N* = 30 phleomycin resistant). Two of them were crossed to a wild-type strain. In their progeny, all the phleomycin-resistant strains carrying either the *PaKmt1*^+^ allele or the Δ*PaKmt1* were indistinguishable from wild-type strains.

Similarly, we transformed a *PaHP1*-*GFP-HA* allele, linked to a phleomycin marker, into a mutant Δ*PaHP1* strain. Among the 20 phleomycin-resistant transformants that were recovered, five independent transformants displaying a wild-type phenotype were selected and crossed to the wild-type strain. In the progeny, all the phleomycin- and hygromycin-resistant haploid strains behaved similarly to the wild-type strain. Transformation of the *AS4-PaHP1-GFP-HA* allele into the Δ*PaHP1* strain generated phleomycin-resistant transformants (*N* = 19) showing intermediate to full complementation but no extra phenotypes. Two transformants presenting full complementation were crossed to a wild-type strain. In their progeny, all the phleomycin-resistant strains carrying either the *PaHP1*^+^ allele or the Δ*PaHP1* behaved like the wild-type strains.

Finally, the *PaKmt6-HA* allele and the *PaKmt6*-GFP*-HA* allele were independently transformed into a Δ*PaKmt6* strain. For the two transformation experiments, among the phleomycin-resistant transformants that were recovered (*N* = 20 and *N* = 32, respectively), two independent transgenic strains displaying a wild-type phenotype were selected and crossed to a wild-type strain. In the progeny of these four crosses, all the phleomycin- and nourseothricin-resistant haploid strains behaved similarly to wild-type strain.

### Phylogenetic analysis

Orthologous genes were identified using the MycoCosm portal [103] and manually verified by reciprocal Best Hit BLAST analysis. Sequences were aligned using Muscle (http://www.ebi.ac.uk/Tools/msa/muscle/) and trimmed using Jalview (version 2.9.0b2) to remove non-informative regions (i.e., poorly aligned regions and/or gap containing regions). Trees were constructed with PhyML 3.0 software with default parameters and 100 bootstrapped data sets [104]. The trees were visualized with the iTOL server (http://itol.embl.de/).

### Phenotypic analyses

Spermatium counting was performed as follows: each strain was grown on M2 medium with two cellophane layers at 18 °C for 14 days (P. Silar’s personal communication). To collect spermatia, cultures were washed with 1.5 mL of sterile water. A Malassez counting chamber was used for enumeration.

### Cytological analysis

Light microscopy was performed on 2-day-old mycelium. Explants taken from complemented strains expressing *PaHP1-GFP-HA* were analyzed by fluorescence microscopy. Pictures were taken with a Leica DMIRE 2 microscope coupled with a 10-MHz Cool SNAPHQ charge-coupled device camera (Roper Instruments), with a z-optical spacing of 0.5 mm. The GFP filter was the GFP-3035B from Semrock (Ex: 472 nm/30, dichroïc: 495 nm, Em: 520 nm/35). The Metamorph software (Universal Imaging Corp.) was used to acquire z-series. Images were processed using the ImageJ software (NIH, Bethesda).

### Western blot analysis

Western blots were performed on nuclear extracts made from 200 mg of ground mycelia resuspended in 3 mL of New Cell Lysis Buffer [Tris–HCL pH 7.5 50 mM, NaCl 150 mM, EDTA 5 mM, NP-40 0.5%, Triton 1% and protease inhibitor (Roche-04693132001)]. The nuclear extracts were collected after centrifugation (300×*g*, 5 min) and resuspended in 300 µL of Nuclei Lysis Buffer (Tris–HCl pH 8 50 mM, EDTA 10 mM, SDS 0.5% and protease inhibitor (Roche-04693132001). Nuclei were then sonicated (Diagenode bioruptor plus sonicator, 20 min at high level). Protein contents of nuclear extracts were quantified using the Qubit Protein assay kit (Invitrogen). Then, 35 µg of nuclear extract were diluted in Laemmli sample buffer and loaded on a 10% SDS polyacrylamide gel. Membranes were probed using the following high affinity ChIP-grade monoclonal antibodies: anti-Histone 3 (Abcam ab12079), anti-H3K9me3 (Abcam ab8898), anti-H3K27me3 (Millipore 17-622) and anti-H3K4me3 (Active Motif 39159).

### Chromatin immuno-precipitation

Each ChIP-seq experiment was performed on two independent biological replicates for each genotype (*mat*+ strains only). Chromatin extractions were made from mycelium grown for 60 h at 27 °C in liquid culture (KH_2_PO_4_ 0.25 g/L, K_2_HPO_4_ 0.3 g/L, MgSO_4_ 0.25 g/L, urea 0.5 g/L, biotin 0.05 mg/L, thiamine 0.05 mg/L, oligo-element 0.1%, yeast extract 5 g/L and dextrin 5.5 g/L). Mycelium was filtered and quickly washed with phosphate-buffered saline (PBS), resuspended and then cross-linked in 100 mL of PBS with 1% paraformaldehyde for 15 min at 27 °C. Reaction was stopped by addition of glycine (60 mM). Cross-linked mycelium was filtered, washed with cold PBS buffer and dried on Whatman paper. Dried mycelium was frozen in liquid nitrogen and ground to powder in a mortar. Aliquots of mycelium powder (200 mg) were resuspended in 1 mL of Lysis Buffer [Hepes pH 7.5 50 mM, NaCl 0.5 M, EDTA pH 8 1 mM, Triton X-100 1%, Sodium deoxycholate 0.1%, CaCl_2_ 2 mM and protease inhibitor (Roche)]. Chromatin was digested to 0.15–0.6-kb fragments using Micrococcal nuclease (NEB #M0247) for 30 min at 37 °C (10,000 gels units/mL). The enzymatic digestion was stopped by addition of EGTA (15 mM). After centrifugation (15,000×*g*, 15 min, twice) of the digested samples, pelleted genomic DNA fragments were discarded. Concentration of the soluble chromatin fractions was assayed using the fluorescence system Qubit dsDNA HS assay kit (Invitrogen). Prior to immune-precipitation, 5 µg of soluble chromatin was washed in 1.1 mL of Lysis buffer containing 30 µL of magnetic beads (Invitrogen 10001D, 4 h at 4 °C). From the 1.1-mL washed soluble chromatin cleared from magnetic beads, 100 µL was kept for non-IP input control. Specific antibodies were added individually to the remaining 1 mL of the washed soluble chromatin (IP samples) for an overnight incubation at 4 °C on a rotating wheel. Antibodies used in this study are anti-H3K9me3 (Abcam ab8898), anti-H3K27me3 (Millipore 17-622) and anti-H3K4me3 (Active Motif 39159). One additional mock sample was done with an anti-GFP antibody (Abcam ab290) in the wild-type strain only. Chromatin was immuno-precipitated by adding 20 µL of magnetic beads, incubated for 4 h at 4 °C on a rotating wheel. Beads were successively washed in 1 mL for 10 min in of the following buffers: Lysis buffer (twice), Lysis buffer plus 0.5 M NaCl, LiCl Wash Buffer (Tris–HCl pH 8 10 mM, LiCl 250 mM, NP40 0.5%, Sodium deoxycholate 0.5%, EDTA pH 8 1 mM; twice for H3K9me3 immuno-precipitation) and finally Tris–EDTA Buffer (Tris–HCl pH 7.5 10 mM, EDTA pH 8 1 mM). Elution of IP samples was done with TES buffer (Tris–HCl pH 8 50 mM, EDTA pH 8 10 mM with SDS 1%) at 65 °C. TES buffer was also added to non-immuno-precipitated input sample. Both IP and input samples were decrosslinked at 65 °C overnight and treated successively with RNAse A (Thermo Fisher Scientific) (0.2 mg/mL, 2 h at 50 °C) and Proteinase K (Thermo Fisher Scientific) (0.8 mg/mL, 2 h at 50 °C). DNA was then subjected to phenol–chloroform purification and ethanol precipitation. DNA pellets were resuspended in 30 µL of Tris–EDTA buffer.

### RT-qPCR experiments

Cultures for RNA extractions were performed for 60 h at 27 °C in liquid culture (KH_2_PO_4_ 0.25 g/L, K_2_HPO_4_ 0.3 g/L, MgSO_4_ 0.25 g/L, urea 0.5 g/L, biotin 0.05 mg/L, thiamine 0.05 mg/L, oligo-element 0.1%, yeast extract 5 g/L and dextrin 5.5 g/L). For each replicate, 100 mg of mycelium was harvested, flash-frozen in liquid nitrogen and ground with a Mikro-Dismembrator S (Sartorius, Göttingen, Germany) for one minute at 2600 rpm in a Nalgene Cryogenic vial (ref # 5000-0012, ThermoFischer Scientific, Waltham, USA) with a chromium steel grinding ball (ref # BBI-8546916, Sartorius, Göttingen, Germany). Total RNAs were extracted with the RNeasy Plant Mini Kit (ref # 74904, Qiagen, Hilden, Germany), according to the protocol described for Plants and Fungi with buffer RLT. Contaminating DNA was digested in RNA solutions with RNase-free DNase (ref # 79254, Qiagen, Hilden) and RNAs were purified once more with the RNeasy Plant Mini Kit (ref # 74904, Qiagen, Hilden, Germany), according to the protocol described for RNA cleanup. RNAs were quantified with a DeNovix DS-11 spectrophotometer (Willmington, USA), checked for correct 260/280 and 260/230 ratios, and RNA quality was checked by gel electrophoresis. Reverse transcription was performed with the SuperScript III Reverse Transcriptase (ref # 18080093, Invitrogen, Carlsbad, USA) and oligo-dT. A non-reverse transcribed (NRT) control was systematically performed on a pool of NRT controls to ensure that the Cq was above the Cq obtained from corresponding reverse transcribed RNAs. For genes without introns, NRT control was performed for each replicate sample to ensure that the Cq was above the Cq obtained from corresponding reverse transcribed RNAs. Experiments were run with five biological replicates for each strain, except for some TE cDNA detections (see the section below) (see Additional file 22: Table S4 for Cq). Each biological replicate was run in technical triplicates (see Additional file 22: Table S4 for Cq), except NRT control, which were in technical duplicates (see Additional file 22: Table S4 for Cq). When possible, primers were designed on two consecutive exons (Additional file 23: Table S5). Normalization genes were selected among a set of 8 housekeeping genes using geNorm (see Additional file 21: Table S3 for details of analysis) [105]. Selected normalization genes are *AS1* (Pa_1_16650), *GPD* (Pa_3__5110) and *PDF2* (Pa_7_6690), with an average expression stability *M* = 0.23 indicating a homogeneous set of samples and a V3/4 < 0.15 (see Additional file 22: Table S4 for details of analysis). RT-qPCR normalization according to the ΔΔCt method [106], standard error and 95% confidence interval calculations, and statistical analyses were performed using REST 2009 software (Qiagen, Hilden, Germany). Genes were defined as down-regulated in the mutant strain if the ratio of their transcript level in the mutant strain compared with that in the wild-type strain showed a-fold change > 0 and < 1, with a *p*-value < 0.05. On the other hand, genes were defined as up-regulated in the mutant strain if the ratio of their transcript level in the mutant strain compared with that in the wild-type strain showed a > onefold change, with a *p*-value < 0.05. Ratios with a 95% confidence interval, including 1, were not considered significant [107]. RT-qPCR experiments were MIQE compliant [108].

### RT-qPCR experiments for detection of TE

Primers for detection of TE were designed to detect as many members as possible for each family (Additional file 23: Table S5). These primer couples detect genomic DNA as well as cDNA. As described above, NRT-qPCR controls were performed with these primer couples on each biological replicate and revealed for some of them that NRT-qPCR signals were too close to the RT-qPCR signals to allow a reliable analysis of the data. Biological replicates that displayed NRT-qPCR signals less than 2.9 cycles above the RT-qPCR signal were discarded and analyses were performed if three biological replicates at least were available (see Additional file 22: Table S4 for details of analysis).

### qPCR analysis to test ChIP efficency

Quantitative PCR experiments were performed with dsDNA fluorescent detection method using the FastStart Universal SYBR Green Master from Roche (04913850001).

IP enrichments were assayed with the following protocol: 5 µL of IPed DNA samples and non-IPed input were diluted 10 times in nuclease free water. qPCR experiments were done on 2 µL of the diluted samples using the pairs of primers (0.5 µM) FC77-Actin/FC78-Actin and FC125-580/FC126-580 (see Additional file 23: Table S5). For each IP sample, relative concentrations were determined as an “Input percentage” with the following equation:

$$\% {\text{Input}} = \left( {{\text{E}}^{{\left( { {\text{CqInput}} {-}\left( {\frac{{\ln \left( {10} \right)}}{{{\text{lnE}}}}} \right)} \right) - {\text{CqSample}}}} } \right) \times 100.$$

### Sequencing

ChIP-seq libraries were built using the NEB Next UltraII DNA library Prep kit for Illumina (New England Biolabs #E7645S/L) and Agencourt Ampure XP beads (Beckman Coulter, #A63880) according to the manufacturer’s protocols. PCR amplification was made of 12 cycles. ChIP-sequencing was performed by the I2BC High-throughput sequencing facility [NextSeq 500/550 High Output Kit v2 (75 cycles), SR 75 bp]. Reads were trimmed with Cutadapt 1.15 and filtered for control quality by FastQC v0.11.5.

### ChIP-seq sequencing data analysis and visualization

Sequencing generated between 8 and 45 million reads depending on samples. The ChIP-seq analysis was performed on sets of data that contained 8 to 10 million reads at most. If needed, the number of reads was down-sized randomly. Reads were mapped on the S *mat*+ *P. anserina* genome [51] using Bowtie 2 software (version 2.3.0, see annex for results). For visualization, data were normalized using Deeptools 2.0 [109] bamcoverage with the BPM method. Spearman correlation factors were calculated on normalized data using Deeptools 2.0. For each condition, we made sure that the two biological replicates correlate (Additional file 14: Fig. S14 and Additional file 25: Table S7 and Additional file 26: Table S8) and then merge them for peak calling. Peak calling was performed using MACS2 software (version 2.1.1) using the mock sample as control. Further peak annotations and comparisons were done using Bedtools (version 2.26.0). Genome-wide visualization of peaks was also generated with Circos software [110]. All graphs have been drawn using ggplot2 [111]. Heatmap and metaplot figures have been generated using Deeptools2. Genomic regions have been extracted from the current genome annotation. Promoters have been arbitrarily defined as 1 kb 5′ of the start codon. Scores of the heatmap represent the mapped reads after normalization (see above), hence not only the MACS2-predicted peaks. The Euler diagram has been generated by the eulerr R package (v 6.0.0, https://cran.r-project.org/package=eulerr). The domainogram analysis [50] has been performed using scripts described in [112] and downloaded from the BBCF Github (EPFL, Lausanne, https://github.com/bbcf/). The domainograms were calculated on binned data sets (100 bp binning), using windows sizes ranging from 1 to 500 bins. Individual BRICKs (Blocks of Regulators In Chromosomal Kontext), which represent regions with significant clustering of ChIP-seq signal, were limited to 50 bins each.

To assess whether any of the biological functions observed in H3K4me3 + H3K27me3 gene lists (Additional file 20: Table S2) were present at a frequency greater than that expected by chance, *p*-values were calculated using hypergeometric distribution as described in [113].

## Supplementary Information

**Additional file 1: Figure S1.** Conserved structure and phylogenetic analysis of histone methyltransferases involved in heterochromatin assembly. Domain structure comparison of histone methyltransferase Kmt1 (**A**) and Kmt6 (**B**). Sizes in amino acid (aa) are given (right). Pre-SET (red, IPR007728), SET (orange, IPR001214) and Post-SET (green, IPR003616) conserved domains are required for H3K9 methyltransferase activity of Kmt1 homolog proteins. SRA (SET and RING finger-associated, purple, IPR003105) and SANT (blue, IPR001005) are protein–protein interaction domains. CXC (yellow, IPR026489) is a cysteine-rich conserved domain located in the H3K27 methyltransferase catalytic domain of Kmt6 homologs. Filamentous fungi: *Podospora anserina* (Pa), *Neurospora crassa* (Nc), *Fusarium graminearum* (Fg), *Epichloë festucae* (Ef), *Aspergillus nidulans* (An); yeasts: *Schizosaccharomyces pombe* (Sp) and *Cryptococcus neoformans* (Cn); the worm *Caenorhabditis elegans* (Ce), the fruit-fly *Drosophila melanogaster* (Dm), the mouse *Mus musculus* (Mm) and the model plant *Arabidopsis thaliana* (At). Accession numbers for proteins used in alignments are listed in Additional file 24: Table S6. **C** Alignment of the SET domains within SU(VAR)3–9 (upper panel) and EZH2 (lower) homologs for a set of selected species. Deleted parts of proteins are indicated by // (shaded in gray). Conserved GxG motif (shaded in green) corresponds to the substrate AdoMet/SAM binding site (motif I). Motif II displays the conserved YxG motif, motif III the YLF triplet. Folding of the histone methyltransferases in knot-like structure brings together the catalytic residues NH and Y (shaded in yellow), embedded in the conserved signature motifs IV and VI (in bold). The F residue of the FxY motif (shaded in blue) is responsible for methylation specificity of SU(VAR)3–9 [114]. If changed to a Y, H3K9me3 modification is converted to H3K9me2. Mm: *Mus musculus*, Dm: *Drosophila melanogaster*, Sp: *Schizosaccharomyces pombe*, Cn: *Cryptococcus neoformans*, Fg: *Fusarium graminearum*, Ef: *Epichloë festucae*, Nc: *Neurospora crassa*, Pa: *Podospora anserina*. **D** Alignment of the pre/post-SET domains within SU(VAR)3–9 homologs for a set of selected species. Deleted parts of proteins are indicated by // (shaded in gray). Conserved pre-SET motif Zn3Cys9 (shaded in yellow) and post-SET CxCx4C (shaded in blue) [114]. Mm: *Mus musculus*, Dm: *Drosophila melanogaster*, Sp: *Schizosaccharomyces pombe*, Fg: *Fusarium graminearum*, Ef: *Epichloë festucae*, Nc: *Neurospora crassa*, Pa: *Podospora anserina*. Accession numbers for proteins used in alignments are listed in Additional file 24: Table S6.

**Additional file 2: Figure S2.** Expression kinetics *PaKmt1* in wild-type strain. Upper panel: RT-PCR on RNA extraction from wild-type vegetative growing mycelium (1 day and 4 days), perithecia (2 days and 4 days after the fertilization), and input genomic DNA. *PaKmt1* CDS was predicted to be made of two exons separated by a 62 bp intron (positions 49–110). However, two amplicons of distinct sizes are obtained, corresponding to both spliced (1108 bp) and unspliced (1170 bp) *PaKmt1* mRNAs. MW: molecular weight. Lower panel: schematic representation of the *PaKmt1* locus (DNA). mRNA1 corresponds to the spliced form of the transcripts while mRNA2 (1108 bp) corresponds to the unspliced form (1170 bp). Translation of the unspliced form would lead to a premature termination and thus to a truncated protein. Primers used for the reverse-transcription polymerase chain reaction (RT-PCR) are drawn as arrows above and below the *PaKmt1* CDS.

**Additional file 3: Figure S3. A** Heatmap of Spearman’s correlation coefficient comparison: clustering analysis of histone marks in wild-type background (WT). 2e and 3d are the two WT strains used for this study. They are issued from two spores from the same WT cross. Mock = IP performed with GFP antibody in absence of GFP tag in *P. anserina*’s genome (see “Methods”). Raw data are given in Additional file 25: Table S7. **B** H3K27me3, H3K4me3 and H3K9me3 proportion on *P. anserina* chromosomes in the WT, Δ*PaKmt1*, Δ*PaKmt6* and Δ*PaHP1* mutant strains. Plot showing the percentage of each chromosome covered with H3K4me3 (green), H3K9me3 (red) and H3K27me3 (blue). The coverage is the sum of all MACS2-predicted peak sizes. **C** Normalized ChIP-seq data representation for all marks on the seven *P. anserina* chromosomes for all conditions. ChIP-seq patterns display histone modification coverage and MACS2 detected peaks. **D** Domainogram representations for all marks on the seven *P. anserina* chromosomes for all conditions. Domainograms show significance of enrichment of H3K4me3, H3K9me3, H3K27me3 marks in windows of varying size. Color-coding of *p*-value is indicated (top).

**Additional file 4: Figure S4.** Combined epigenetic landscapes in wild-type and heterochromatin mutant strains of *P. anserina*. Panorama of genome-wide peak localization for each genotype, wild-type, Δ*PaKmt1*, Δ*PaKmt6* and Δ*PaHP1* strains. Telomeres sequences were arbitrarily defined as the segment going from the end of each arm of the chromosomes to the first annotated gene (with the exception of the rDNA cluster localized on chromosome 3) and centromeres are indicated. Mat region = Non-recombining region containing the mating-type locus as defined in [93]. A segment overlapping portions of chromosomes 3 and 4 is expanded to show a zoom of the combined epigenetic landscapes.

**Additional file 5: Figure S5.** H3K4me3, H3K27me3 and H3K9me3 modifications of *P. anserina* TEs in the WT, Δ*PaKmt1*, Δ*PaKmt6* and Δ*PaHP1* mutant strains. A. Histone marks on *P. anserina* TE families. Top panel: Plots of normalized ChIP signal: H3K4me3 (green), H3K9me3 (red) and H3K27me3 (dark blue) signals in the wild-type strain for five TE families, i.e., Copia, Gypsy, MITE, Tc1 Mariner, solo LTR and unclassified TEs [51] (Additional file 21: Table S3). Because MITE TEs are shorter than the other categories (< 500 bp in length), the genomic window was narrowed on the graph. Aligned sequences correspond to TE bodies ± 0.5 kbp surrounding region (see Additional file 21: Table S3 for TE’s numbers). Bottom panel: K-means built clusters representing the association versus non-association of the indicated histone modifications within a specific TE family. Histone modification levels in the heatmaps were calculated for non-overlapping 10-bp windows within the specific genomic regions and sorted by average value of each row. **B** Number of TEs, classified by family, marked with H3K4me3, H3K27me3 or H3K9me3, according to genetic backgrounds (wild-type strain, Δ*PaKmt1*, Δ*PaKmt6* and Δ*PaHP1* mutant strains). **C** Violin plots of expression of TEs classified by family. Gene expression was inferred from the TPM (Transcripts Per Kilobase Million) values calculated in [49]. Gene expression of non-repeated CDS (i.e., “genes”) was added for comparison.

**Additional file 6: Figure S6.** Snapshots of a set of TEs representing all the annotated TE families in *P. anserina*’s genome. ChIP-seq signals were normalized as described in “Methods” and visualized using the Integrated Genomics Viewer (IGV) [115]. H3K4me3 (green), H3K9me3 (red) and H3K27me3 (blue).

**Additional file 7: Figure S7.** Antibody specificity analysis. Dot blot results using H3K9me3 and H3K27me3 peptides (left) at different concentration (top). Membranes were inoculated with the corresponding antibody (right). Signal intensity comparison shows a cross-reactivity between anti-H3K9me3 antibody and H3K27me3 evaluated at 3% compared to the immunogen reaction.

**Additional file 8: Figure S8.** Molecular characterization of knockout mutants by Southern blot hybridization. Schematic representations of the endogenous and disrupted loci are given (Left). Replacement by homologous recombination of the wild-type *PaKmt1* allele by the disrupted Δ*PaKmt1* allele results in the substitution of 2.4 kbp and 5.7 kbp *Eco*RV fragments by a unique 11 kbp PstI fragment as revealed by hybridization of the 5′ and 3′ digoxygenin-labeled probes (dashed rectangles *PaKmt1* locus). Replacement by homologous recombination of the wild-type *PaKmt6* allele by the disrupted Δ*PaKmt6* allele results in the substitution of a unique 6.5 kbp KpnI fragment by two 1.8 and 2.4 kbp KpnI fragments as revealed by hybridization of the 5′ and 3′ digoxygenin-labeled probes (dashed rectangles *PaKmt6* locus). A second verification has been made with the HindIII enzymes and the same probes shows the substitution of two 1.9 kbp and 4.6 kbp HindIII fragments by a 3.7 kbp HindIII fragment as revealed by hybridization of the 5′ and 3′ digoxygenin-labeled probes.

**Additional file 9: Figure S9.** Localization of histone marks on specific genomic regions in the Δ*PaKmt1* and Δ*PaKmt6* mutant strains. Top panel: Plots of normalized ChIP-seq signal. Bottom panel: Heatmaps divided in K-means built clusters representing the association versus non-association of the indicated histone modifications with the specific genomic regions. Coding sequences or CDS were aligned by their two ends (indicated by START and STOP) ± 1 kbp of surrounding sequence (*N* = 10,839; Additional file 20: Table S2); repeats were defined as TE bodies, duplications and the rDNA array ± 0.2 kbp surrounding regions (*N* = 1680; Additional file 21: Table S3). Histone modification levels in the heatmaps were calculated for non-overlapping 10 bp windows within the specific genomic regions and sorted by average value of each row.

**Additional file 10: Figure S10.** Relative expression of selected genes in the Δ*PaKmt1* mutants. Caption as in Fig. 5a. The error bars represent the 95% confidence interval. No significant fold change was detected, except for *Pa_1_6263* and *Pa_6_7370*, which both lost the H3K27me3 mark and were up-regulated and down-regulated, respectively. *Pa_1_6263*: expression ratio = 1.855, *p*-value = 0.002; *Pa_4_1170*: expression ratio = 1.535, *p*-value = 0.094; *Pa_1_16300*: expression ratio = 1.393, *p*-value = 0.092; *Pa_6_7270*: expression ratio = 1.397, *p*-value = 0.035; *Pa_6_7370*: expression ratio = 0.696, *p*-value = 0.006; *Pa_5_10*: expression ratio = 0.759, *p*-value = 0.017; Pa_1_1880: expression ratio = 1.422, *p*-value = 0.034; Pa_7_9210: expression ratio = 1.082, *p*-value = 0.252; TC1mlr represent quantification of the cDNAs from the members of the Tc1_mariner-like_rainette family: expression ratio = 1.127, *p*-value = 0.339; TC1mlp represent quantification of the cDNAs from the members of the Tc1_mariner-like_pelobates family: expression ratio = 0.851, *p*-value = 0.308. Copia_Ty1_nephelobates transcripts could not be quantified as NRT-qPCR controls were too close to RT-qPCR (see Additional file 21: Table S3 for details of analysis).

**Additional file 11: Figure S11.** Growth features of the Δ*PaKmt1* strain. **A** Experimental procedure to test ability to resume growth of the Δ*PaKmt1* strain. To set up this restart test, mycelium implants issued from germination thalli were inoculated onto fresh M2 medium and incubated at 27 °C for 8 days (step 1). Two independent plugs from each location (purple cross), 8-day stationary phase (plug#3); pink cross, 4-day stationary phase (plug#2); and green cross, growing phase (plug#1) were then transferred to fresh M2 solid medium and incubated for 3 days at 27 °C (step 2). The growing phase of each thallus originating from step 2 (green margins) were transplanted again to fresh M2 solid medium and incubated at 27 °C for 3 days (step 3). Growth restart from stationary phase was impaired for Δ*PaKmt1* mutants, which resulted in smaller and thinner colonies than the wild-type ones (white arrows), whereas continuous growth (plug#1) was not altered. Complemented Δ*PaKmt1-PaKmt1*^+^ strains behaved as wild-type strains. As control experiments (step 3), we then transferred mycelia from growing margins (marked in green, step 2) of thalli deriving from Plug#1, Plug#2 and Plug#3. In this case, Δ*PaKmt1* mutants did not show any delay to resume growth (orange arrows), confirming that this defect was not permanent but rather linked to the disability of the Δ*PaKmt1* strains to resume growth properly. **B** Crippled growth test for Δ*PaKmt1* strain. Crippled growth (CG) process can be shown using a ‘band test’. Strains were incubated on M2 medium for 7 days at 27 °C. Two 1-mm-wide slices of agar were then inoculated onto fresh M2 media with or without yeast extract (YE) in the following method for 3 days. Picture shows actively growing apical hyphae (right) and resting stationary phase hyphae (left). The surface side of the slice on plate has been orientated at the top. CG is a degenerative process caused by C element production in the stationary phase. When inoculated on yeast extract medium, it displays slow growth, alteration of pigmentation, inability to differentiate aerial hyphae, and female sterility*.* The Δ*PaMpk1* mutant strain is impaired for CG development while the *PDC2208* strain is supposed to display CG on M2 medium without yeast extract [116]. The single Δ*PaKmt1* mutants are not impaired for CG.

**Additional file 12: Figure S12.** Longevity tests for Δ*PaKmt1* and Δ*PaKmt6* strains. Graphs show the maximal growth length on M2 medium at 27 °C in race tubes for wild-type (WT), Δ*PaKmt1* and Δ*PaKmt6* mutant strains for both mating types. Data correspond to the mean of three technical replicates from five biological samples.

**Additional file 13: Figure S13.** Role of *PaKmt6* during sexual development. **A** Heterozygote oriented crosses between wild-type strains (WT) and Δ*PaKmt6* mutants. Fertilization of wild-type ascogonia with Δ*PaKmt6* male gametes results in normal fruiting body development displaying a single neck (black arrow), while fertilization of Δ*PaKmt6* ascogonia with wild-type spermatia results in fewer crippled Δ*PaKmt6*-like fruiting bodies (two necks are indicated by two black arrows). These features indicate that *PaKmt6* is a maternal gene. **B** Mosaic analyses using the Δ*mat* mutant strain. Wild-type or Δ*PaKmt6 mat*+ and *mat*− strains were mixed with or without Δ*mat* mycelium and inoculated onto fresh M2 medium. After a week of incubation on M2 medium, Δ*PaKmt6* dikaryon displayed crippled and mis-orientated perithecia, resulting in scattered and reduced ascospore production. The tricaryon Δ*PaKmt6 mat*+ /Δ*PaKmt6 mat−*/Δ*mat* showed nearly wild-type restoration of mycelium growth, as well as perithecia and ascospore production. These features confirm that *PaKmt6* is a gene expressed in the maternal tissues of the perithecium and not in its zygotic tissues (centrum).

**Additional file 14: Figure S14.** Structure and evolutionary relationships of HP1 orthologs. **A** Domain structure comparison of heterochromatin protein 1 homologs. Size in amino acids (aa) is given (right). PaHP1 displays the evolutionary conserved and topologically connected chromodomain (turquoise) and chromoshadowdomain (dark green), along with three disordered elements: the N-terminal extension (NTE), the Hinge region (HR) and the C-terminal extension (CTE). Chromodomain recognizes H3K9me2/3 histone modification, while chromoshadowdomain is a protein–protein interaction domain. **B** Alignment of the chromodomains of HP1 homologs for a set of selected species. The aromatic cage residues (three aromatic amino acids shaded in yellow, i.e., Y and WxxY) form the binding cavity for H3K9me. Residues involve in the H3 peptide recognition surface are highlighted in blue. Residues of chromo-shadow domain involved in dimerization are shown in green, those involved in folding are shown in lavender [63].

**Additional file 15: Figure S15. A** Tree showing evolution of some HP1 orthologs from fungi, plant and metazoans. The *P. anserina* heterochromatin protein-1 homolog is marked with a rectangle. Bootstraps are given. Filamentous fungi: *Podospora anserina* (Pa), *Neurospora crassa* (Nc), *Fusarium graminearum* (Fg), *Trichoderma reesei* (Tr), *Epichloë festucae* (Ef) *Botrytis cinerea* (Bc), *Magnaporthe oryzae* (Mo), *Zymoseptoria tritici* (Zt), *Leptosphaeria maculans* (Lm), *Aspergillus nidulans* (An), *Aspergillus fumigatus* (Af), *Penicillium oxalicum* (Po), *Ascobolus immersus* (Ai), *Puccinia graminis* (Pg), *Pneumocystis jirovecii* (Pj), *Conidiobolus coronatus* (Cc), *Mucor circinelloides* (Mc); yeasts: *Schizosaccharomyces pombe* (Sp) and *Cryptococcus neoformans* (Cn); the worm *Caenorhabditis elegans* (Ce), the fruit-fly *Drosophila melanogaster* (Dm), the mouse *Mus musculus* (Mm) and the model plant *Arabidopsis thaliana* (At). Accession numbers for proteins used in alignments are listed in Additional file 24: Table S6. **B** Molecular characterization of knockout Δ*PaHP1* mutants by Southern blot hybridization. Replacement by homologous recombination of the wild-type *PaHP1* allele by the disrupted Δ*PaHP1* allele results in the substitution of a unique 3.4 kb EcoRI/EcoRV fragment by two 1.4 and 2.5 kb EcoRI/EcoRV fragments as revealed by hybridization of the 5′UTR and 3′UTR digoxygenin-labeled probes (dashed rectangles *PaHP1* locus). **C** Vegetative growth of Δ*PaHP1 single* mutants and Δ*PaKmt1*Δ*PaHP1* double mutants compared to wild-type strains. Δ*PaKmt1* and Δ*PaHP1* single mutants are impaired in aerial mycelium production. This defect was more pronounced for the single Δ*PaHP1* mutants*.* Δ*PaKmt1*Δ*PaHP1* double mutants show Δ*PaKmt1* morphologic phenotype. The strains have been grown on M2 medium 3 days.

**Additional file 16: Figure S16.** Localization of histone marks on specific genomic regions in the Δ*PaPH1* mutant strains. Top panel: Plots of normalized ChIP-seq signal. Bottom panel: heatmaps divided in K-means built clusters representing the association versus non-association of the indicated histone modifications with the specific genomic regions. Coding sequences or CDS were aligned by their two ends (indicated by START and STOP) ± 1 kbp of surrounding sequence (*N* = 10,839; Additional file 20: Table S2); repeats were defined as TE bodies, duplications and the rDNA array ± 0.2 kbp surrounding regions (*N* = 1680; Additional file 20: Table S3). Histone modification levels in the heatmaps were calculated for non-overlapping 10 bp windows within the specific genomic regions and sorted by average value of each row.

**Additional file 17: Figure S17.** **A** Relative expression of selected genes in the Δ*PaHP1* strain. Caption as in Fig. 5a. The error bars represent the 95% confidence interval. No significant fold change was assayed, except for *Pa_1_6263* and *Pa_5_10*, which both lost the H3K27me3 mark and were up-regulated and down-regulated, respectively. *Pa_1_6263*: expression ratio = 2.463, *p*-value = 0.004; *Pa_4_1170*: expression ratio = 1.911, *p*-value = 0.032; *Pa_1_16300*: expression ratio = 0.931, *p*-value = 0.693; *Pa_6_7270*: expression ratio = 1.527, *p*-value = 0.004; *Pa_6_7370*: expression ratio = 0.766, *p*-value = 0.009; *Pa_5_10*: expression ratio = 0.490, *p*-value = 0.006; Pa_1_1880: expression ratio = 1.313, *p*-value = 0.013; Pa_7_9210: expression ratio = 0.951, *p*-value = 0.547; TC1mlr represents quantification of the cDNAs from the members of the Tc1_mariner-like_rainette family: expression ratio = 1.264, *p*-value = 0.008. Tc1_mariner-like_pelobates and copia_Ty1_nephelobates transcripts could not be quantified as NRT-qPCR controls were too close to RT-qPCR (see Additional file 21: Table S3 for details of analysis). **B** Vegetative growth kinetics of Δ*PaHP1* mutants and Δ*PaKmt1* mutants compared to wild-type strains. See “Methods” section for details. **C** Experimental procedure to test growth resuming capabilities of Δ*PaHP1* and double Δ*PaHP1*Δ*PaKmt1* mutants. For description of experimental settings see Additional file 8: Fig. S8A. Growth restart from stationary phase (plug#2 and plug#3) was impaired for Δ*PaHP1* and double Δ*PaHP1*Δ*PaKmt1* mutants, which resulted in smaller and thinner colonies than the wild-type ones (white arrows), whereas continuous growth (plug#1) was not altered. Complemented Δ*PaHP1-PaHP1*^+^ strains behaved as wild-type strains. As control experiments (step 3), we transferred mycelia from growing margins (marked in green, step 2) of thalli derived from Plug#1, Plug#2 and Plug#3. In this case, neither Δ*PaKmt1* mutants nor Δ*PaHP1*Δ*PaKmt1* mutants showed any delay to resume growth (orange arrows). **D** Crippled growth test of Δ*PaHP1* single mutants and Δ*PaHP1*Δ*PaKmt1* double mutants. For description of experimental settings see Additional file 8: Fig. S8B. The single Δ*PaHP1* and the double Δ*PaKmt1*Δ*PaHP1* mutants are not impaired for CG.

**Additional file 18: Figure S18.** Heat map of Spearman’s correlation coefficient comparison. Clustering analysis of histone marks in wild-type background (WT) and in mutant backgrounds Δ*PaKmt1*, Δ*PaKmt6* and Δ*PaHP1*. Mock = IP performed with GFP antibody in the absence of any GFP tag in the *P. anserina* genome (see “Methods”). Raw data are given in Additional file 26: Table S8.

**Additional file 19: Table S1.** Main features of significantly enriched peaks. Peak numbers, sizes, and genome coverage of H3K4me3, H3K9me3 and H3K27me3 modifications, in wild-type background (WT) and in mutant backgrounds Δ*PaKmt1*, Δ*PaKmt6* and Δ*PaHP1*.

**Additional file 20: Table S2.** Distribution of H3K4me3, H3K9me3 and H3K27me3 on the complete set of *P. anserina* genes in wild-type background (WT) and in Δ*PaKmt1*, Δ*PaKmt6* and Δ*PaHP1* mutants. List of genes where H3K4me3 and H3K27me3 can be found overlapping in the wild-type background. Distribution of H3K4me3, H3K9me3 and H3K27me3 on a subset of *P. anserina* secondary metabolite gene clusters in wild-type background (WT) and in *PaKmt1*, Δ*PaKmt6* and Δ*PaHP1* mutants. ChIP_peaks, 0 means no peaks detected by MACS2, 1 means at least one peak detected by MACS2. ChIP_coverage: mapped reads after normalization (see “ChIP-seq sequencing data analysis and visualization” paragraph in “Methods” for details). All_BRICKS: H3K4me3, H3K9me3 or H3K27me3 domainograms were simplified as described in [50] to a set of discrete domains of enrichment called Blocks of Regulators In Chromosomal Kontext (BRICKs). BRICKS can be used to define domains with significant clustering of ChIP-seq signal and their boundaries, while domainograms provide an overview.

**Additional file 21: Table S3.** Distribution of H3K4me3, H3K9me3 and H3K27me3 on the complete set of *P. anserina* transposable elements in wild-type background (WT) and in Δ*PaKmt1*, Δ*PaKmt6* and Δ*PaHP1* mutants. ChIP_peaks, 0 means no peaks detected by MACS2, 1 means at least one peak detected by MACS2. ChIP_coverage: mapped reads after normalization (see “ChIP-seq sequencing data analysis and visualization” paragraph in “Methods” for details).

**Additional file 22: Table S4.** Cq for RT-qPCR experiments and geNorm analysis of candidate normalization genes.

**Additional file 23: Table S5.** Primers used for PCR experiments. **A** Primers used for RT-PCR and allele construction experiments. **B** Primers used for RT-qPCR experiments.

**Additional file 24: Table S6.** Accession numbers for proteins used in alignments to build the phylogenic trees.

**Additional file 25: Table S7.** Heatmap of Spearman correlation coefficient comparison between ChIP-seq samples. Samples are labeled as follow: Genotype_strain_marks. E.g., KMT6-11d_K9 = Δ*PaKmt6* strain, from spore number 11d (biological replicate), IP = H3K9me3. Spearman’s correlation coefficient confirms the reproducibility of the experiments since biological replicates cluster together with high correlation coefficients. Moreover, H3K9me3 and H3K27me3 IP share a same cluster that negatively correlates with the H3K4me3 cluster. Both display no correlation with non-IP input and negative sample (Mock and H3K27me3 in the Δ*PaKmt6*), which means that IP results are significant and specific.

**Additional file 26: Table S8.** Raw data from the heatmap of Spearman’s correlation coefficient comparison in Additional file 18: Fig. S18.

## Data Availability

The datasets generated and/or analyzed during the current study are available in the NCBI Sequence Read Archive (SRA) (BioProject: PRJNA574032, https://www.ncbi.nlm.nih.gov/bioproject/?term=PRJNA574032).
