# Supplementary figures and images for: Loss of EZH2-like or SU(VAR)3–9-like proteins causes simultaneous perturbations in H3K27 and H3K9 tri-methylation and associated developmental defects in the fungus Podospora anserina
Source: Epigenetics Chromatin. 2021 May 7;14:22. doi: 10.1186/s13072-021-00395-7 (PMC8105982; doi:10.1186/s13072-021-00395-7)

## Slide 1
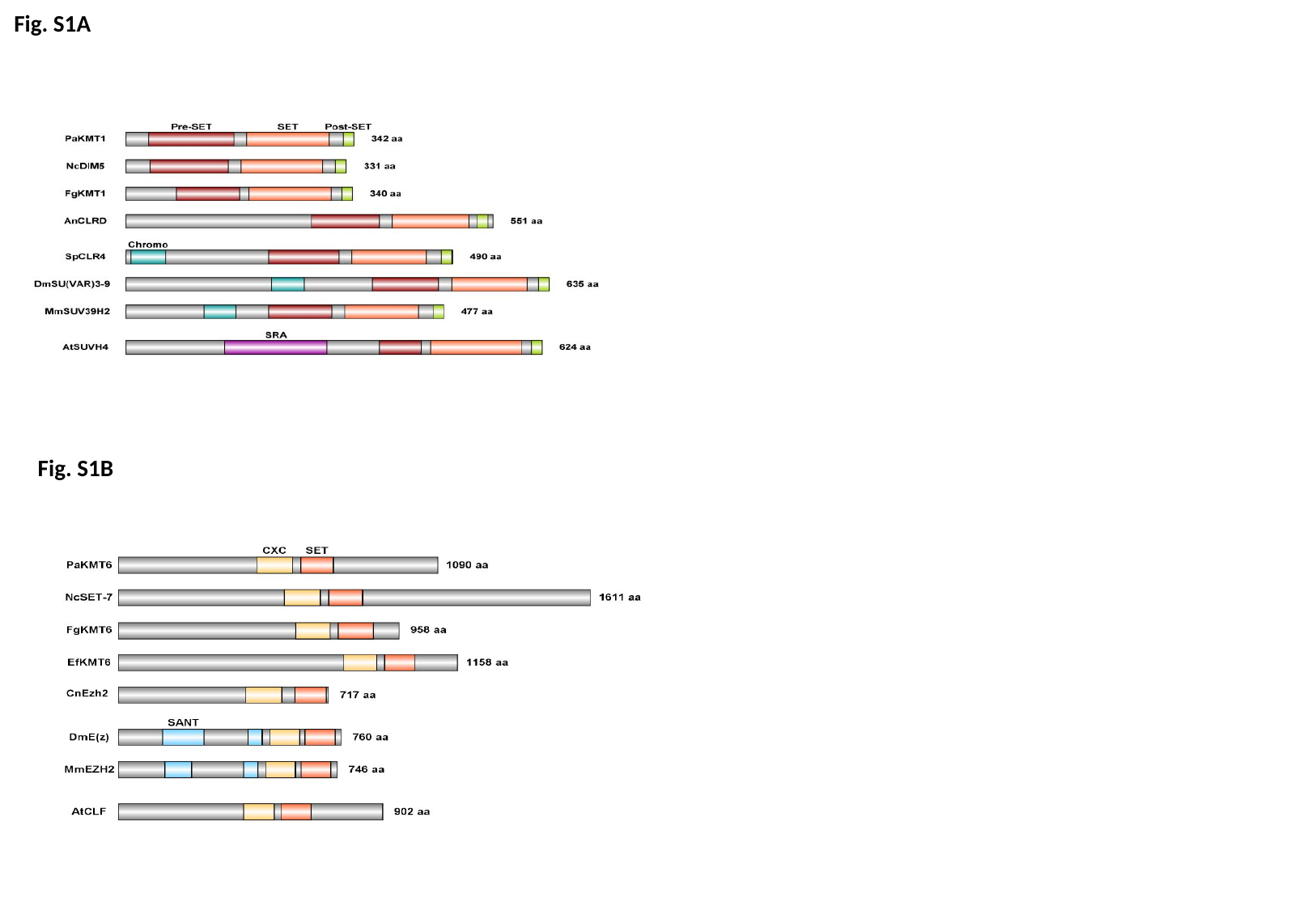

Fig. S1A
Fig. S1B

## Slide 2
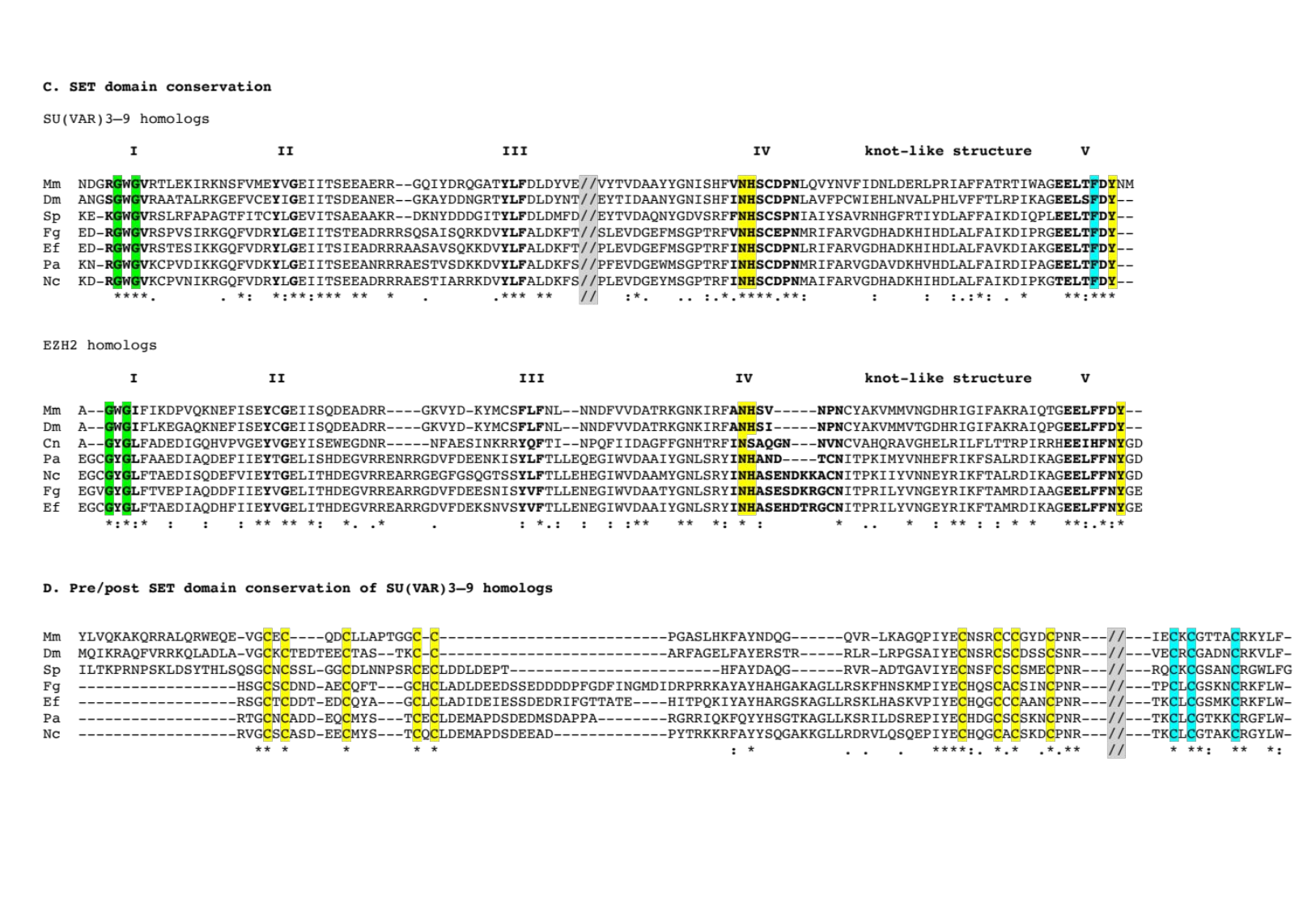

Supplement: Supplementary file 1 — Additional file 1: Figure S1. Conserved structure and phylogenetic analysis of histone methyltransferases involved in heterochromatin assembly. Domain structure comparison of histone methyltransferase Kmt1 (A) and Kmt6 (B). Sizes in amino acid (aa) are given (right). Pre-SET (red, IPR007728), SET (orange, IPR001214) and Post-SET (green, IPR003616) conserved domains are required for H3K9 methyltransferase activity of Kmt1 homolog proteins. SRA (SET and RING finger-associated, purple, IPR003105) and SANT (blue, IPR001005) are protein–protein interaction domains. CXC (yellow, IPR026489) is a cysteine-rich conserved domain located in the H3K27 methyltransferase catalytic domain of Kmt6 homologs. Filamentous fungi: Podospora anserina (Pa), Neurospora crassa (Nc), Fusarium graminearum (Fg), Epichloë festucae (Ef), Aspergillus nidulans (An); yeasts: Schizosaccharomyces pombe (Sp) and Cryptococcus neoformans (Cn); the worm Caenorhabditis elegans (Ce), the fruit-fly Drosophila melanogaster (Dm), the mouse Mus musculus (Mm) and the model plant Arabidopsis thaliana (At). Accession numbers for proteins used in alignments are listed in Additional file 24: Table S6. C Alignment of the SET domains within SU(VAR)3–9 (upper panel) and EZH2 (lower) homologs for a set of selected species. Deleted parts of proteins are indicated by // (shaded in gray). Conserved GxG motif (shaded in green) corresponds to the substrate AdoMet/SAM binding site (motif I). Motif II displays the conserved YxG motif, motif III the YLF triplet. Folding of the histone methyltransferases in knot-like structure brings together the catalytic residues NH and Y (shaded in yellow), embedded in the conserved signature motifs IV and VI (in bold). The F residue of the FxY motif (shaded in blue) is responsible for methylation specificity of SU(VAR)3–9 [114]. If changed to a Y, H3K9me3 modification is converted to H3K9me2. Mm: Mus musculus, Dm: Drosophila melanogaster, Sp: Schizosaccharomyces pombe, Cn: Cryptococcus neof [file 13072_2021_395_MOESM1_ESM.pptx]

## Slide 1
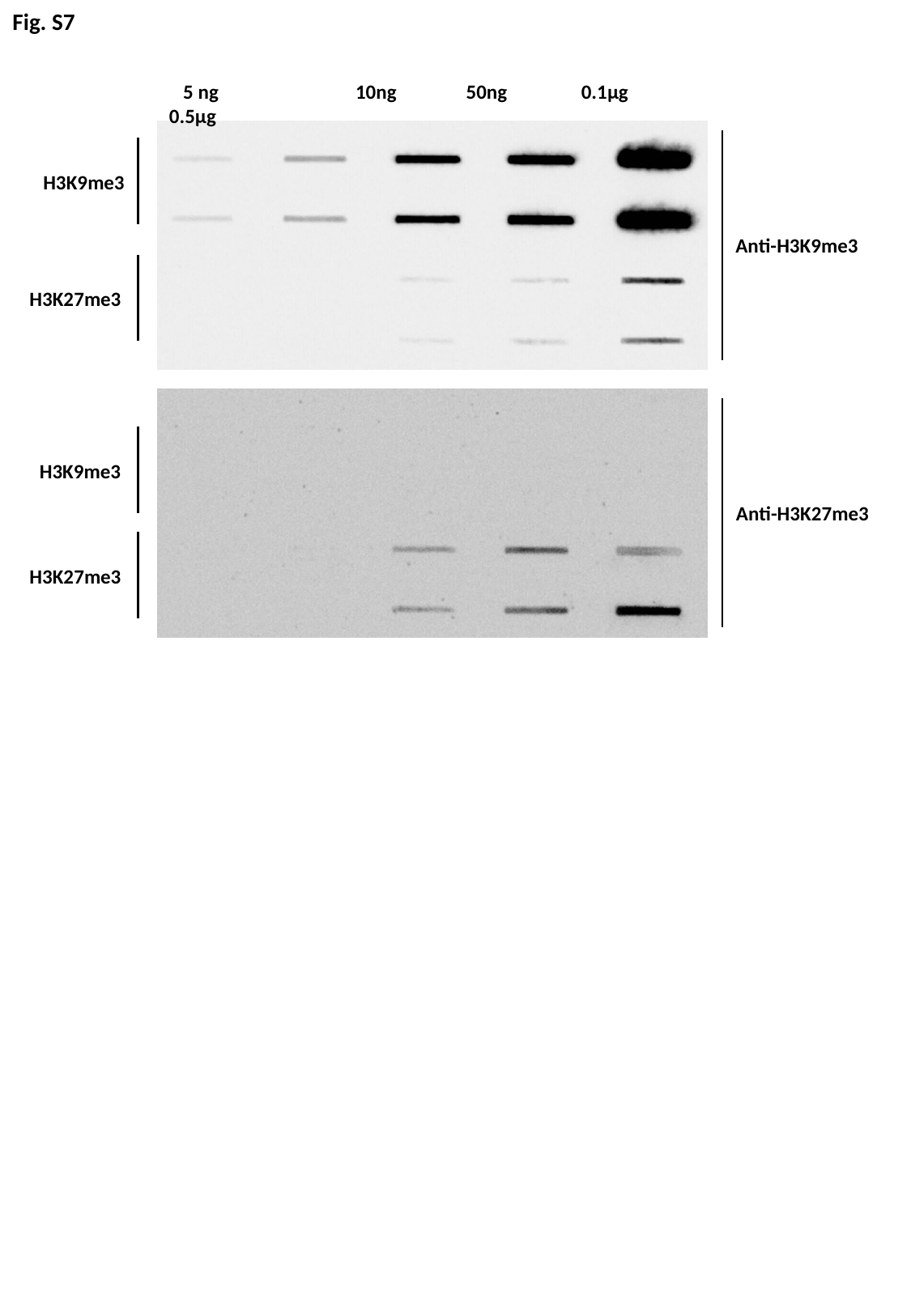

Fig. S7
 5 ng	 10ng 50ng 0.1µg 0.5µg
Anti-H3K9me3
H3K9me3
H3K27me3
Anti-H3K27me3
H3K9me3
H3K27me3

Supplement: Supplementary file 7 — Additional file 7: Figure S7. Antibody specificity analysis. Dot blot results using H3K9me3 and H3K27me3 peptides (left) at different concentration (top). Membranes were inoculated with the corresponding antibody (right). Signal intensity comparison shows a cross-reactivity between anti-H3K9me3 antibody and H3K27me3 evaluated at 3% compared to the immunogen reaction. [file 13072_2021_395_MOESM7_ESM.pptx]

## Slide 1
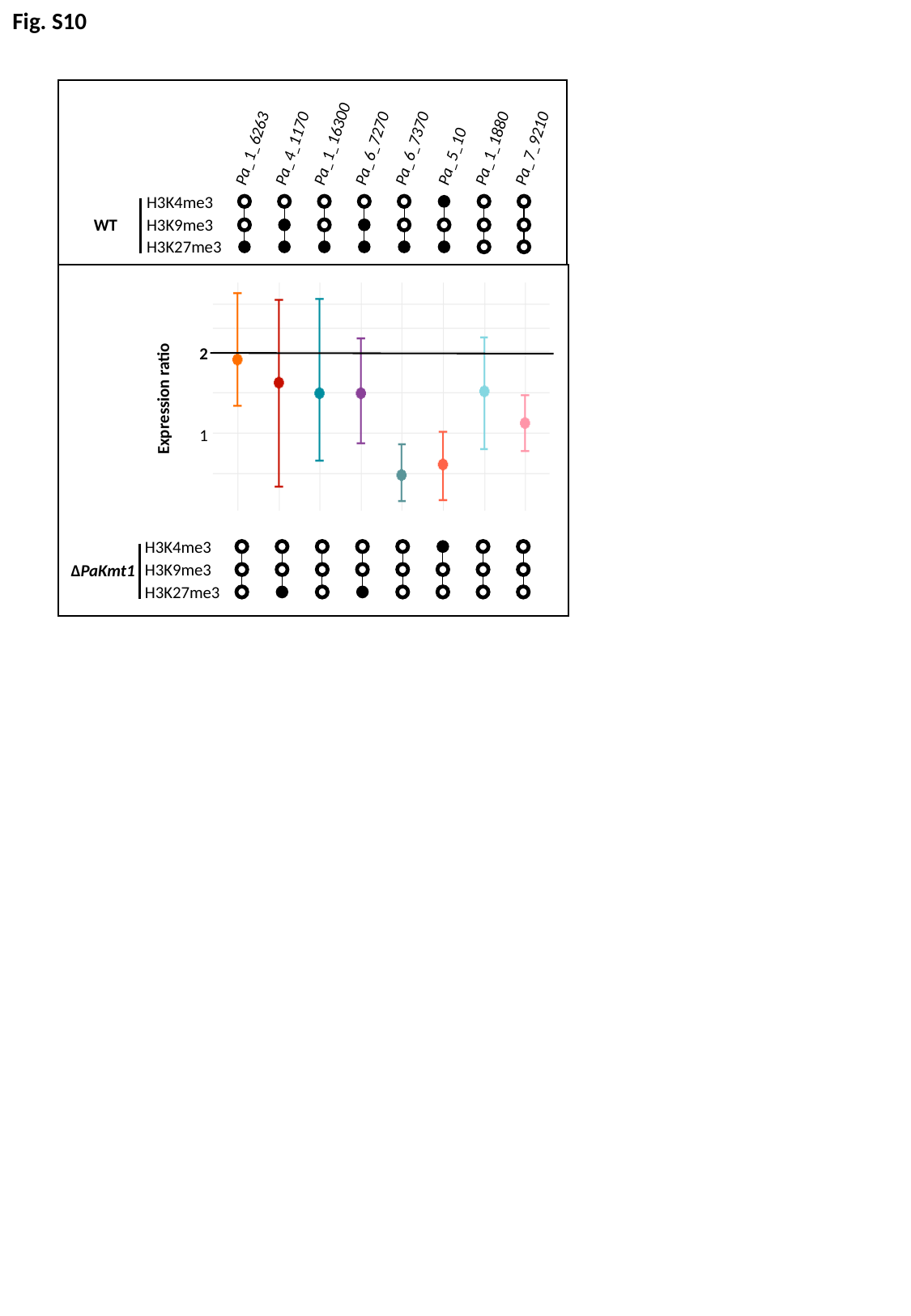

Fig. S10
Pa_1_16300
Pa_1_6263
Pa_4_1170
Pa_6_7270
Pa_6_7370
Pa_1_1880
Pa_7_9210
Pa_5_10
H3K4me3
H3K9me3
WT
H3K27me3
2
Expression ratio
1
H3K4me3
H3K9me3
∆PaKmt1
H3K27me3

Supplement: Supplementary file 10 — Additional file 10: Figure S10. Relative expression of selected genes in the ΔPaKmt1 mutants. Caption as in Fig. 5a. The error bars represent the 95% confidence interval. No significant fold change was detected, except for Pa_1_6263 and Pa_6_7370, which both lost the H3K27me3 mark and were up-regulated and down-regulated, respectively. Pa_1_6263: expression ratio = 1.855, p-value = 0.002; Pa_4_1170: expression ratio = 1.535, p-value = 0.094; Pa_1_16300: expression ratio = 1.393, p-value = 0.092; Pa_6_7270: expression ratio = 1.397, p-value = 0.035; Pa_6_7370: expression ratio = 0.696, p-value = 0.006; Pa_5_10: expression ratio = 0.759, p-value = 0.017; Pa_1_1880: expression ratio = 1.422, p-value = 0.034; Pa_7_9210: expression ratio = 1.082, p-value = 0.252; TC1mlr represent quantification of the cDNAs from the members of the Tc1_mariner-like_rainette family: expression ratio = 1.127, p-value = 0.339; TC1mlp represent quantification of the cDNAs from the members of the Tc1_mariner-like_pelobates family: expression ratio = 0.851, p-value = 0.308. Copia_Ty1_nephelobates transcripts could not be quantified as NRT-qPCR controls were too close to RT-qPCR (see Additional file 21: Table S3 for details of analysis). [file 13072_2021_395_MOESM10_ESM.pptx]

## Slide 1
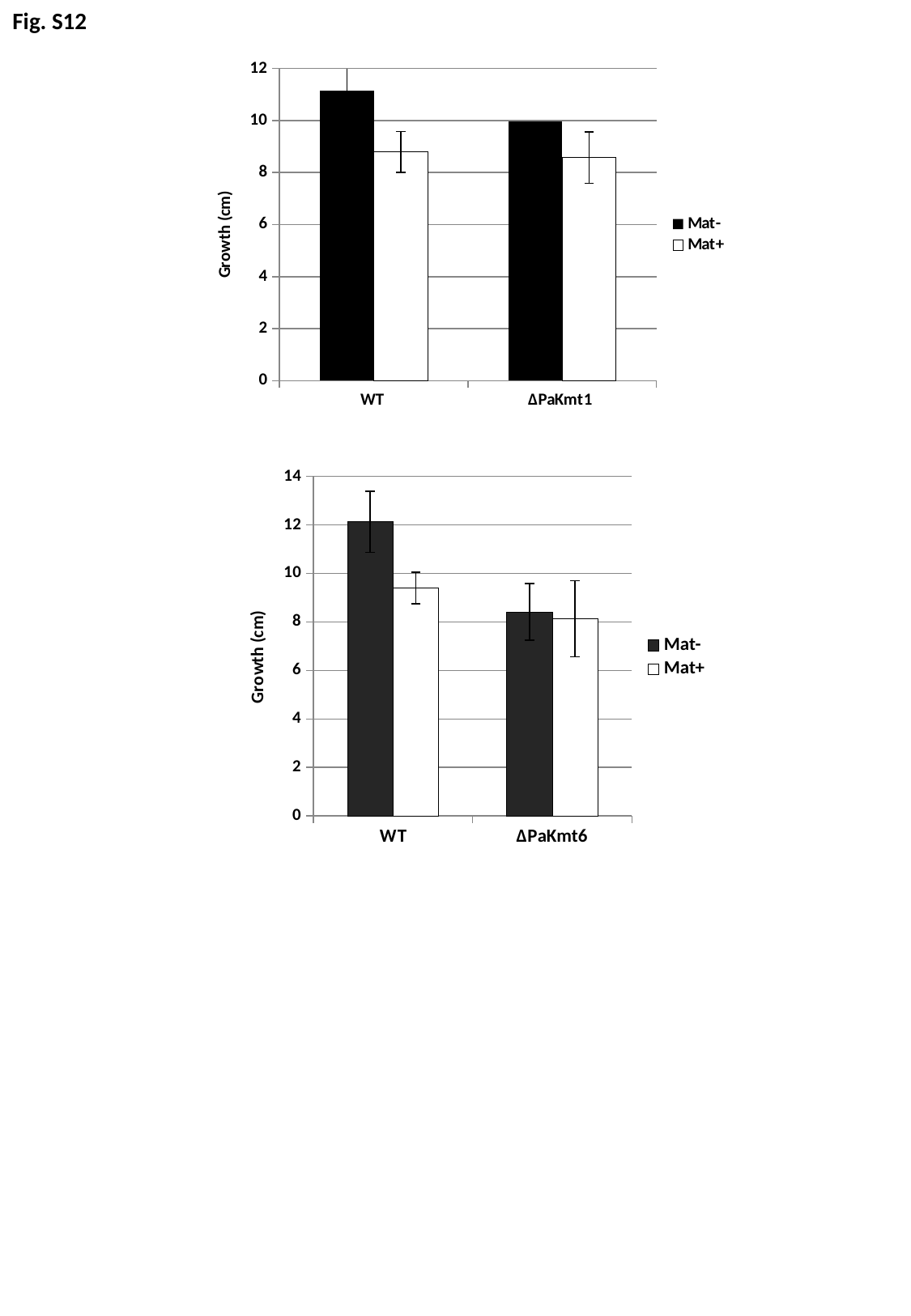

Fig. S12
### Chart
| Category | Mat- | Mat+ |
|---|---|---|
| WT | 11.14 | 8.79285714285712 |
| ΔPaKmt1 | 9.96666666666667 | 8.580000000000002 |
### Chart
| Category | Mat- | Mat+ |
|---|---|---|
| WT | 12.12333333333333 | 9.4 |
| ΔPaKmt6 | 8.413333333333332 | 8.133333333333333 |

Supplement: Supplementary file 12 — Additional file 12: Figure S12. Longevity tests for ΔPaKmt1 and ΔPaKmt6 strains. Graphs show the maximal growth length on M2 medium at 27 °C in race tubes for wild-type (WT), ΔPaKmt1 and ΔPaKmt6 mutant strains for both mating types. Data correspond to the mean of three technical replicates from five biological samples. [file 13072_2021_395_MOESM12_ESM.pptx]

## Slide 1
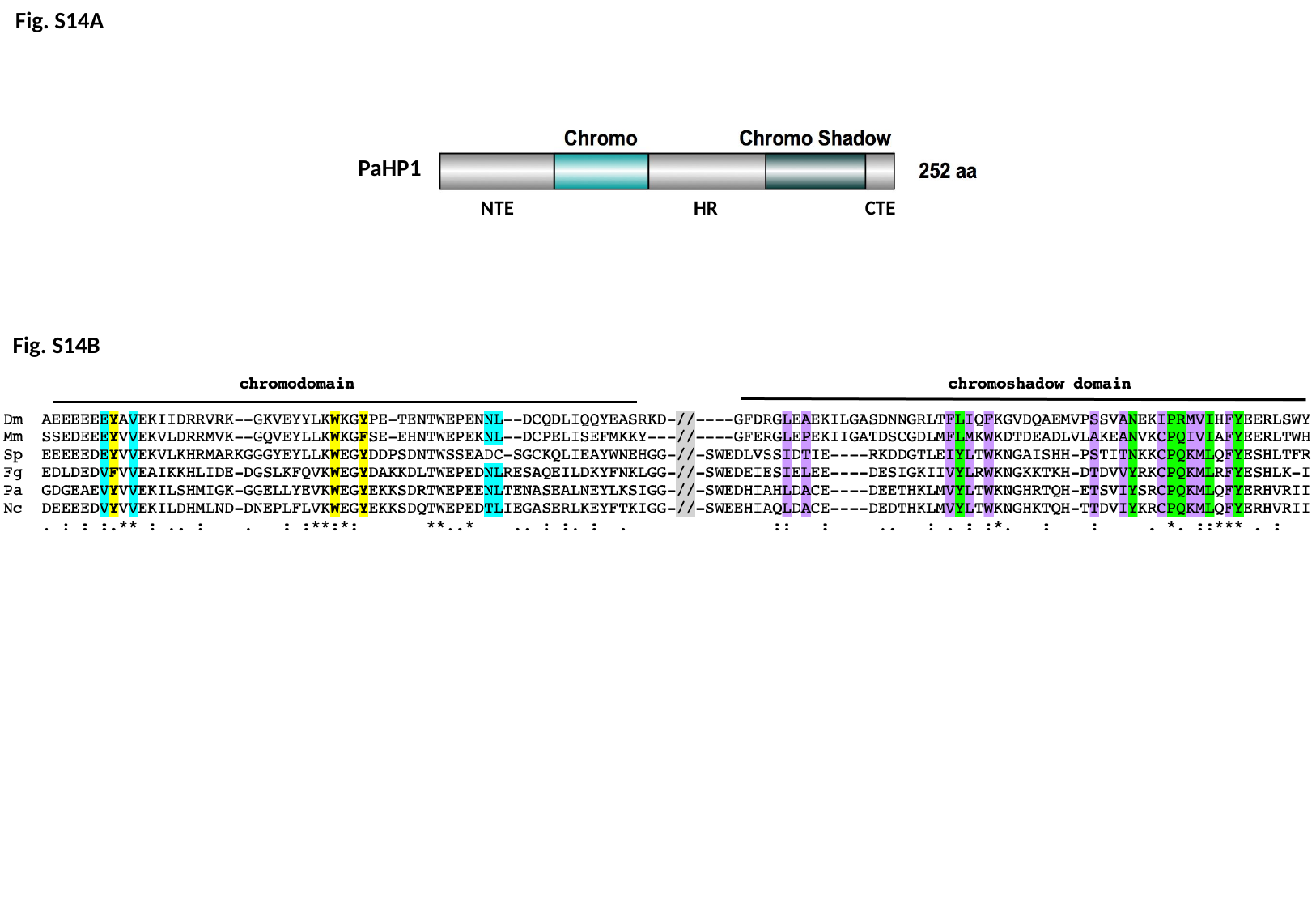

Fig. S14A
PaHP1
NTE
HR
CTE
Fig. S14B

Supplement: Supplementary file 14 — Additional file 14: Figure S14. Structure and evolutionary relationships of HP1 orthologs. A Domain structure comparison of heterochromatin protein 1 homologs. Size in amino acids (aa) is given (right). PaHP1 displays the evolutionary conserved and topologically connected chromodomain (turquoise) and chromoshadowdomain (dark green), along with three disordered elements: the N-terminal extension (NTE), the Hinge region (HR) and the C-terminal extension (CTE). Chromodomain recognizes H3K9me2/3 histone modification, while chromoshadowdomain is a protein–protein interaction domain. B Alignment of the chromodomains of HP1 homologs for a set of selected species. The aromatic cage residues (three aromatic amino acids shaded in yellow, i.e., Y and WxxY) form the binding cavity for H3K9me. Residues involve in the H3 peptide recognition surface are highlighted in blue. Residues of chromo-shadow domain involved in dimerization are shown in green, those involved in folding are shown in lavender [63]. [file 13072_2021_395_MOESM14_ESM.pptx]
