# Supplementary material for: Loss of EZH2-like or SU(VAR)3–9-like proteins causes simultaneous perturbations in H3K27 and H3K9 tri-methylation and associated developmental defects in the fungus Podospora anserina
Source: Epigenetics Chromatin. 2021 May 7;14:22. doi: 10.1186/s13072-021-00395-7 (PMC8105982; doi:10.1186/s13072-021-00395-7)
Supplement: Supplementary file 2 — Additional file 2: Figure S2. Expression kinetics PaKmt1 in wild-type strain. Upper panel: RT-PCR on RNA extraction from wild-type vegetative growing mycelium (1 day and 4 days), perithecia (2 days and 4 days after the fertilization), and input genomic DNA. PaKmt1 CDS was predicted to be made of two exons separated by a 62 bp intron (positions 49–110). However, two amplicons of distinct sizes are obtained, corresponding to both spliced (1108 bp) and unspliced (1170 bp) PaKmt1 mRNAs. MW: molecular weight. Lower panel: schematic representation of the PaKmt1 locus (DNA). mRNA1 corresponds to the spliced form of the transcripts while mRNA2 (1108 bp) corresponds to the unspliced form (1170 bp). Translation of the unspliced form would lead to a premature termination and thus to a truncated protein. Primers used for the reverse-transcription polymerase chain reaction (RT-PCR) are drawn as arrows above and below the PaKmt1 CDS. [file 13072_2021_395_MOESM2_ESM.pptx]

## Slide 1
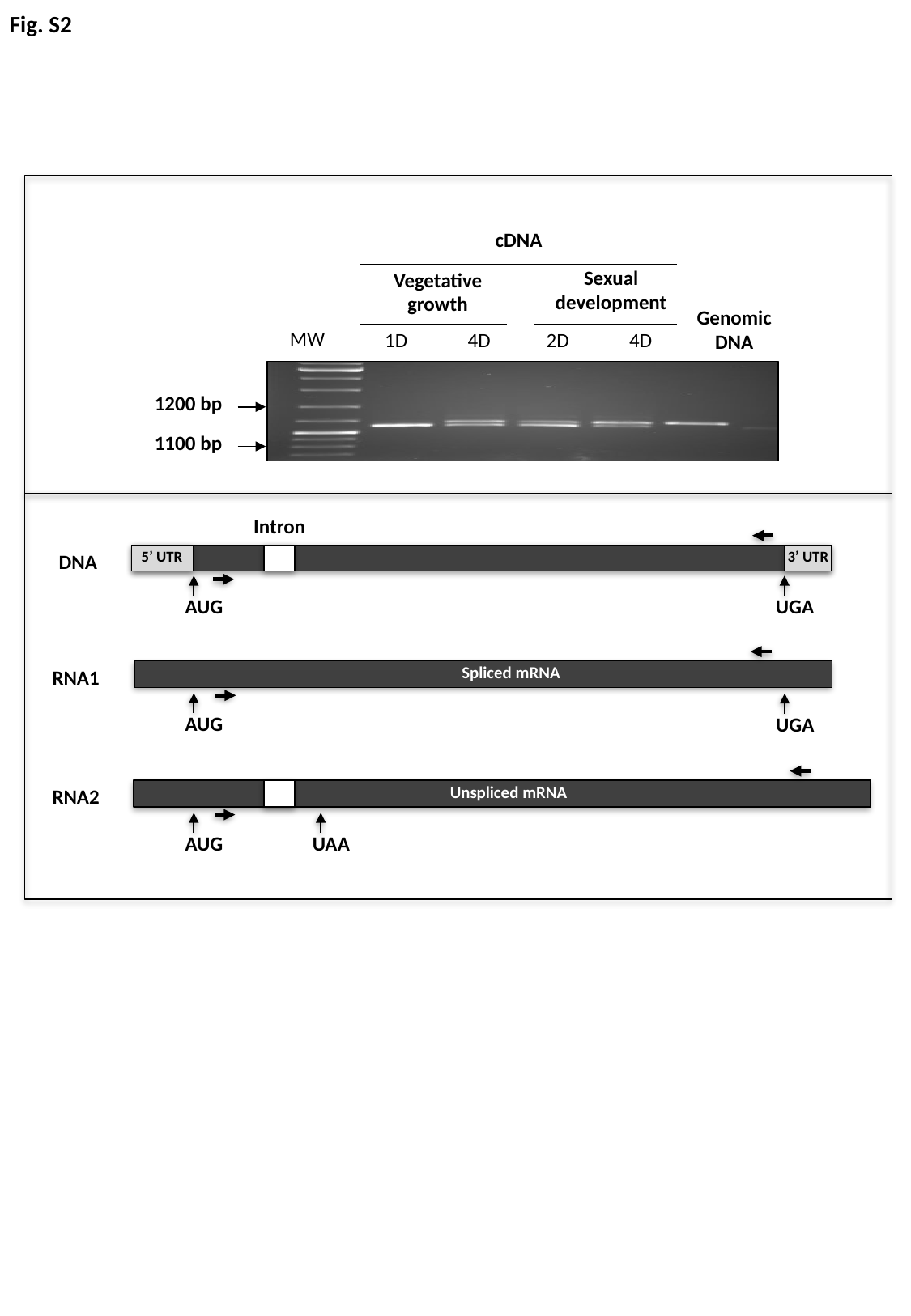

Fig. S2
cDNA
Sexual development
Vegetative growth
Genomic DNA
MW
 1D 4D 2D 4D
1200 bp
1100 bp
Intron
5’ UTR
3’ UTR
DNA
AUG
UGA
Spliced mRNA
RNA1
AUG
UGA
Unspliced mRNA
RNA2
UAA
AUG
