# Supplementary material for: Loss of EZH2-like or SU(VAR)3–9-like proteins causes simultaneous perturbations in H3K27 and H3K9 tri-methylation and associated developmental defects in the fungus Podospora anserina
Source: Epigenetics Chromatin. 2021 May 7;14:22. doi: 10.1186/s13072-021-00395-7 (PMC8105982; doi:10.1186/s13072-021-00395-7)
Supplement: Supplementary file 3 — Additional file 3: Figure S3. A Heatmap of Spearman’s correlation coefficient comparison: clustering analysis of histone marks in wild-type background (WT). 2e and 3d are the two WT strains used for this study. They are issued from two spores from the same WT cross. Mock = IP performed with GFP antibody in absence of GFP tag in P. anserina’s genome (see “Methods”). Raw data are given in Additional file 25: Table S7. B H3K27me3, H3K4me3 and H3K9me3 proportion on P. anserina chromosomes in the WT, ΔPaKmt1, ΔPaKmt6 and ΔPaHP1 mutant strains. Plot showing the percentage of each chromosome covered with H3K4me3 (green), H3K9me3 (red) and H3K27me3 (blue). The coverage is the sum of all MACS2-predicted peak sizes. C Normalized ChIP-seq data representation for all marks on the seven P. anserina chromosomes for all conditions. ChIP-seq patterns display histone modification coverage and MACS2 detected peaks. D Domainogram representations for all marks on the seven P. anserina chromosomes for all conditions. Domainograms show significance of enrichment of H3K4me3, H3K9me3, H3K27me3 marks in windows of varying size. Color-coding of p-value is indicated (top). [file 13072_2021_395_MOESM3_ESM.pptx]

## Slide 1
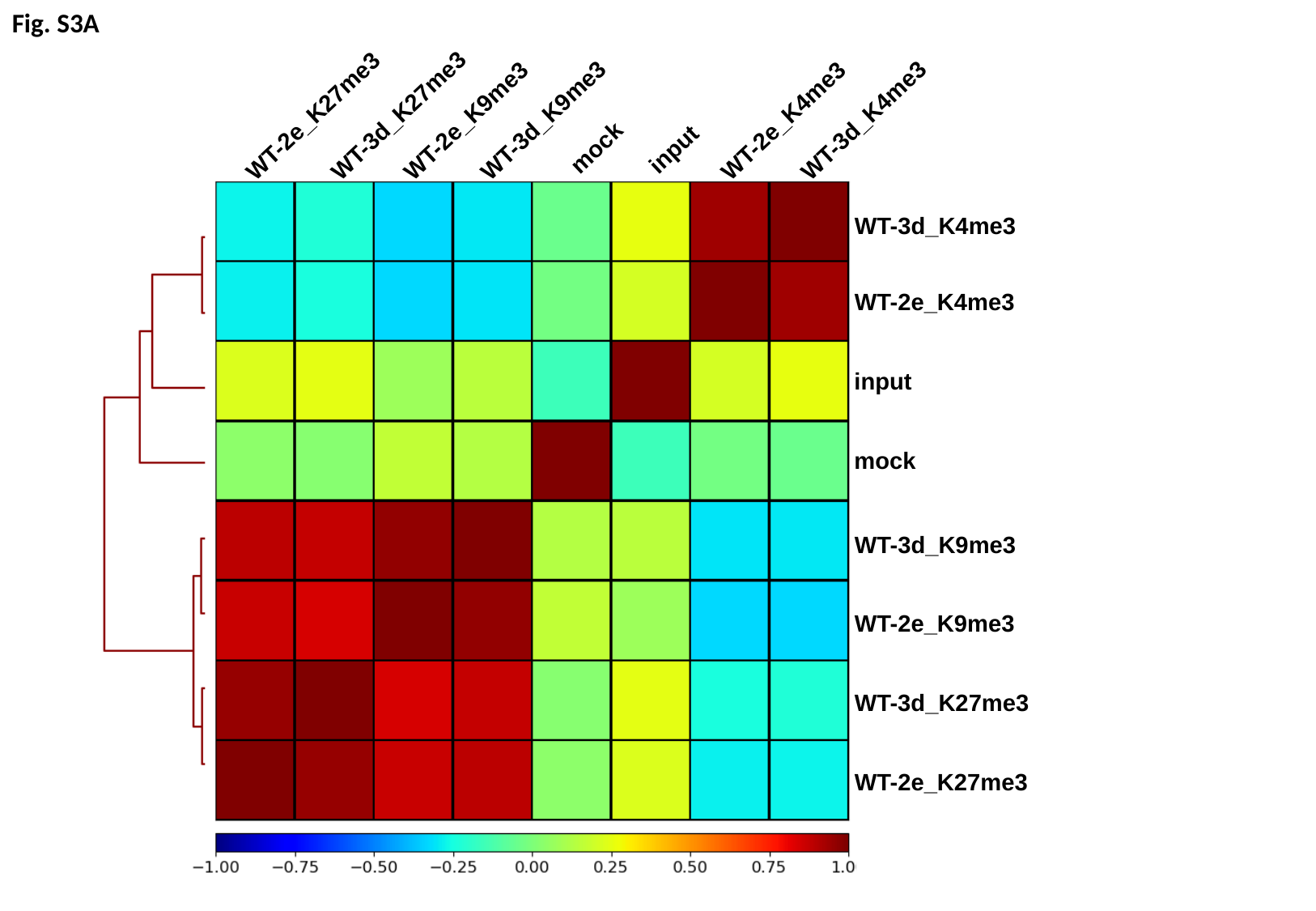

Fig. S3A
WT-3d_K27me3
WT-2e_K27me3
WT-3d_K9me3
WT-3d_K4me3
WT-2e_K9me3
WT-2e_K4me3
mock
input
WT-3d_K4me3
WT-2e_K4me3
input
mock
WT-3d_K9me3
WT-2e_K9me3
WT-3d_K27me3
WT-2e_K27me3

## Slide 2
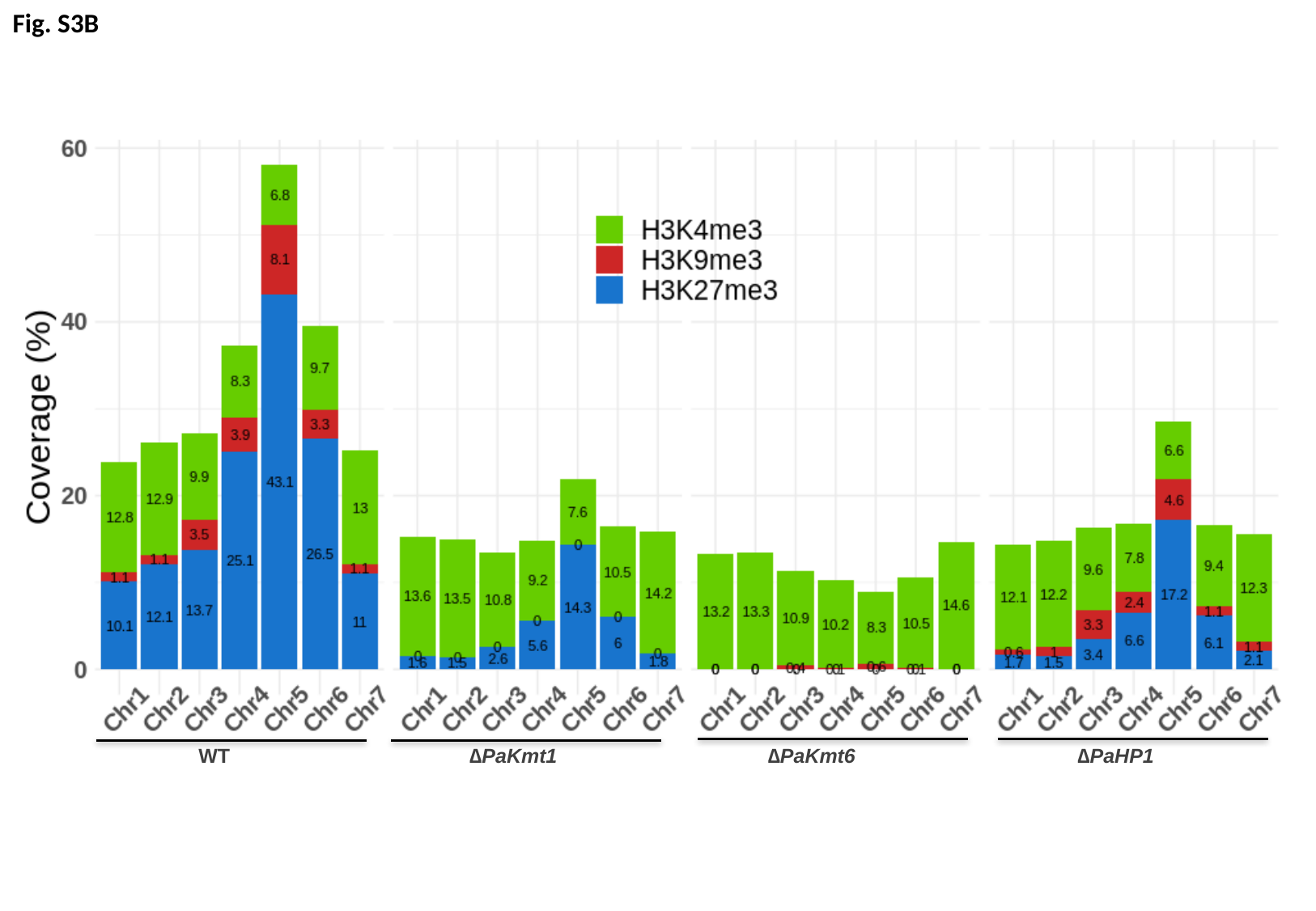

Fig. S3B
WT
∆PaKmt1
∆PaKmt6
∆PaHP1

## Slide 3
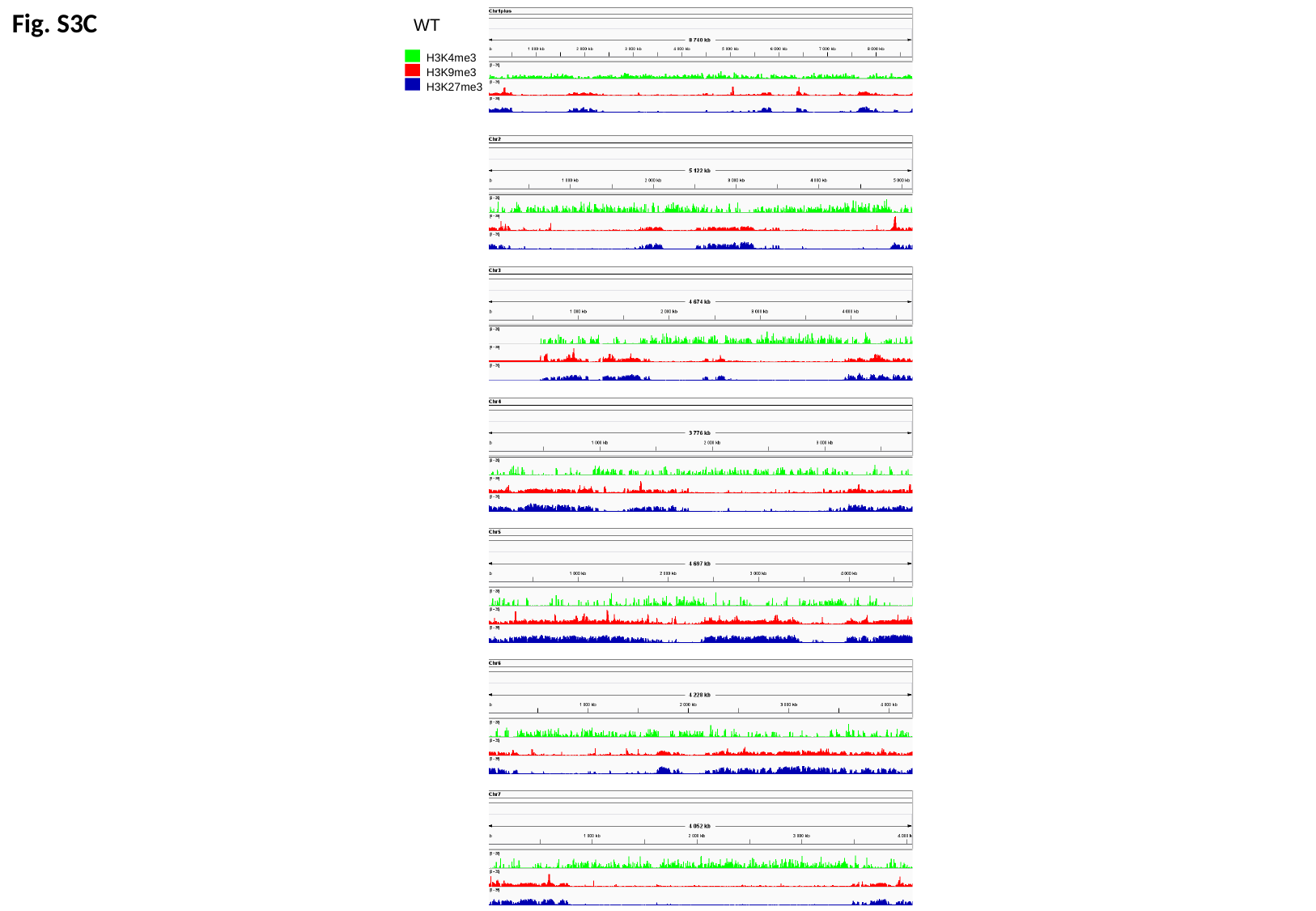

Fig. S3C
WT
H3K4me3
H3K9me3
H3K27me3

## Slide 4
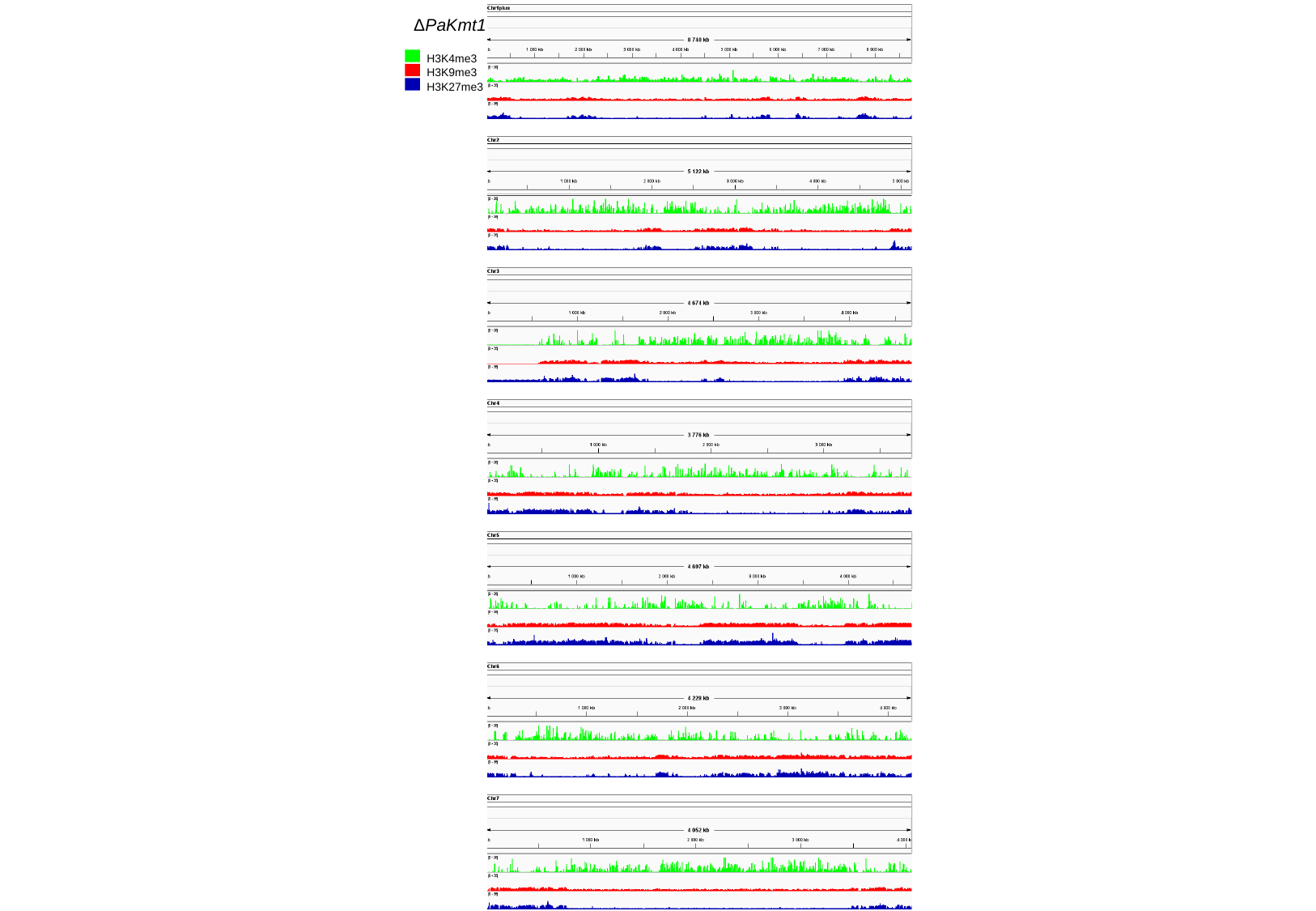

ΔPaKmt1
H3K4me3
H3K9me3
H3K27me3

## Slide 5
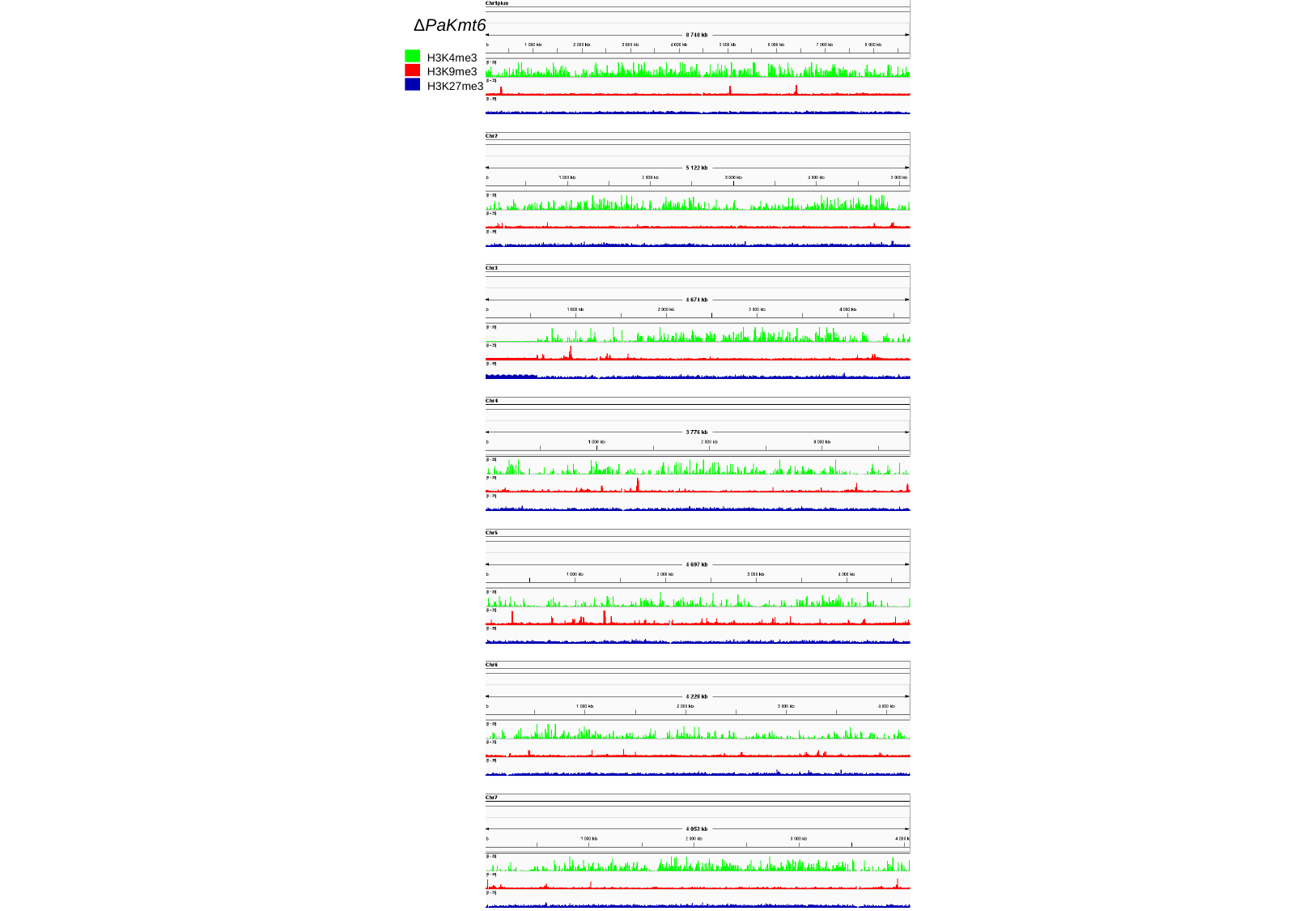

ΔPaKmt6
H3K4me3
H3K9me3
H3K27me3

## Slide 6
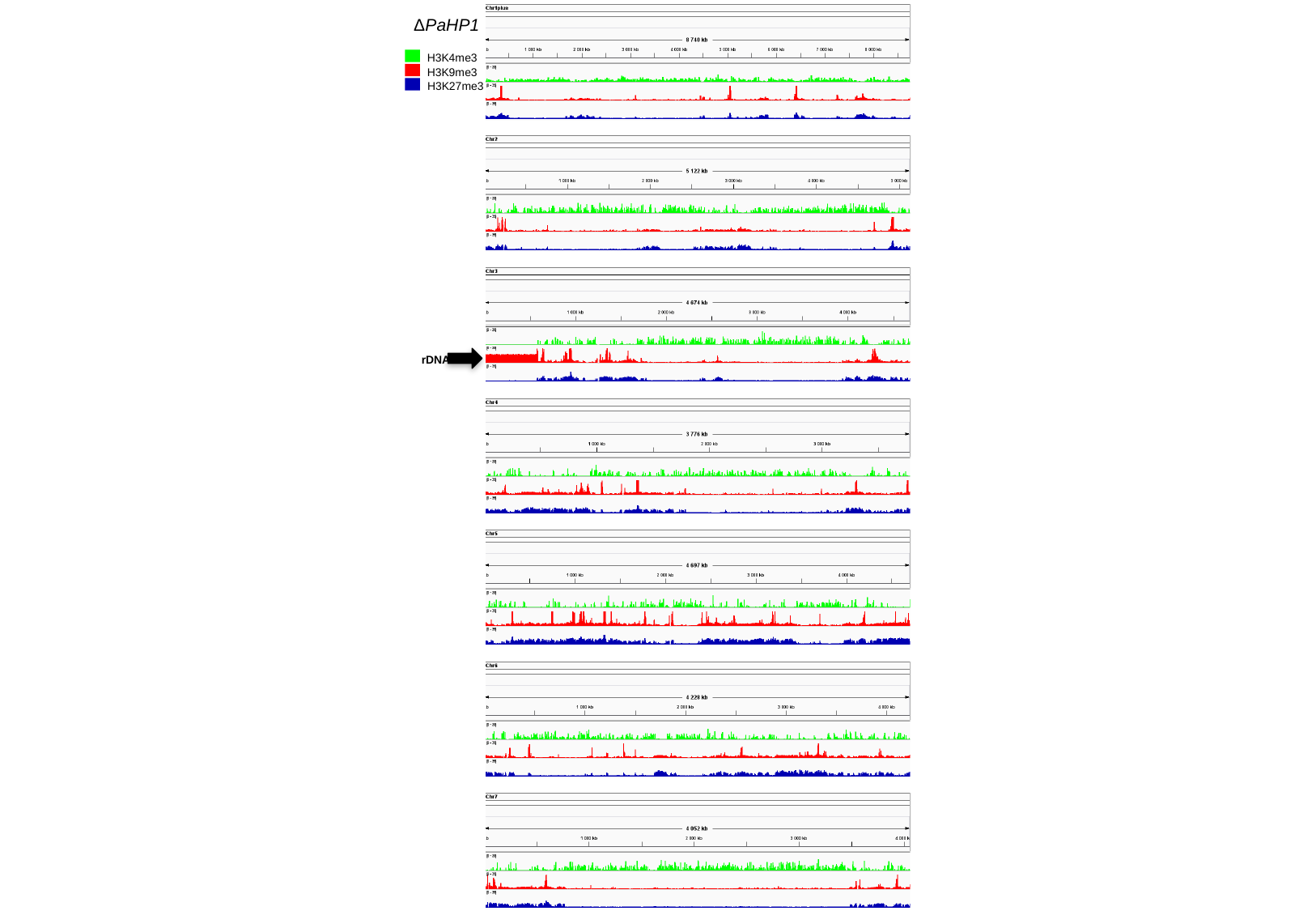

ΔPaHP1
H3K4me3
H3K9me3
H3K27me3
rDNA

## Slide 7
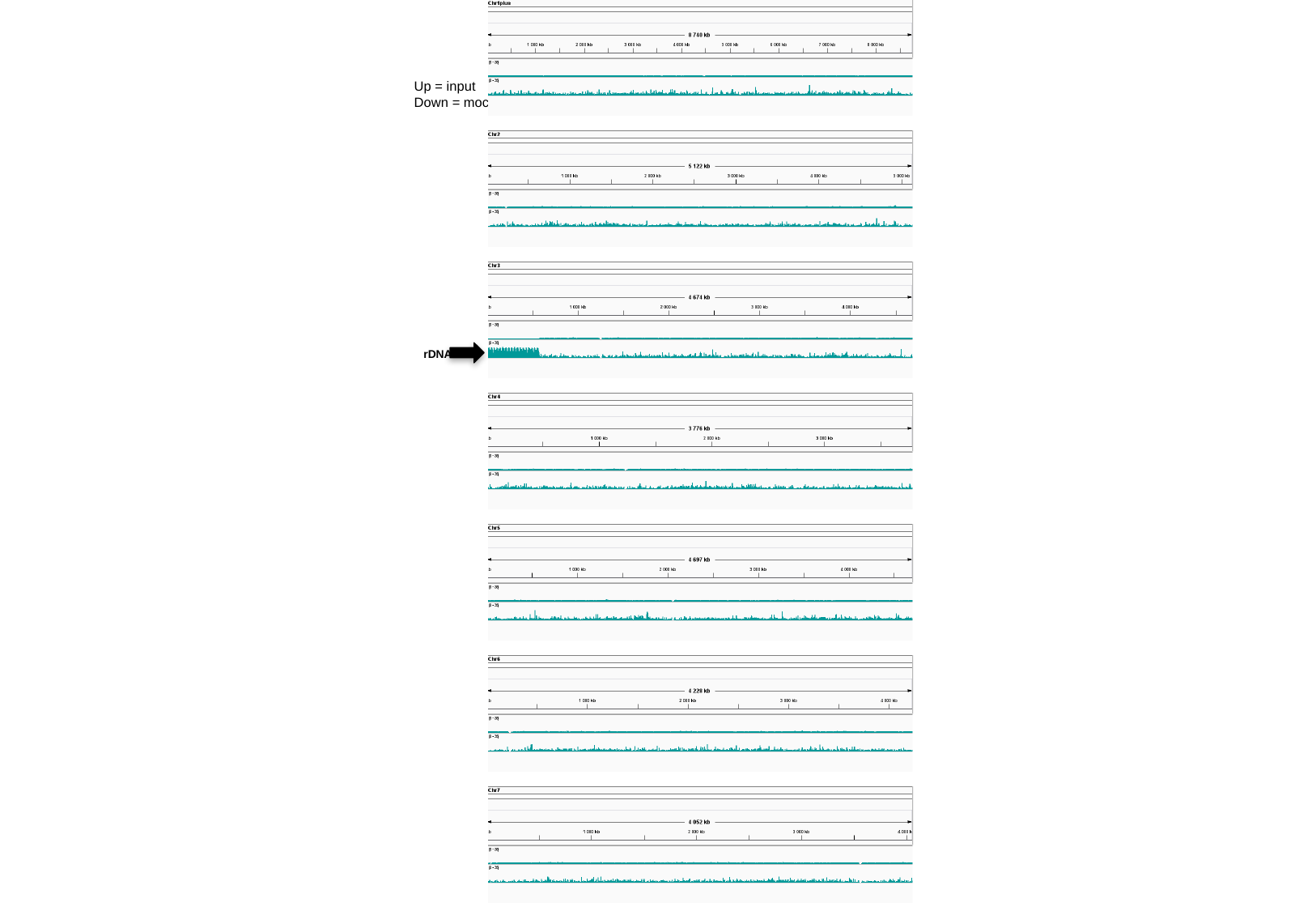

Up = input
Down = mock
rDNA

## Slide 8
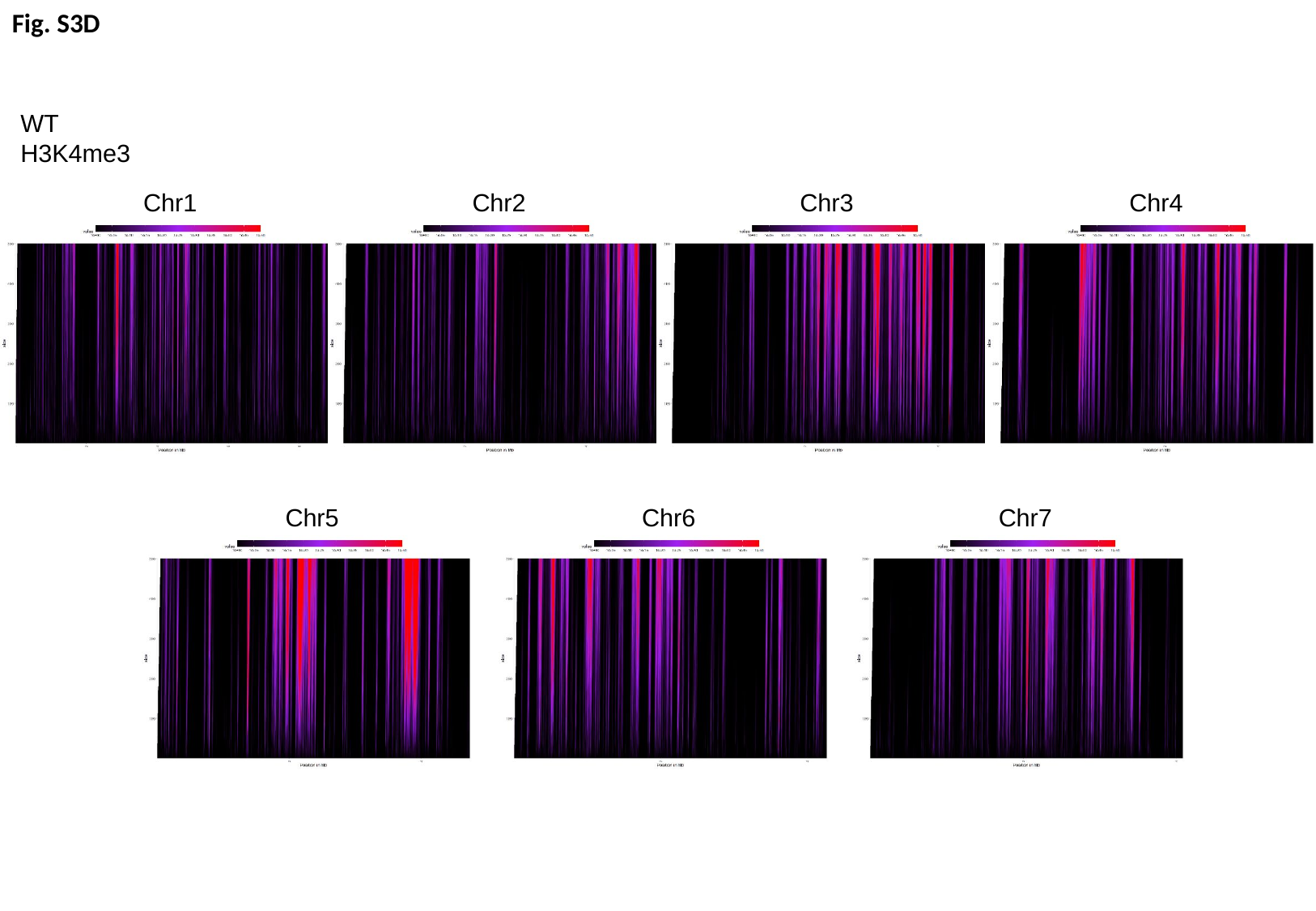

Fig. S3D
WT
H3K4me3
Chr1
Chr2
Chr3
Chr4
Chr5
Chr6
Chr7

## Slide 9
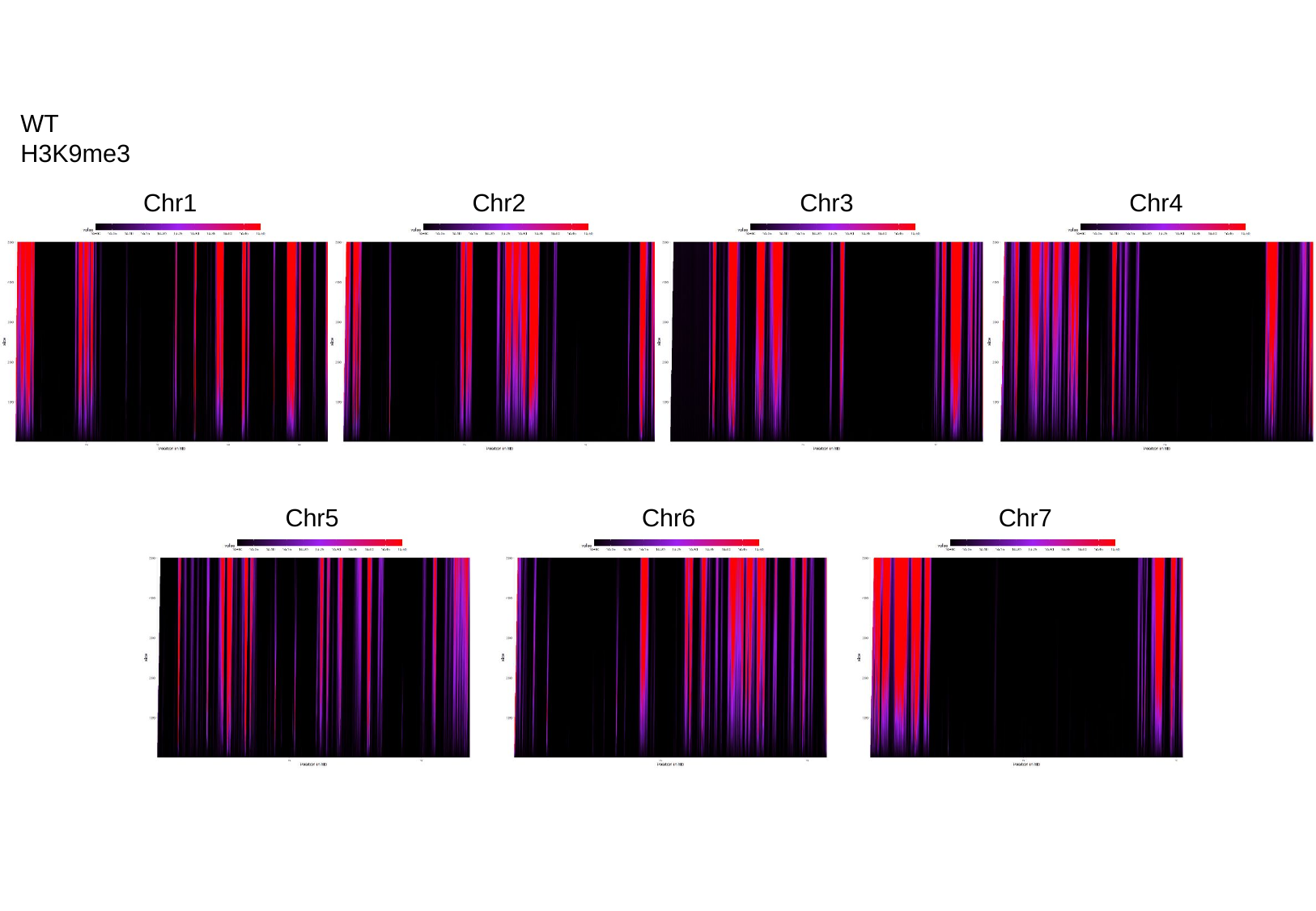

WT
H3K9me3
Chr1
Chr2
Chr3
Chr4
Chr5
Chr6
Chr7

## Slide 10
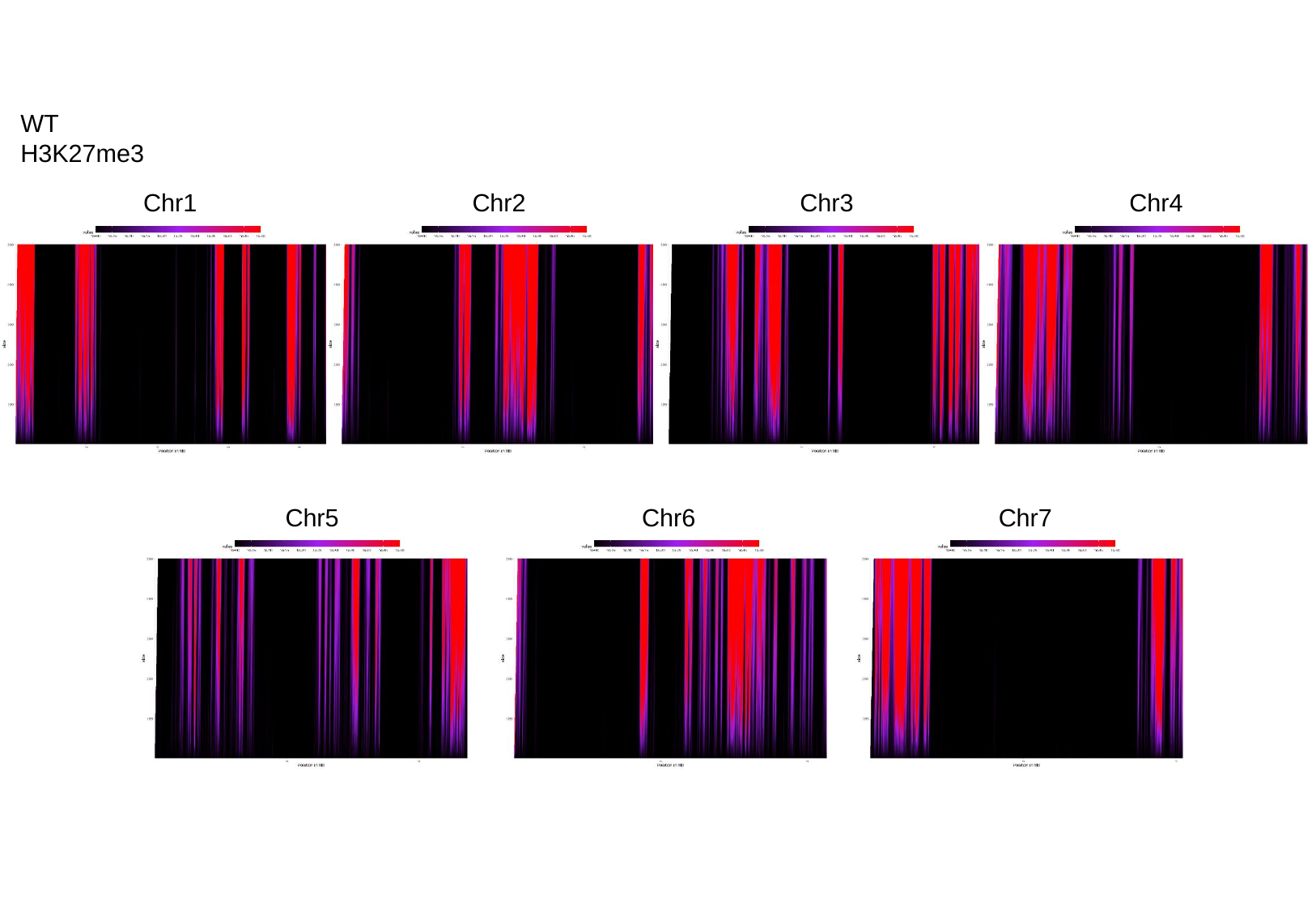

WT
H3K27me3
Chr1
Chr2
Chr3
Chr4
Chr5
Chr6
Chr7

## Slide 11
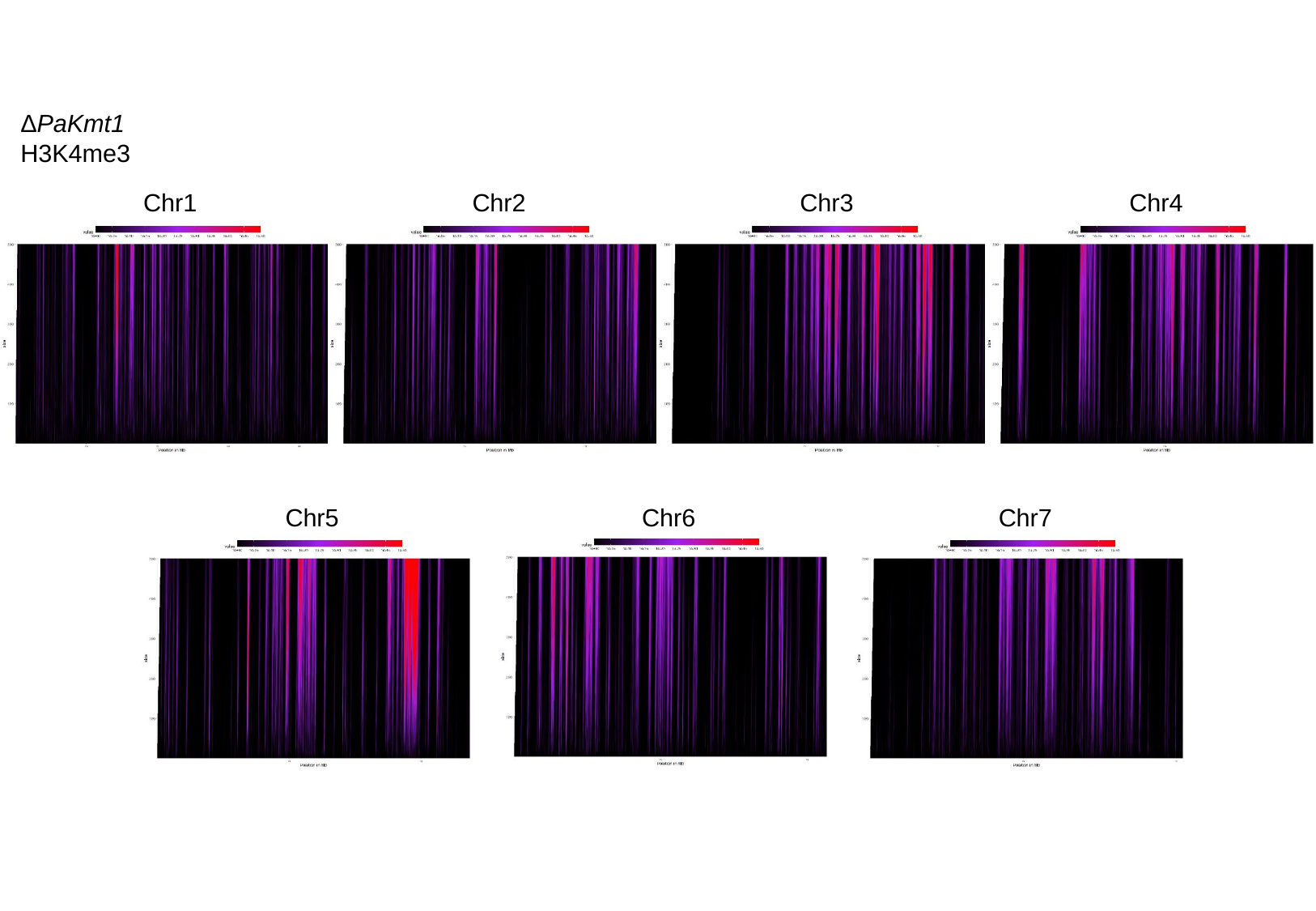

ΔPaKmt1
H3K4me3
Chr1
Chr2
Chr3
Chr4
Chr5
Chr6
Chr7

## Slide 12
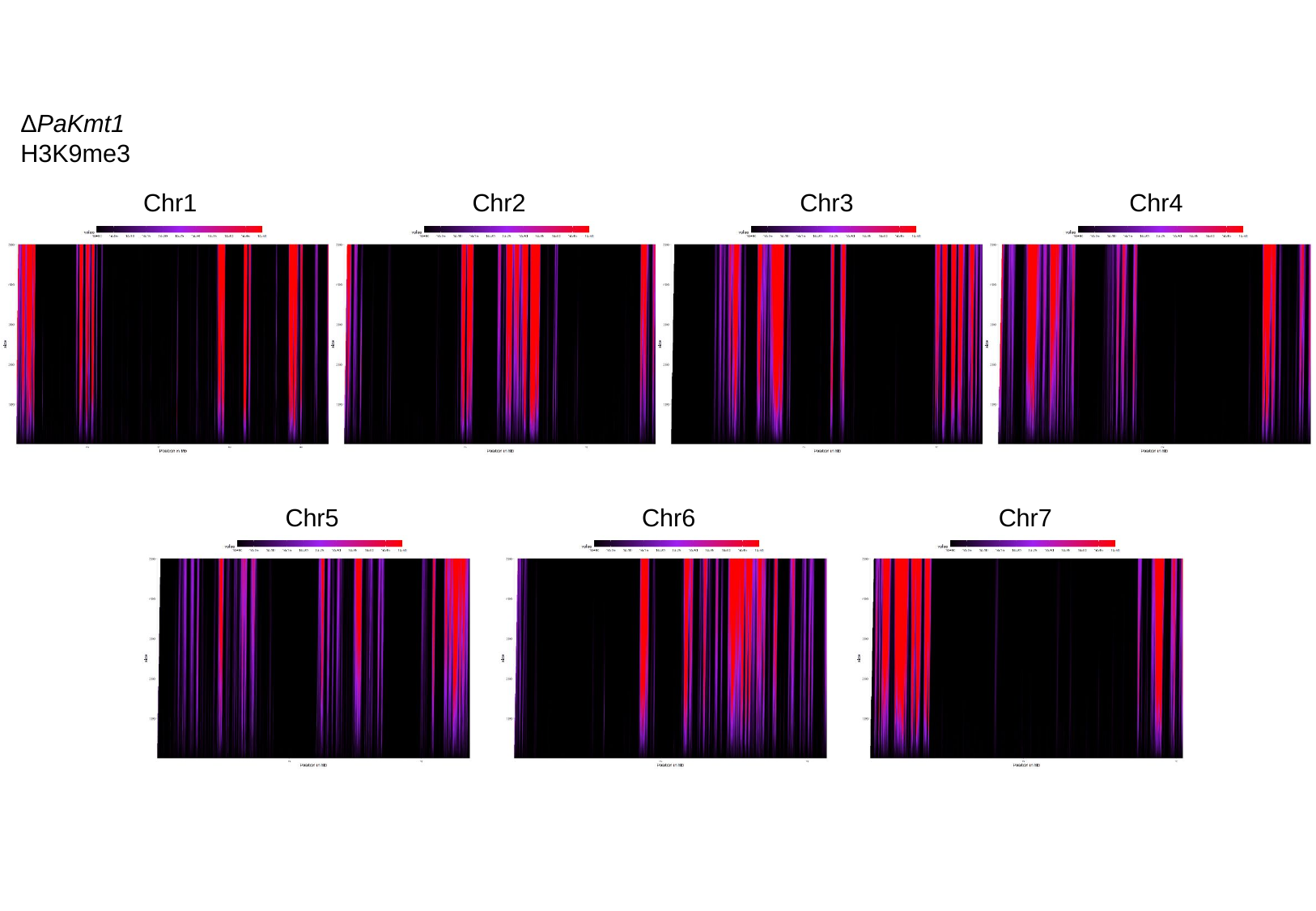

ΔPaKmt1
H3K9me3
Chr1
Chr2
Chr3
Chr4
Chr5
Chr6
Chr7

## Slide 13
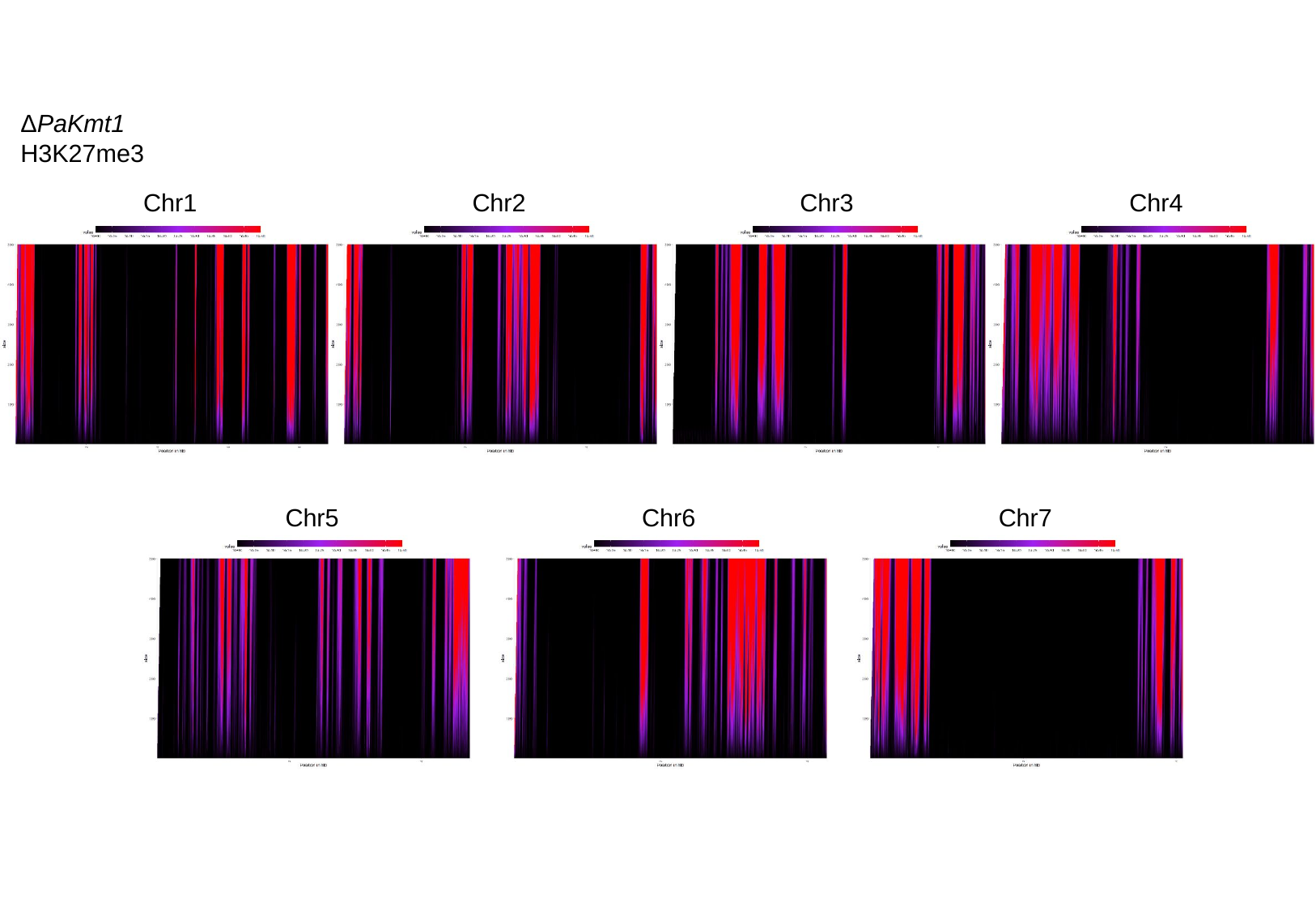

ΔPaKmt1
H3K27me3
Chr1
Chr2
Chr3
Chr4
Chr5
Chr6
Chr7

## Slide 14
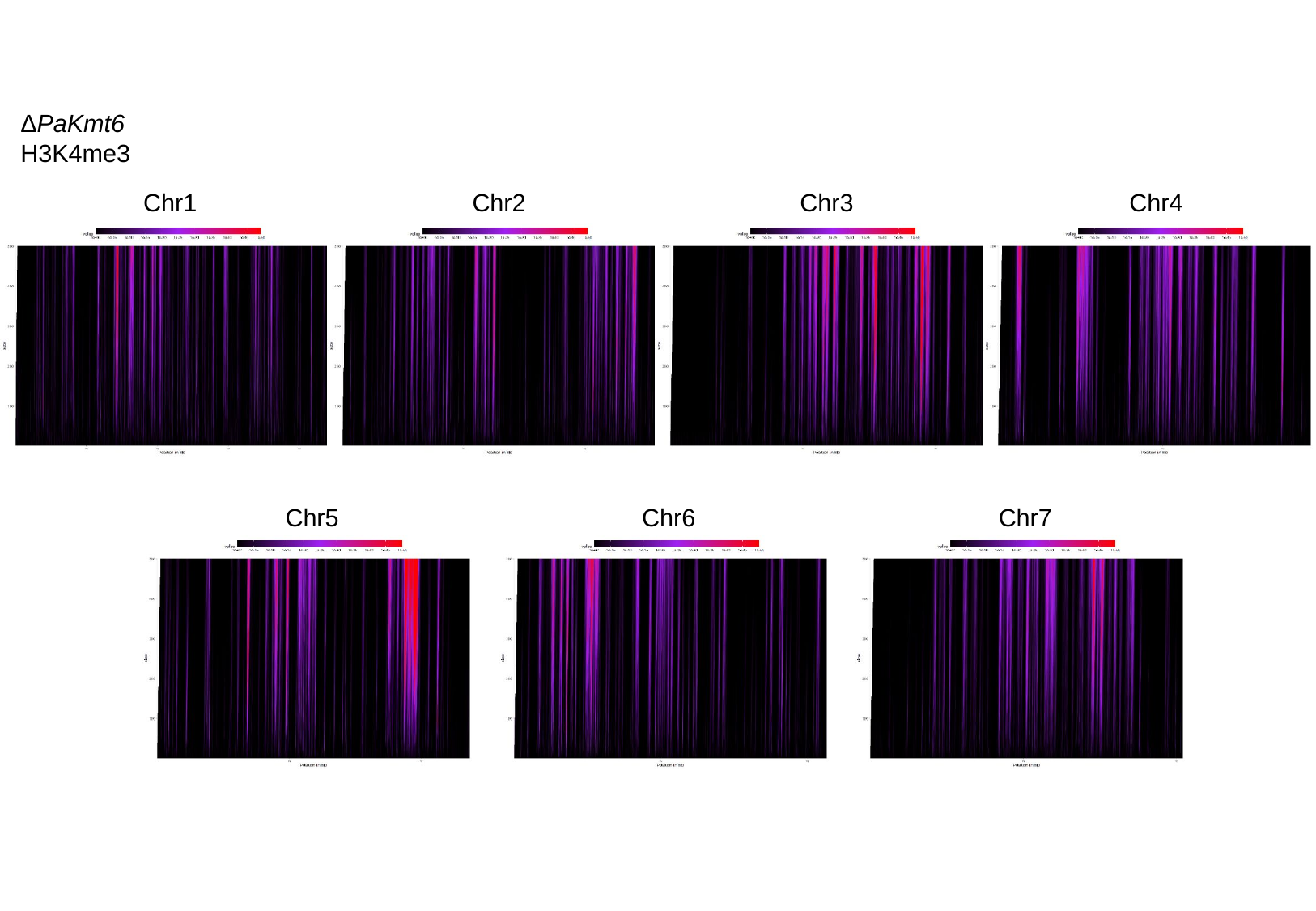

ΔPaKmt6
H3K4me3
Chr1
Chr2
Chr3
Chr4
Chr5
Chr6
Chr7

## Slide 15
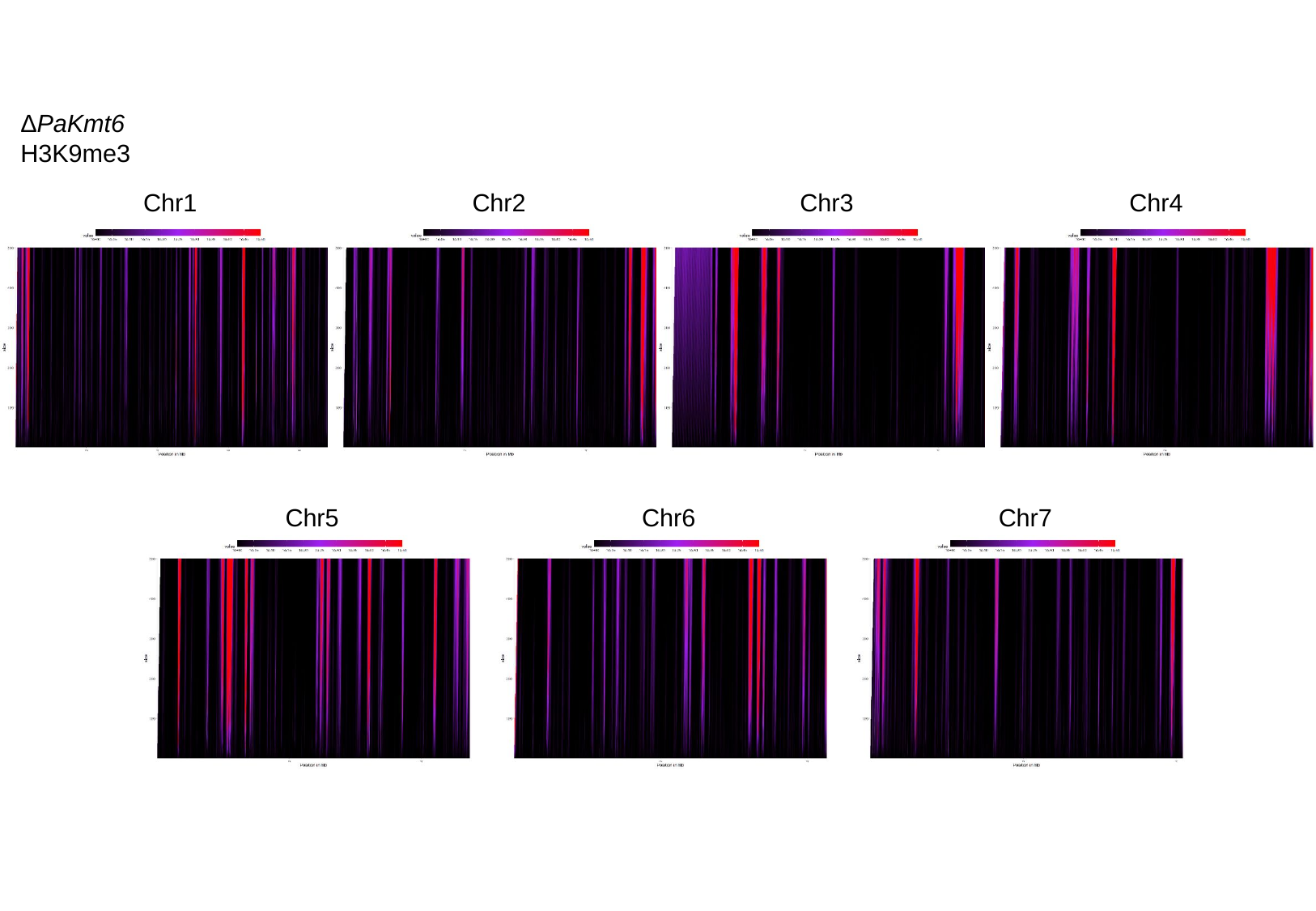

ΔPaKmt6
H3K9me3
Chr1
Chr2
Chr3
Chr4
Chr5
Chr6
Chr7

## Slide 16
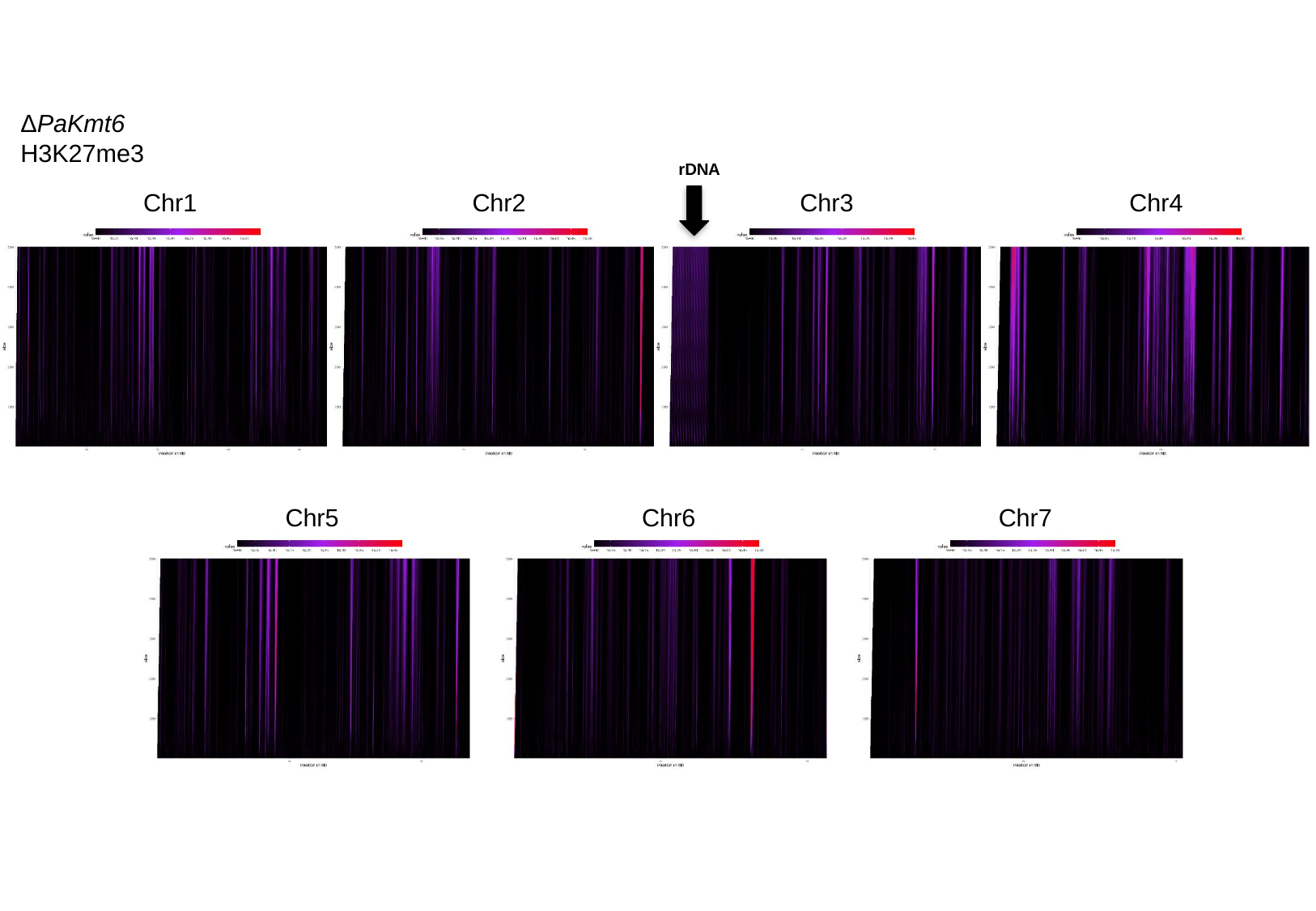

ΔPaKmt6
H3K27me3
rDNA
Chr1
Chr2
Chr3
Chr4
Chr5
Chr6
Chr7

## Slide 17
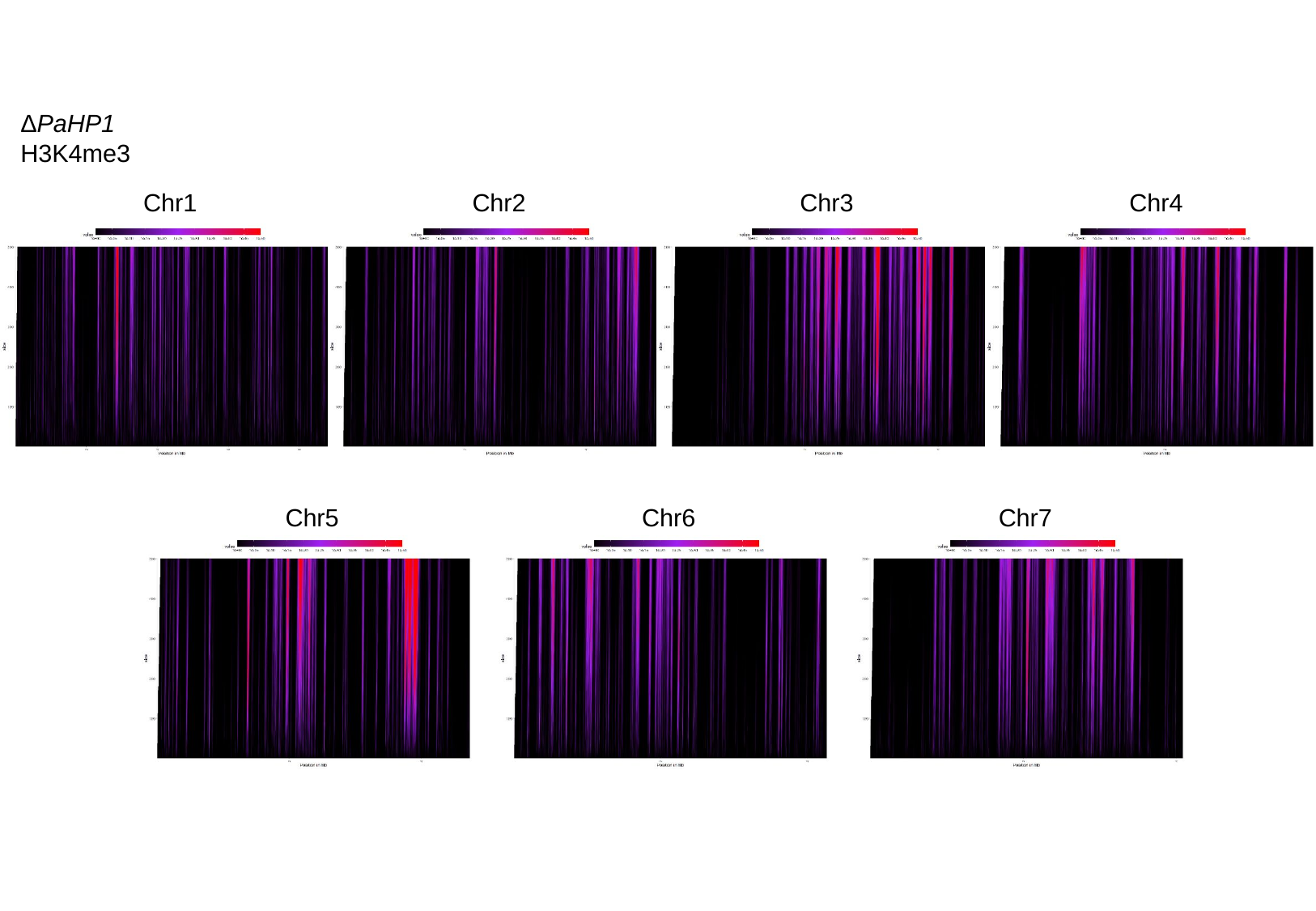

ΔPaHP1
H3K4me3
Chr1
Chr2
Chr3
Chr4
Chr5
Chr6
Chr7

## Slide 18
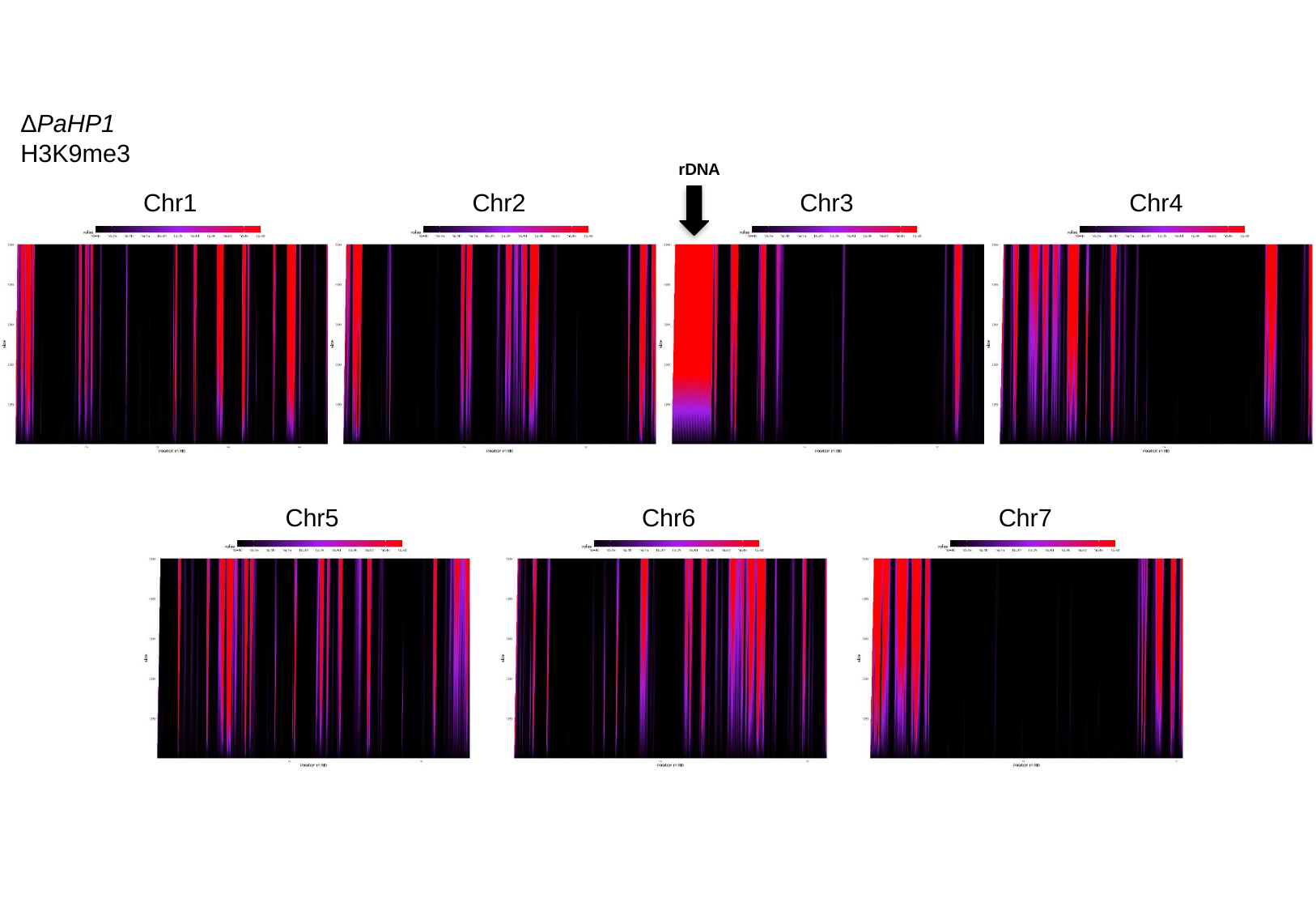

ΔPaHP1
H3K9me3
rDNA
Chr1
Chr2
Chr3
Chr4
Chr5
Chr6
Chr7

## Slide 19
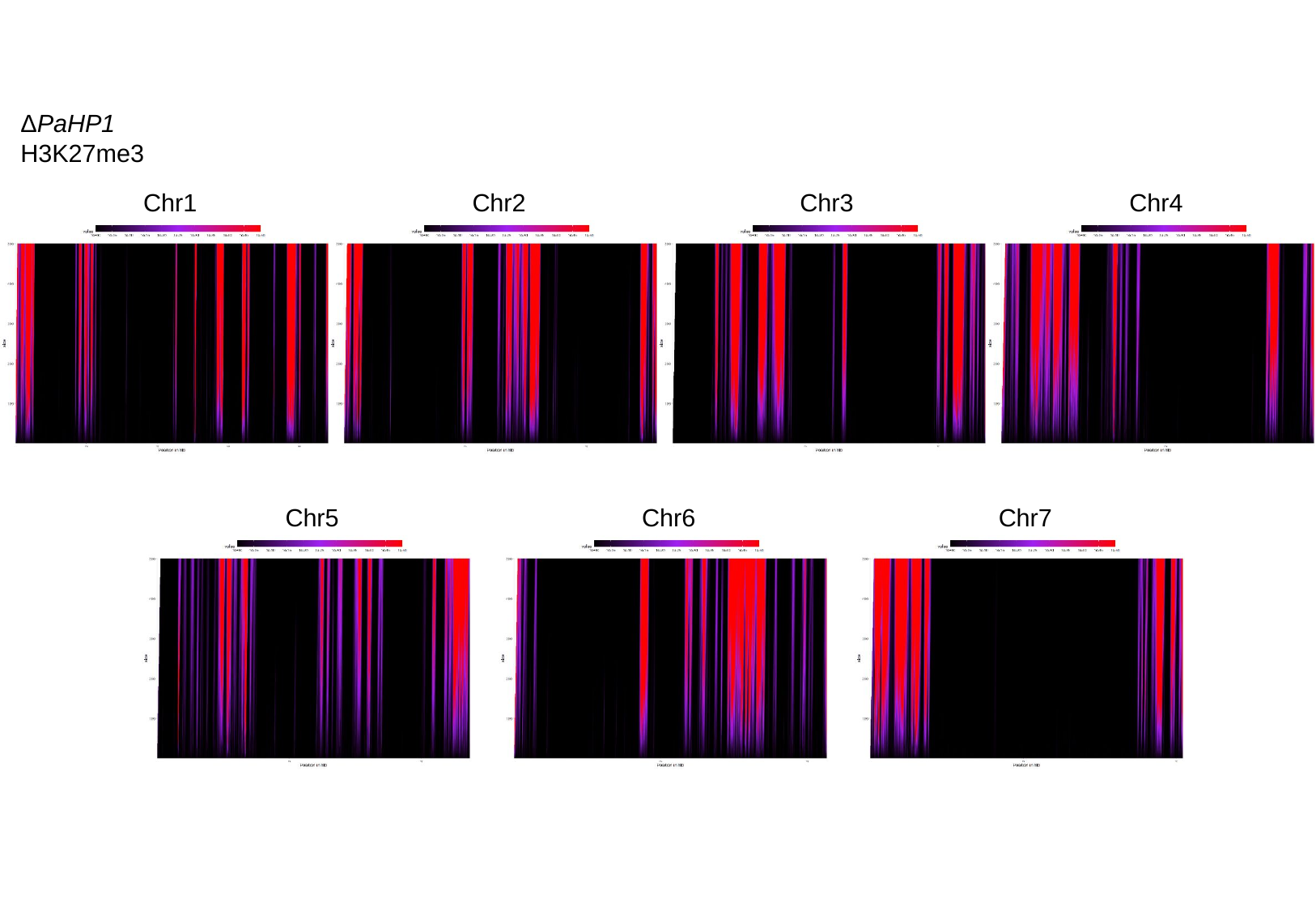

ΔPaHP1
H3K27me3
Chr1
Chr2
Chr3
Chr4
Chr5
Chr6
Chr7

## Slide 20
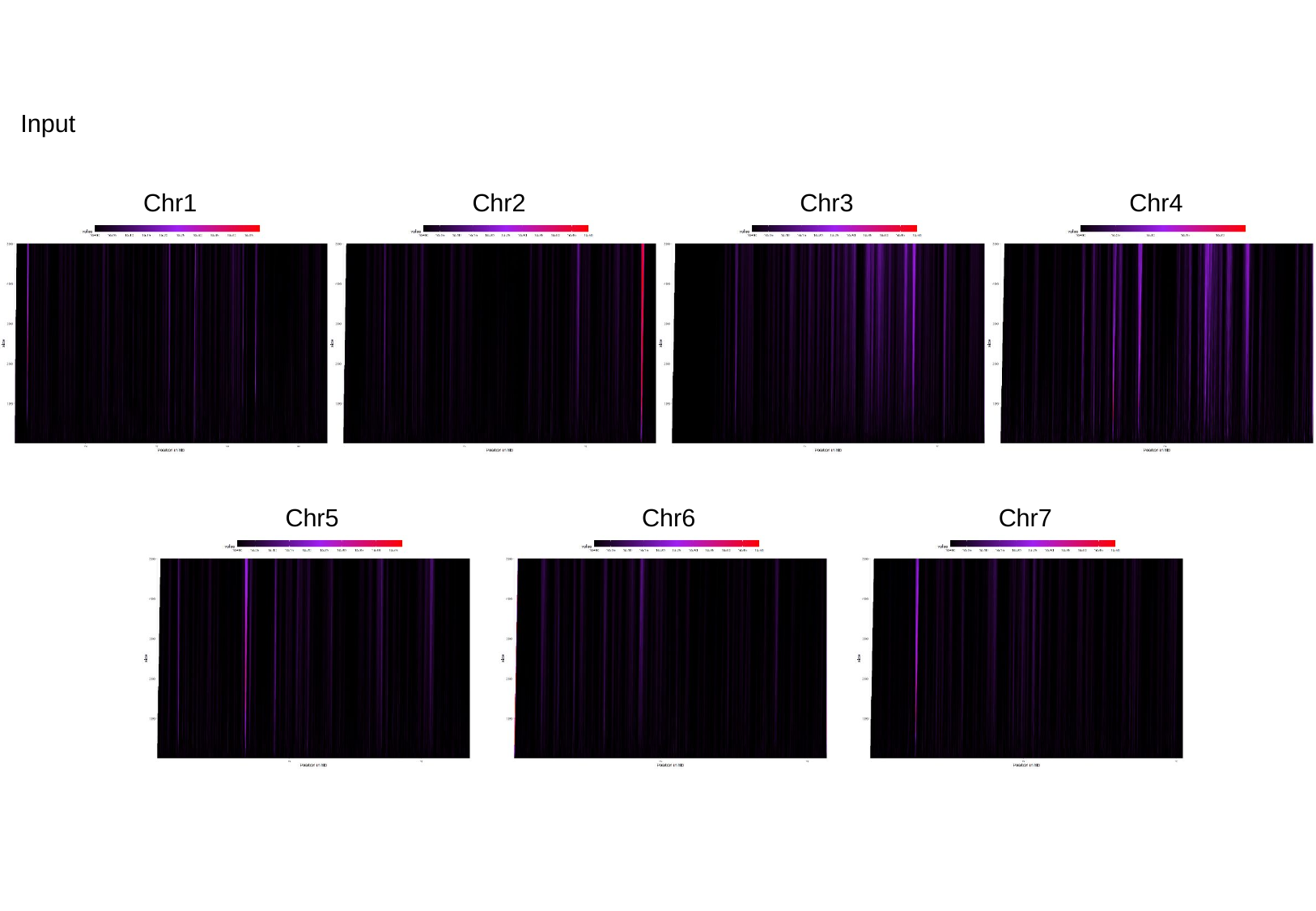

Input
Chr1
Chr2
Chr3
Chr4
Chr5
Chr6
Chr7

## Slide 21
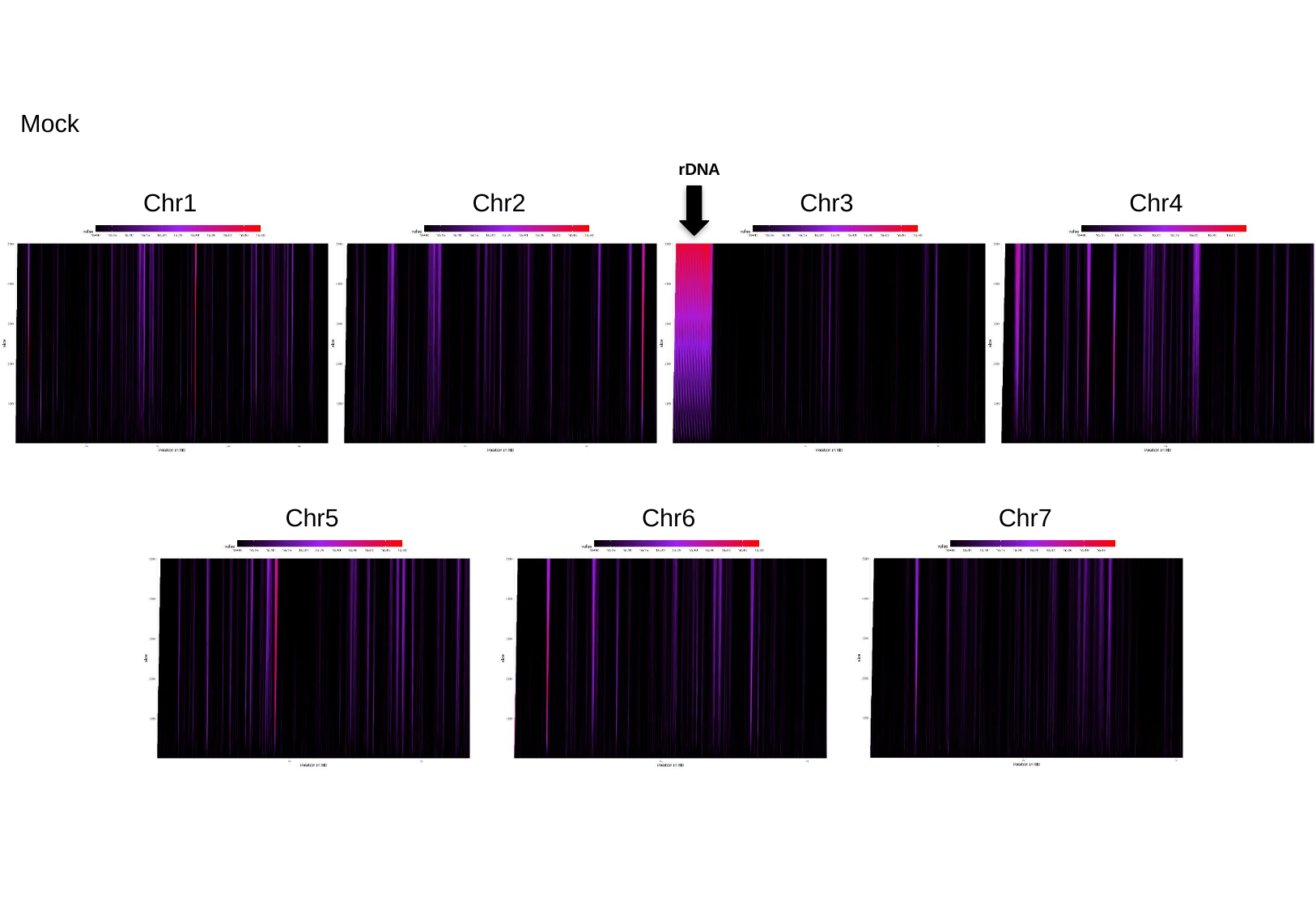

Mock
rDNA
Chr1
Chr2
Chr3
Chr4
Chr5
Chr6
Chr7
