# Supplementary material for: Loss of EZH2-like or SU(VAR)3–9-like proteins causes simultaneous perturbations in H3K27 and H3K9 tri-methylation and associated developmental defects in the fungus Podospora anserina
Source: Epigenetics Chromatin. 2021 May 7;14:22. doi: 10.1186/s13072-021-00395-7 (PMC8105982; doi:10.1186/s13072-021-00395-7)
Supplement: Supplementary file 4 — Additional file 4: Figure S4. Combined epigenetic landscapes in wild-type and heterochromatin mutant strains of P. anserina. Panorama of genome-wide peak localization for each genotype, wild-type, ΔPaKmt1, ΔPaKmt6 and ΔPaHP1 strains. Telomeres sequences were arbitrarily defined as the segment going from the end of each arm of the chromosomes to the first annotated gene (with the exception of the rDNA cluster localized on chromosome 3) and centromeres are indicated. Mat region = Non-recombining region containing the mating-type locus as defined in [93]. A segment overlapping portions of chromosomes 3 and 4 is expanded to show a zoom of the combined epigenetic landscapes. [file 13072_2021_395_MOESM4_ESM.pptx]

## Slide 1
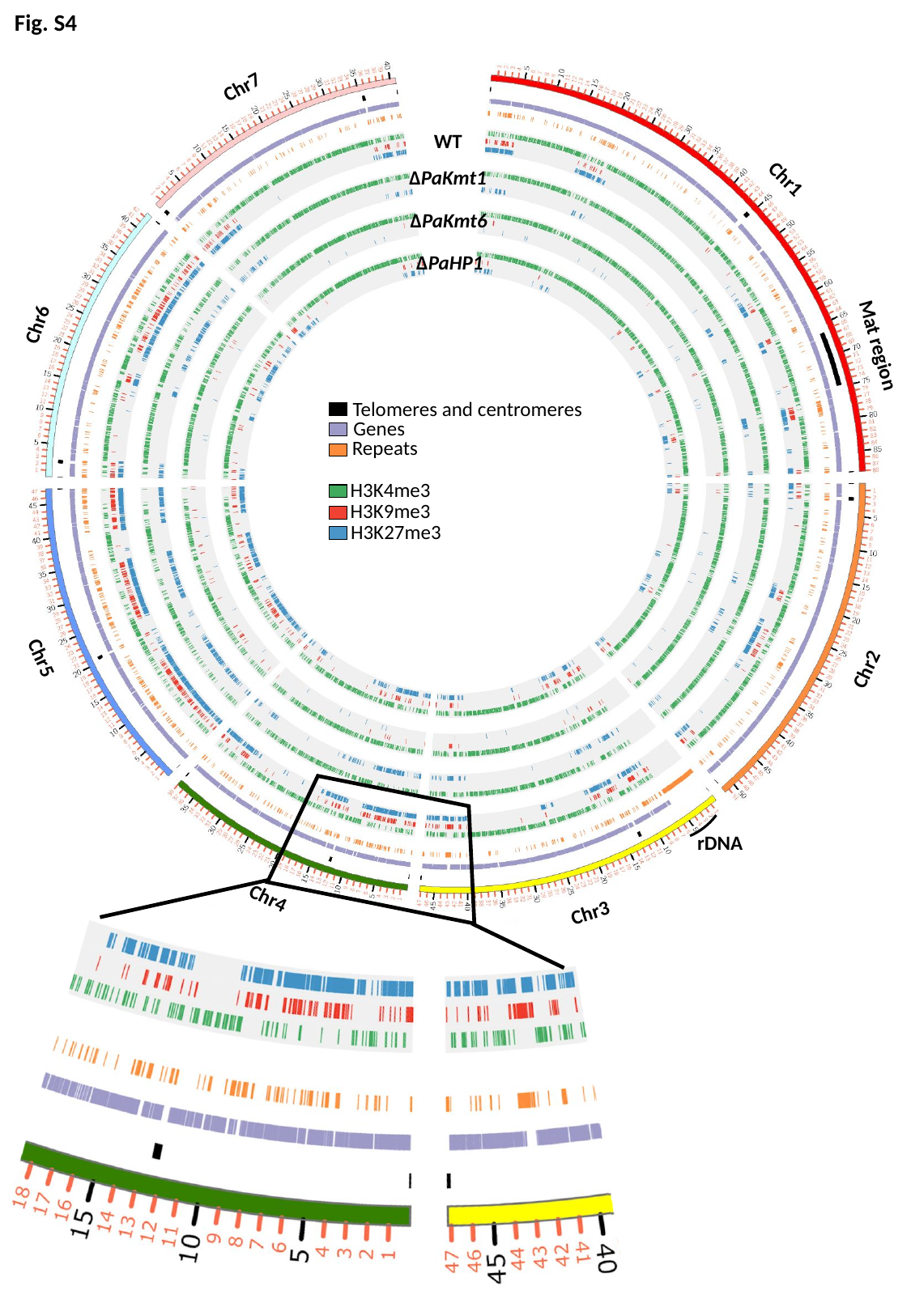

Fig. S4
Chr7
WT
∆PaKmt1
Chr1
∆PaKmt6
∆PaHP1
Chr6
Mat region
Telomeres and centromeres
Genes
Repeats
H3K4me3
H3K9me3
H3K27me3
Chr5
Chr2
rDNA
Chr4
Chr3
