# Supplementary material for: Loss of EZH2-like or SU(VAR)3–9-like proteins causes simultaneous perturbations in H3K27 and H3K9 tri-methylation and associated developmental defects in the fungus Podospora anserina
Source: Epigenetics Chromatin. 2021 May 7;14:22. doi: 10.1186/s13072-021-00395-7 (PMC8105982; doi:10.1186/s13072-021-00395-7)
Supplement: Supplementary file 5 — Additional file 5: Figure S5. H3K4me3, H3K27me3 and H3K9me3 modifications of P. anserina TEs in the WT, ΔPaKmt1, ΔPaKmt6 and ΔPaHP1 mutant strains. A. Histone marks on P. anserina TE families. Top panel: Plots of normalized ChIP signal: H3K4me3 (green), H3K9me3 (red) and H3K27me3 (dark blue) signals in the wild-type strain for five TE families, i.e., Copia, Gypsy, MITE, Tc1 Mariner, solo LTR and unclassified TEs [51] (Additional file 21: Table S3). Because MITE TEs are shorter than the other categories (< 500 bp in length), the genomic window was narrowed on the graph. Aligned sequences correspond to TE bodies ± 0.5 kbp surrounding region (see Additional file 21: Table S3 for TE’s numbers). Bottom panel: K-means built clusters representing the association versus non-association of the indicated histone modifications within a specific TE family. Histone modification levels in the heatmaps were calculated for non-overlapping 10-bp windows within the specific genomic regions and sorted by average value of each row. B Number of TEs, classified by family, marked with H3K4me3, H3K27me3 or H3K9me3, according to genetic backgrounds (wild-type strain, ΔPaKmt1, ΔPaKmt6 and ΔPaHP1 mutant strains). C Violin plots of expression of TEs classified by family. Gene expression was inferred from the TPM (Transcripts Per Kilobase Million) values calculated in [49]. Gene expression of non-repeated CDS (i.e., “genes”) was added for comparison. [file 13072_2021_395_MOESM5_ESM.pptx]

## Slide 1
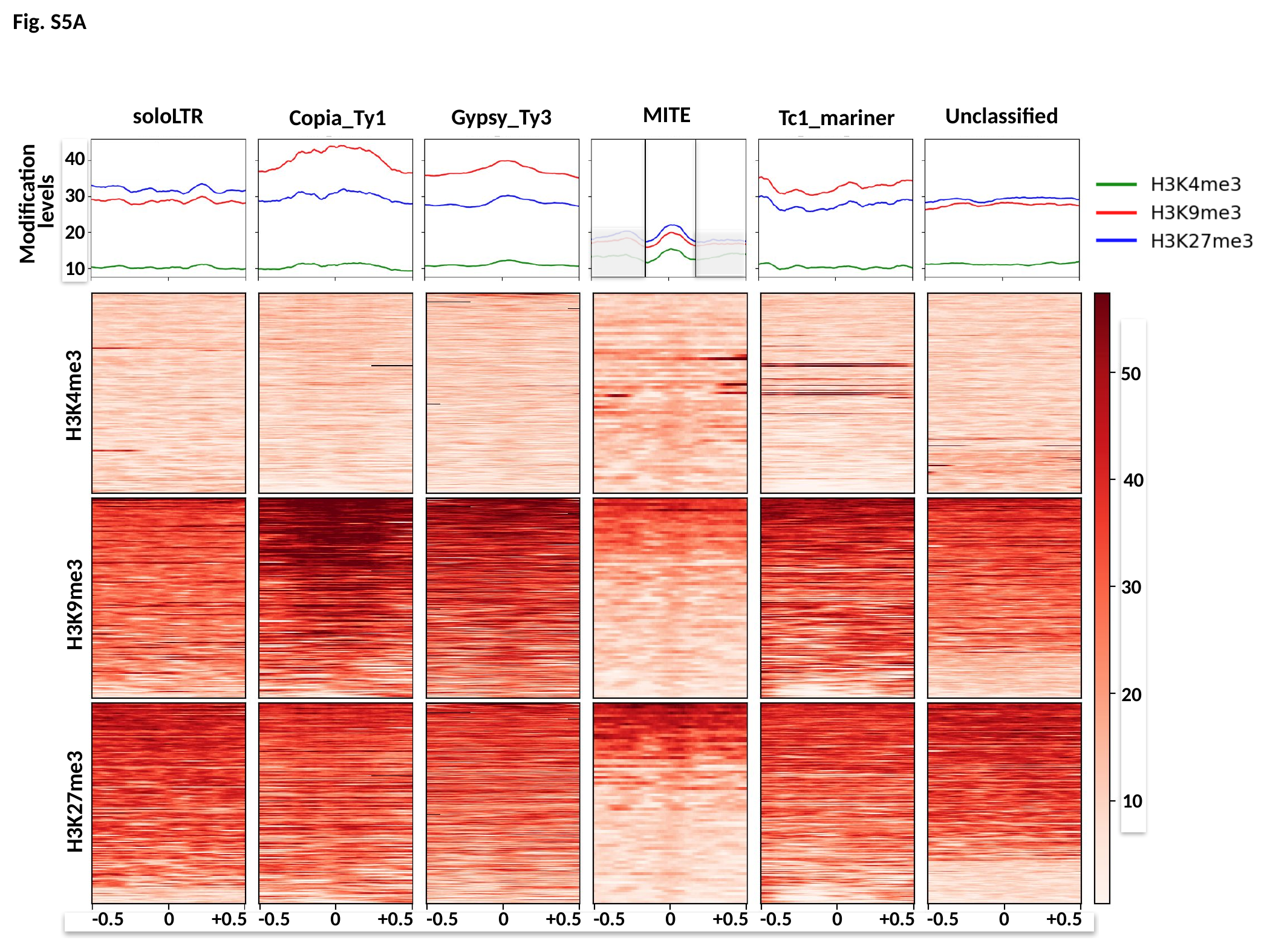

Fig. S5A
MITE
soloLTR
Unclassified
Gypsy_Ty3
Copia_Ty1
Tc1_mariner
40
30
Modification
 levels
20
10
50
H3K4me3
40
30
H3K9me3
20
H3K27me3
10
-0.5
0
+0.5
-0.5
0
+0.5
-0.5
0
+0.5
-0.5
0
+0.5
-0.5
0
+0.5
-0.5
0
+0.5

## Slide 2
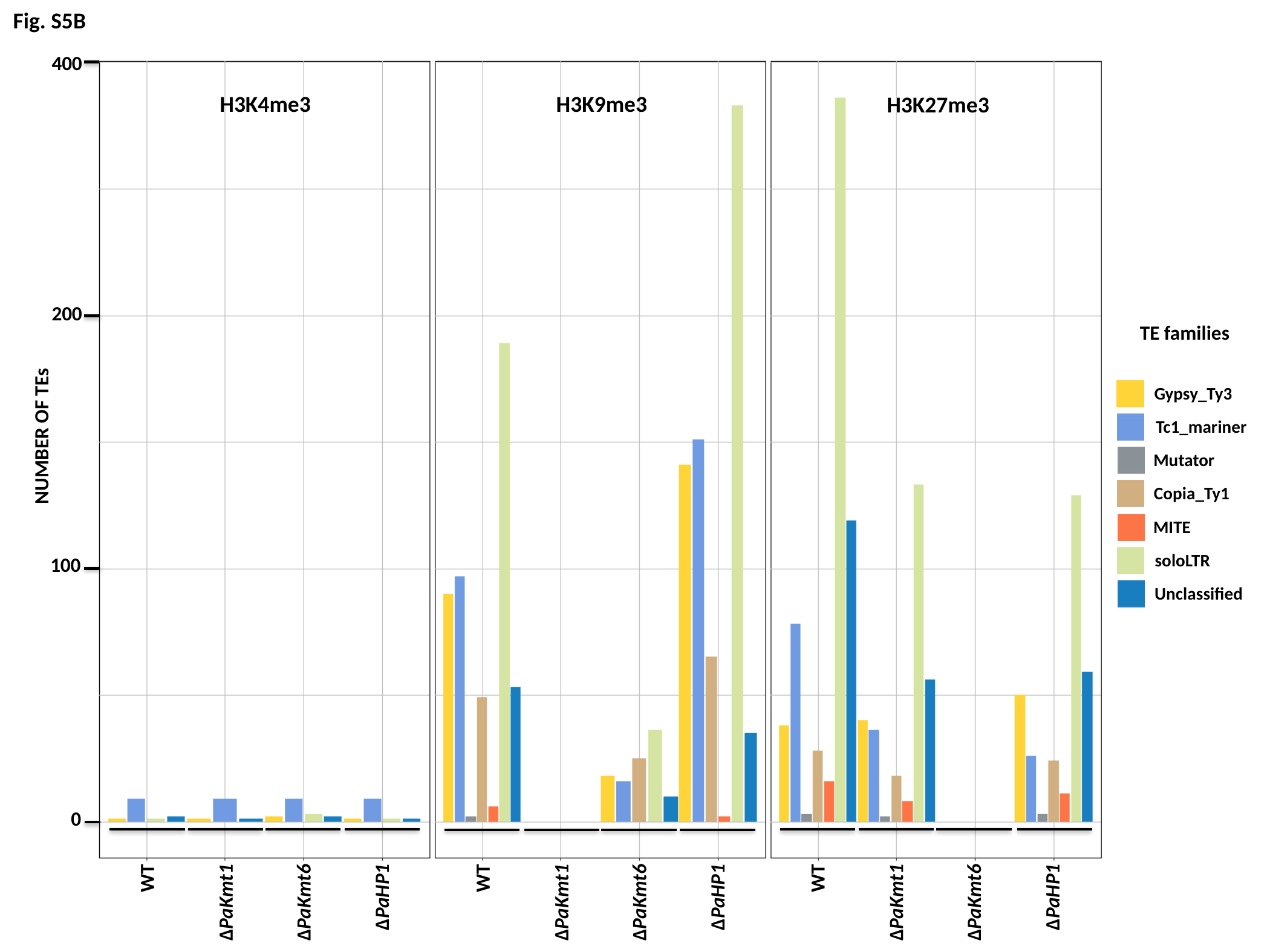

Fig. S5B
400
H3K4me3
H3K9me3
H3K27me3
200
TE families
Gypsy_Ty3
Tc1_mariner
Mutator
Copia_Ty1
MITE
soloLTR
Unclassified
NUMBER OF TEs
100
0
WT
WT
WT
ΔPaHP1
ΔPaHP1
ΔPaHP1
ΔPaKmt1
ΔPaKmt6
ΔPaKmt1
ΔPaKmt6
ΔPaKmt1
ΔPaKmt6

## Slide 3
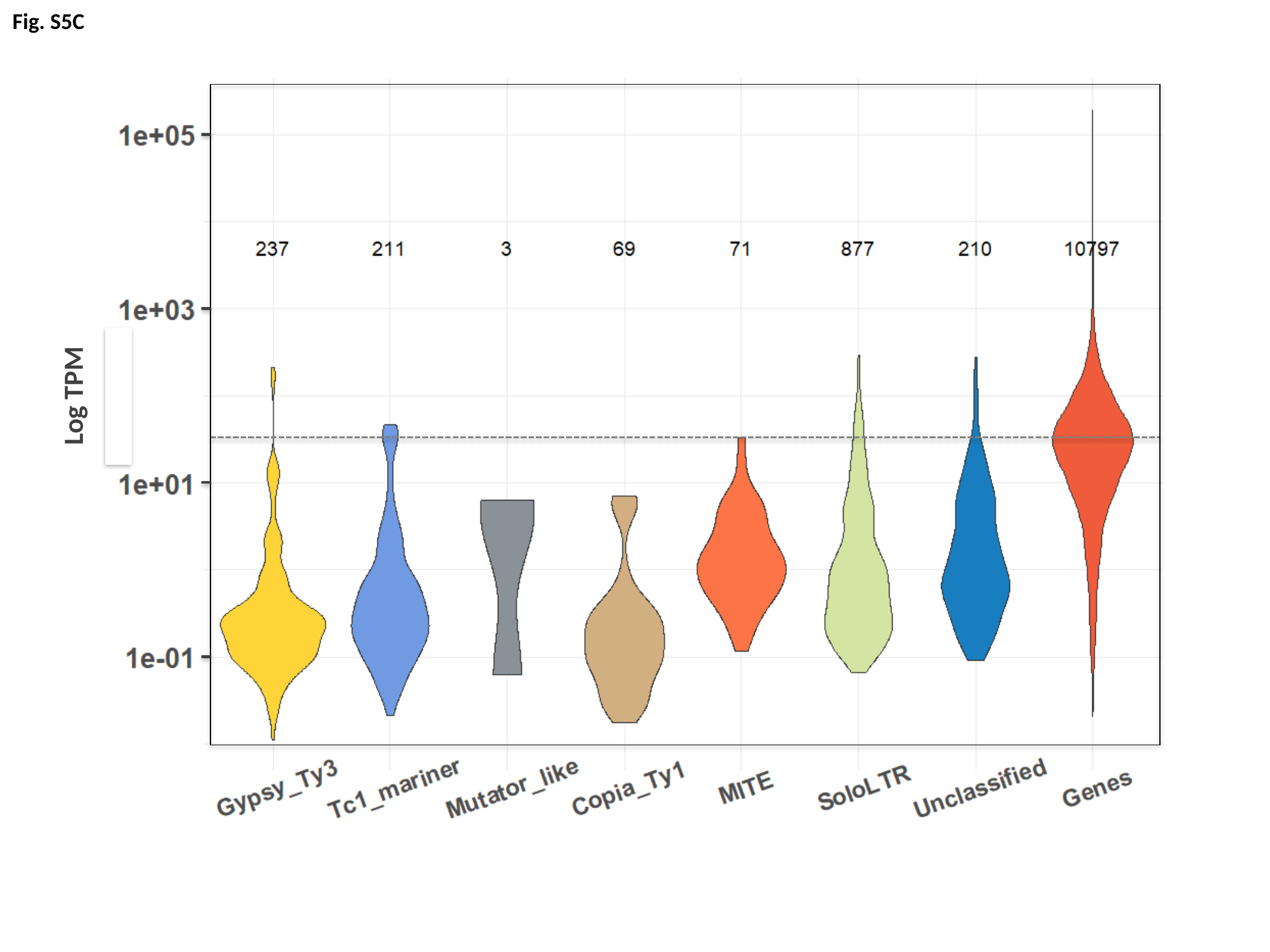

Fig. S5C
Log TPM
