# Supplementary material for: Loss of EZH2-like or SU(VAR)3–9-like proteins causes simultaneous perturbations in H3K27 and H3K9 tri-methylation and associated developmental defects in the fungus Podospora anserina
Source: Epigenetics Chromatin. 2021 May 7;14:22. doi: 10.1186/s13072-021-00395-7 (PMC8105982; doi:10.1186/s13072-021-00395-7)
Supplement: Supplementary file 6 — Additional file 6: Figure S6. Snapshots of a set of TEs representing all the annotated TE families in P. anserina’s genome. ChIP-seq signals were normalized as described in “Methods” and visualized using the Integrated Genomics Viewer (IGV) [115]. H3K4me3 (green), H3K9me3 (red) and H3K27me3 (blue). [file 13072_2021_395_MOESM6_ESM.pptx]

## Slide 1
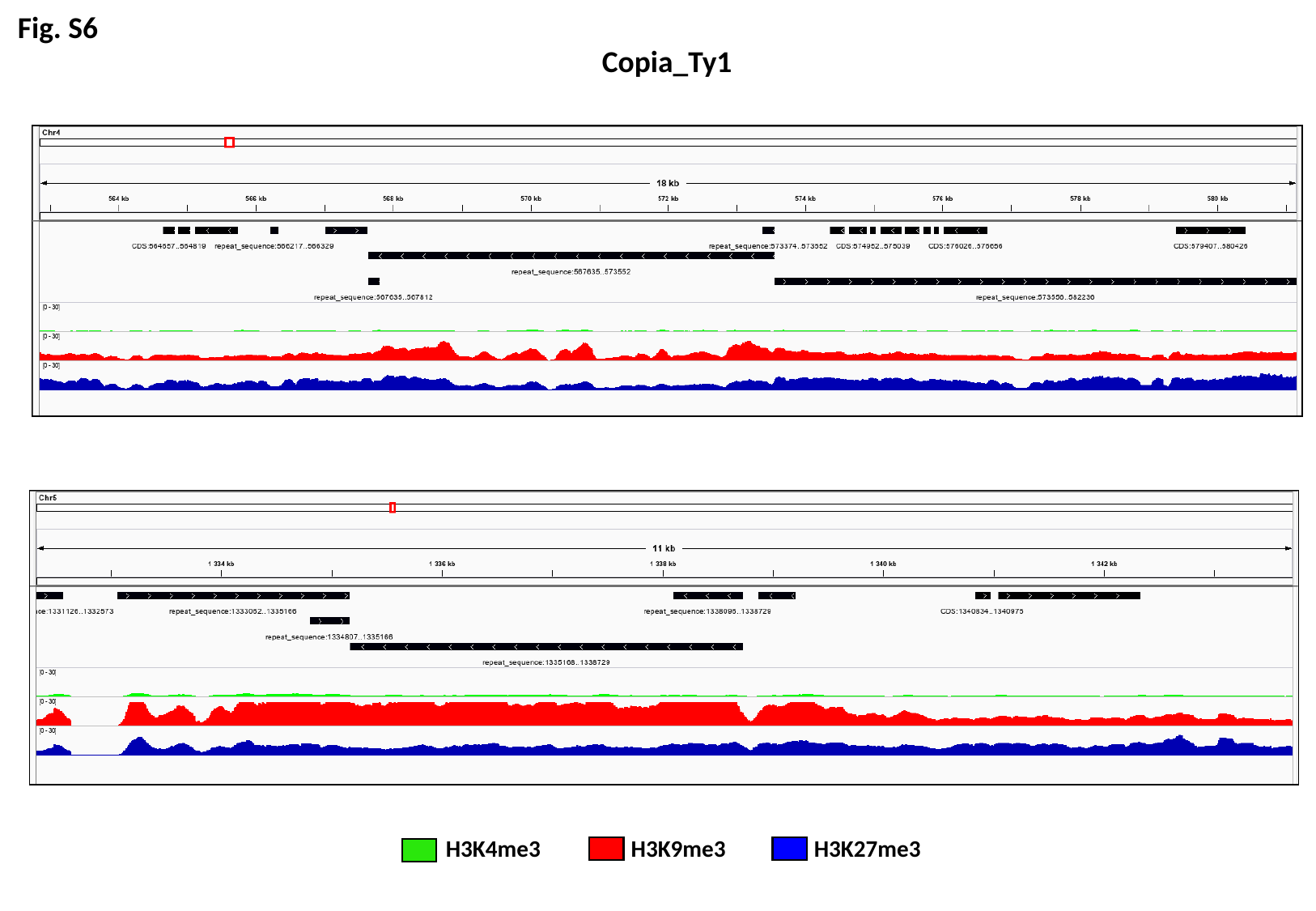

Fig. S6
Copia_Ty1
H3K4me3
H3K9me3
H3K27me3

## Slide 2
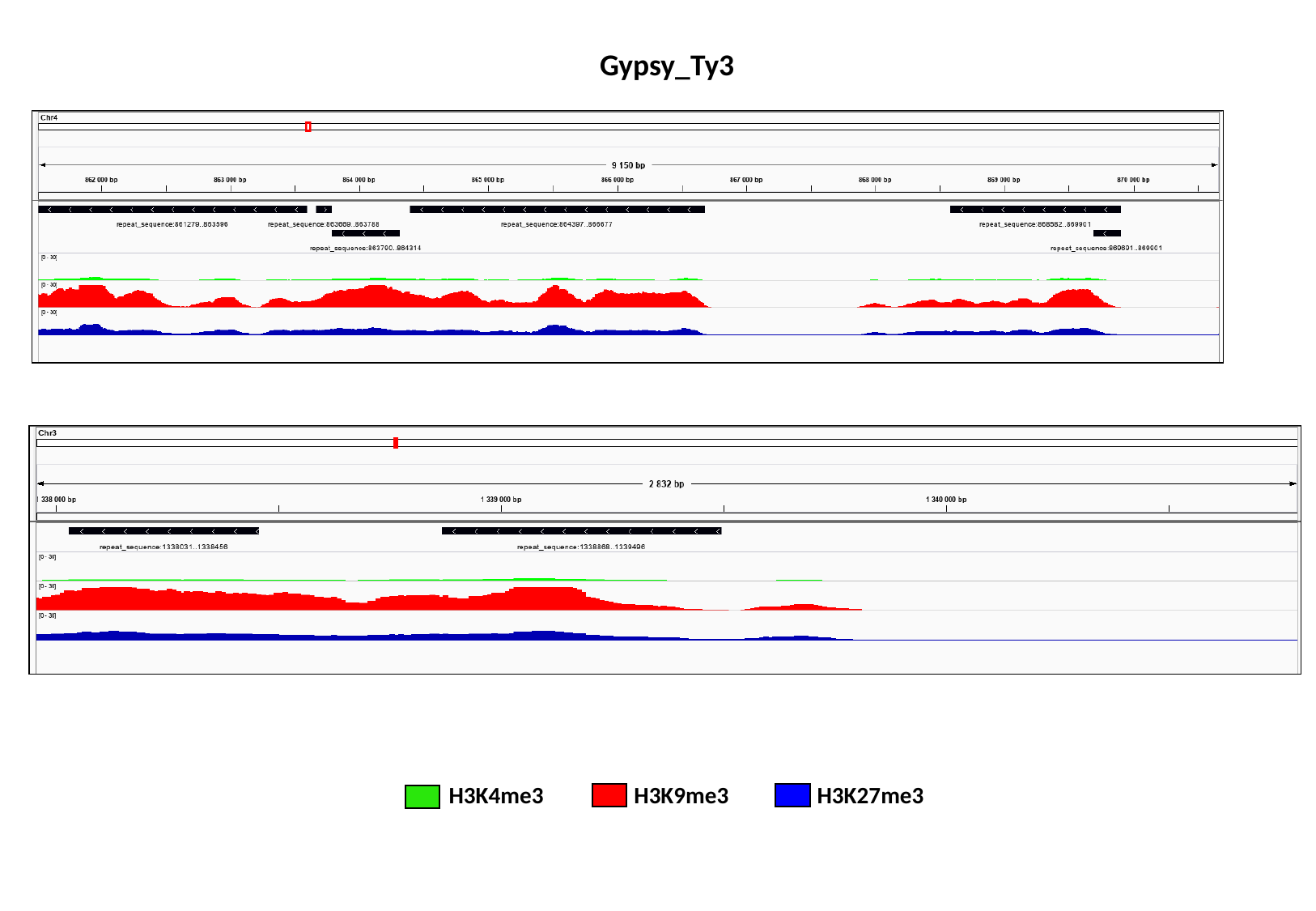

Gypsy_Ty3
H3K4me3
H3K9me3
H3K27me3

## Slide 3
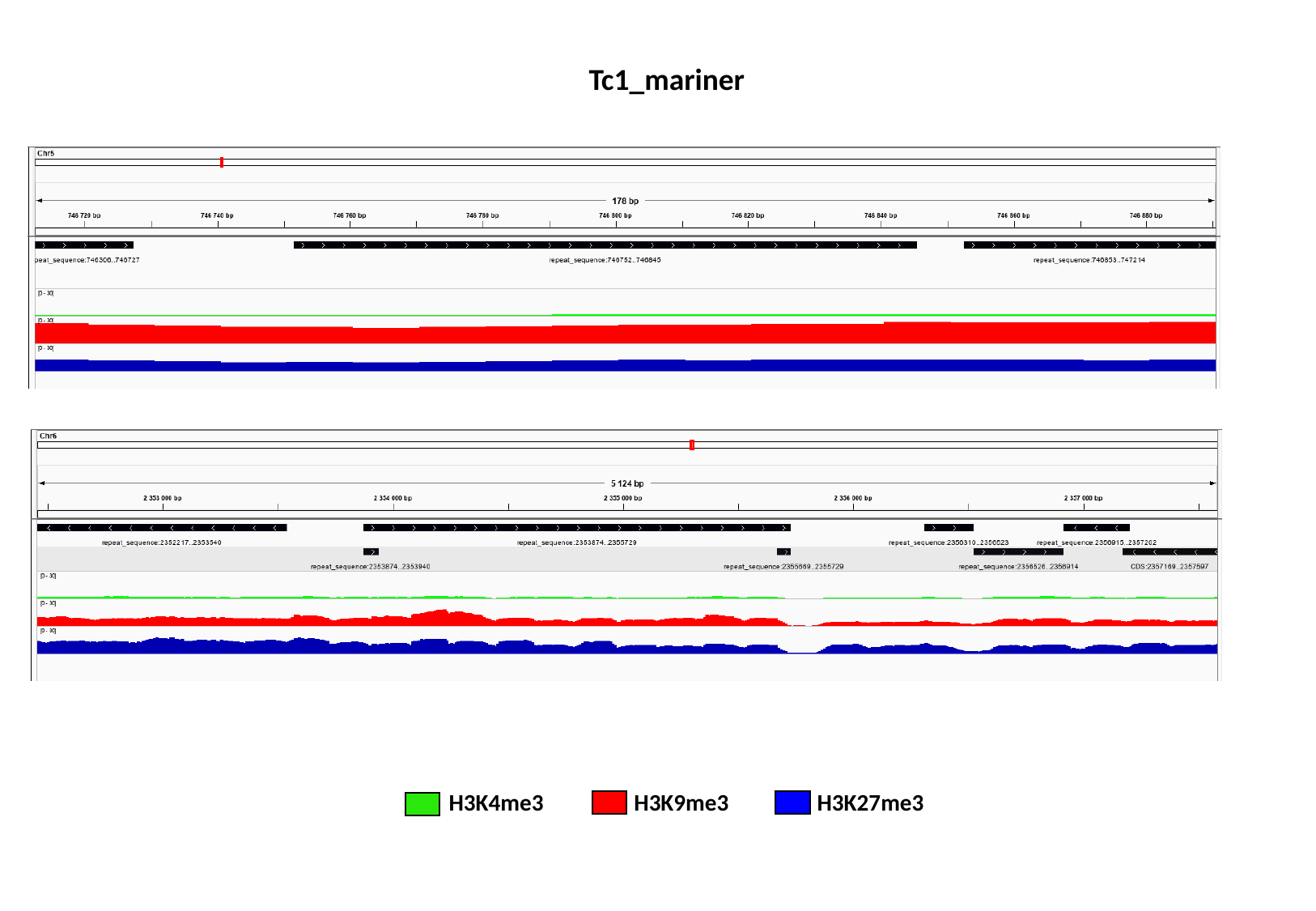

Tc1_mariner
H3K4me3
H3K9me3
H3K27me3

## Slide 4
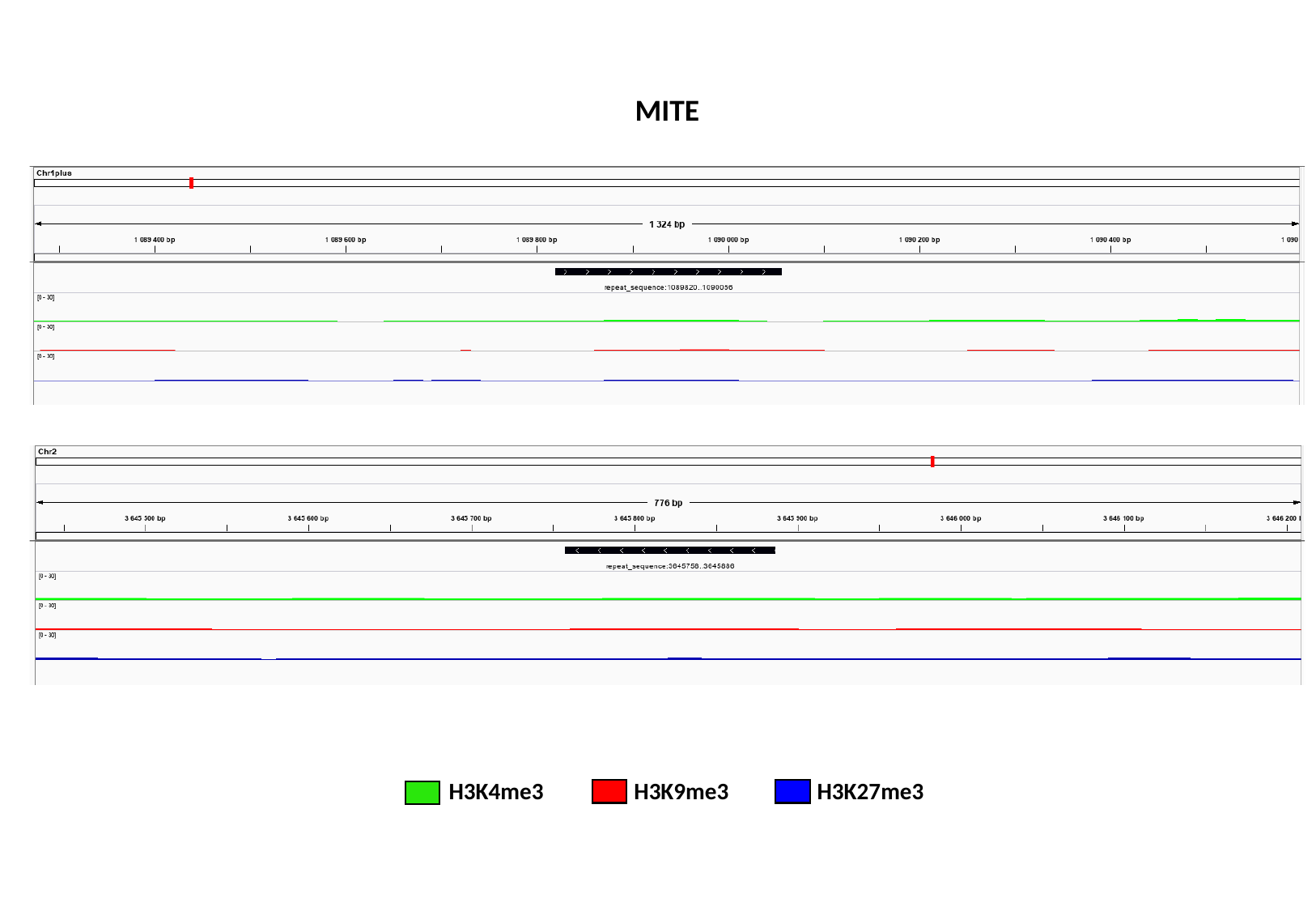

MITE
H3K4me3
H3K9me3
H3K27me3

## Slide 5
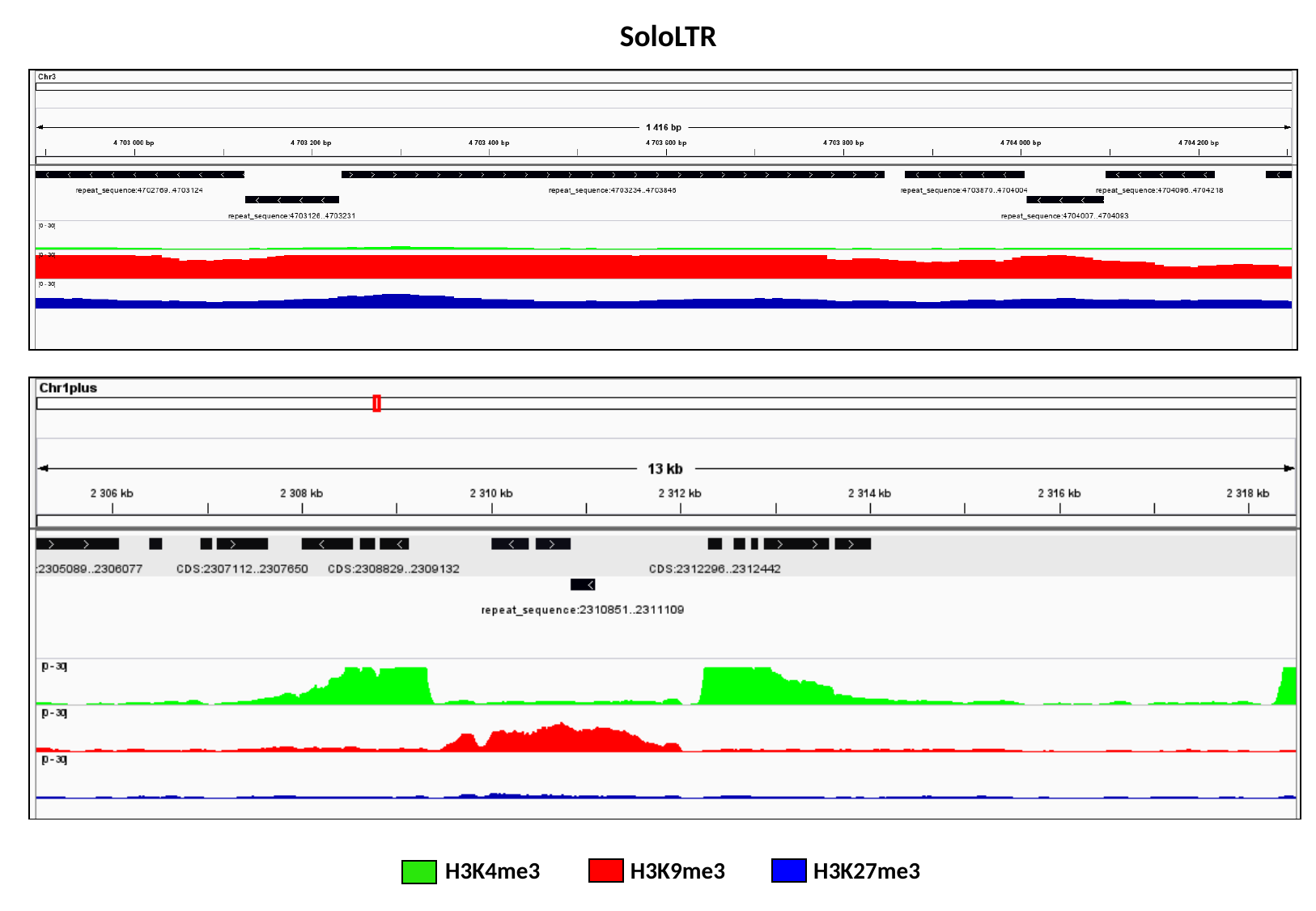

SoloLTR
H3K4me3
H3K9me3
H3K27me3

## Slide 6
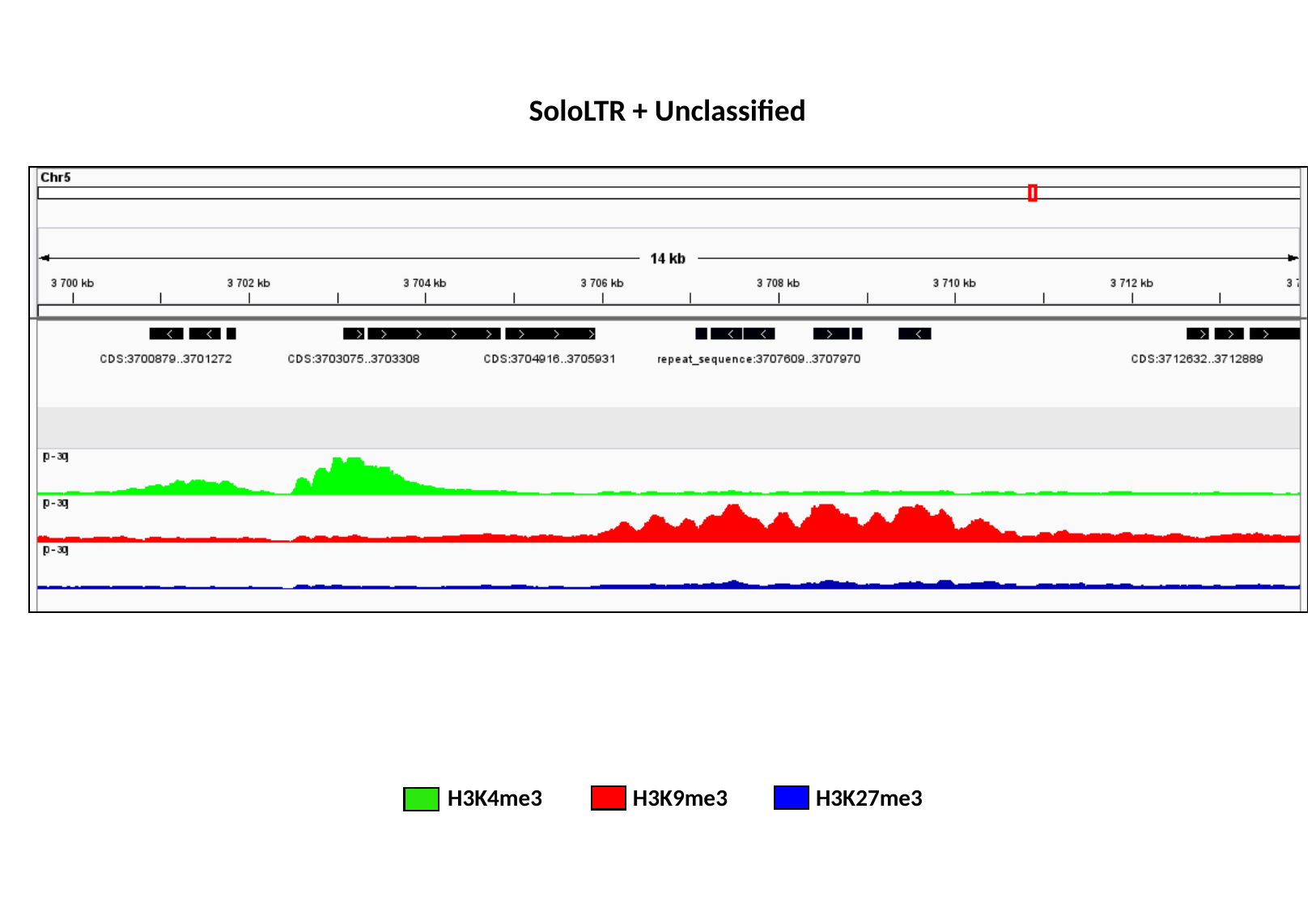

SoloLTR + Unclassified
H3K4me3
H3K9me3
H3K27me3

## Slide 7
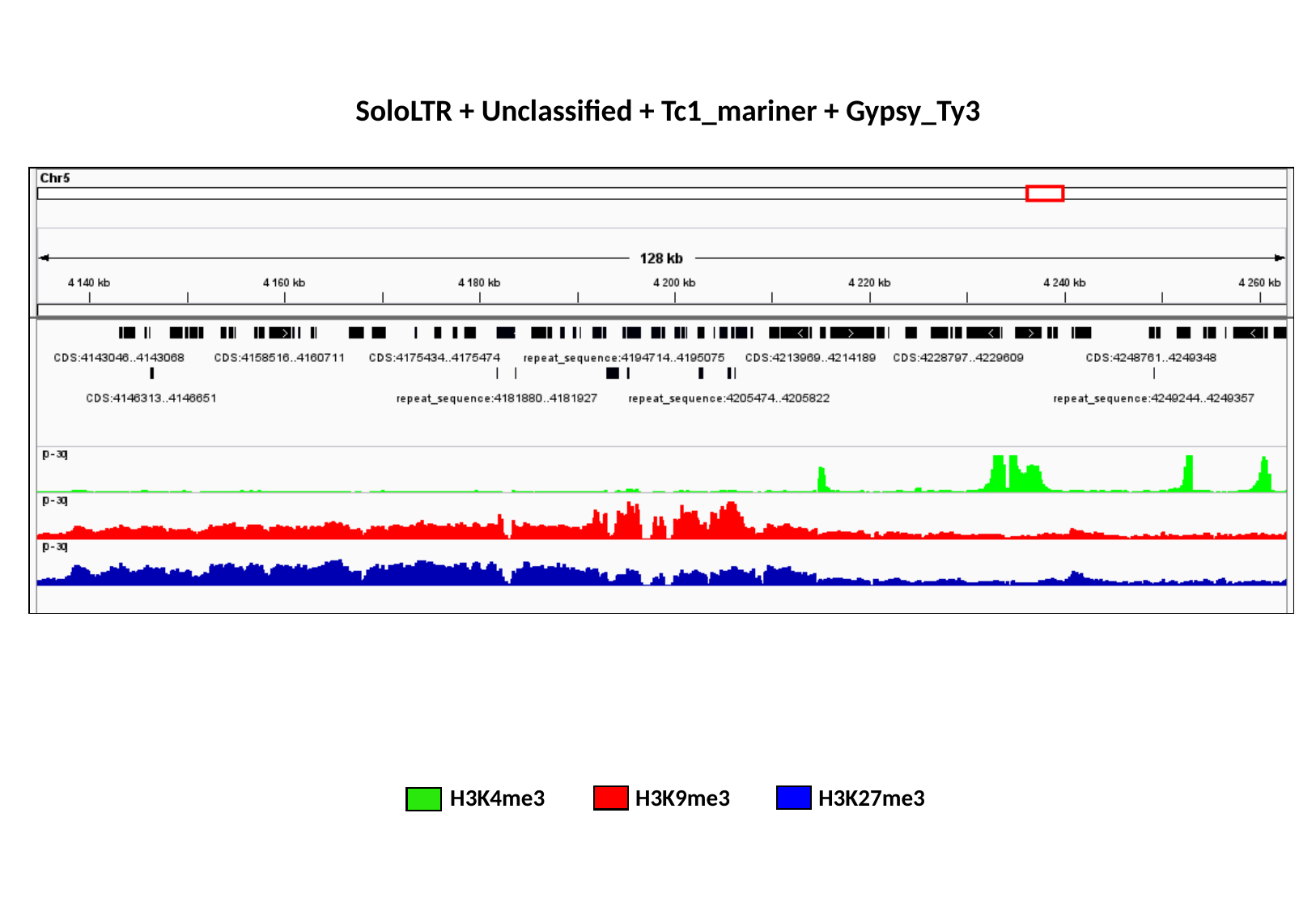

SoloLTR + Unclassified + Tc1_mariner + Gypsy_Ty3
H3K4me3
H3K9me3
H3K27me3
