# Supplementary material for: Loss of EZH2-like or SU(VAR)3–9-like proteins causes simultaneous perturbations in H3K27 and H3K9 tri-methylation and associated developmental defects in the fungus Podospora anserina
Source: Epigenetics Chromatin. 2021 May 7;14:22. doi: 10.1186/s13072-021-00395-7 (PMC8105982; doi:10.1186/s13072-021-00395-7)
Supplement: Supplementary file 8 — Additional file 8: Figure S8. Molecular characterization of knockout mutants by Southern blot hybridization. Schematic representations of the endogenous and disrupted loci are given (Left). Replacement by homologous recombination of the wild-type PaKmt1 allele by the disrupted ΔPaKmt1 allele results in the substitution of 2.4 kbp and 5.7 kbp EcoRV fragments by a unique 11 kbp PstI fragment as revealed by hybridization of the 5′ and 3′ digoxygenin-labeled probes (dashed rectangles PaKmt1 locus). Replacement by homologous recombination of the wild-type PaKmt6 allele by the disrupted ΔPaKmt6 allele results in the substitution of a unique 6.5 kbp KpnI fragment by two 1.8 and 2.4 kbp KpnI fragments as revealed by hybridization of the 5′ and 3′ digoxygenin-labeled probes (dashed rectangles PaKmt6 locus). A second verification has been made with the HindIII enzymes and the same probes shows the substitution of two 1.9 kbp and 4.6 kbp HindIII fragments by a 3.7 kbp HindIII fragment as revealed by hybridization of the 5′ and 3′ digoxygenin-labeled probes. [file 13072_2021_395_MOESM8_ESM.pptx]

## Slide 1
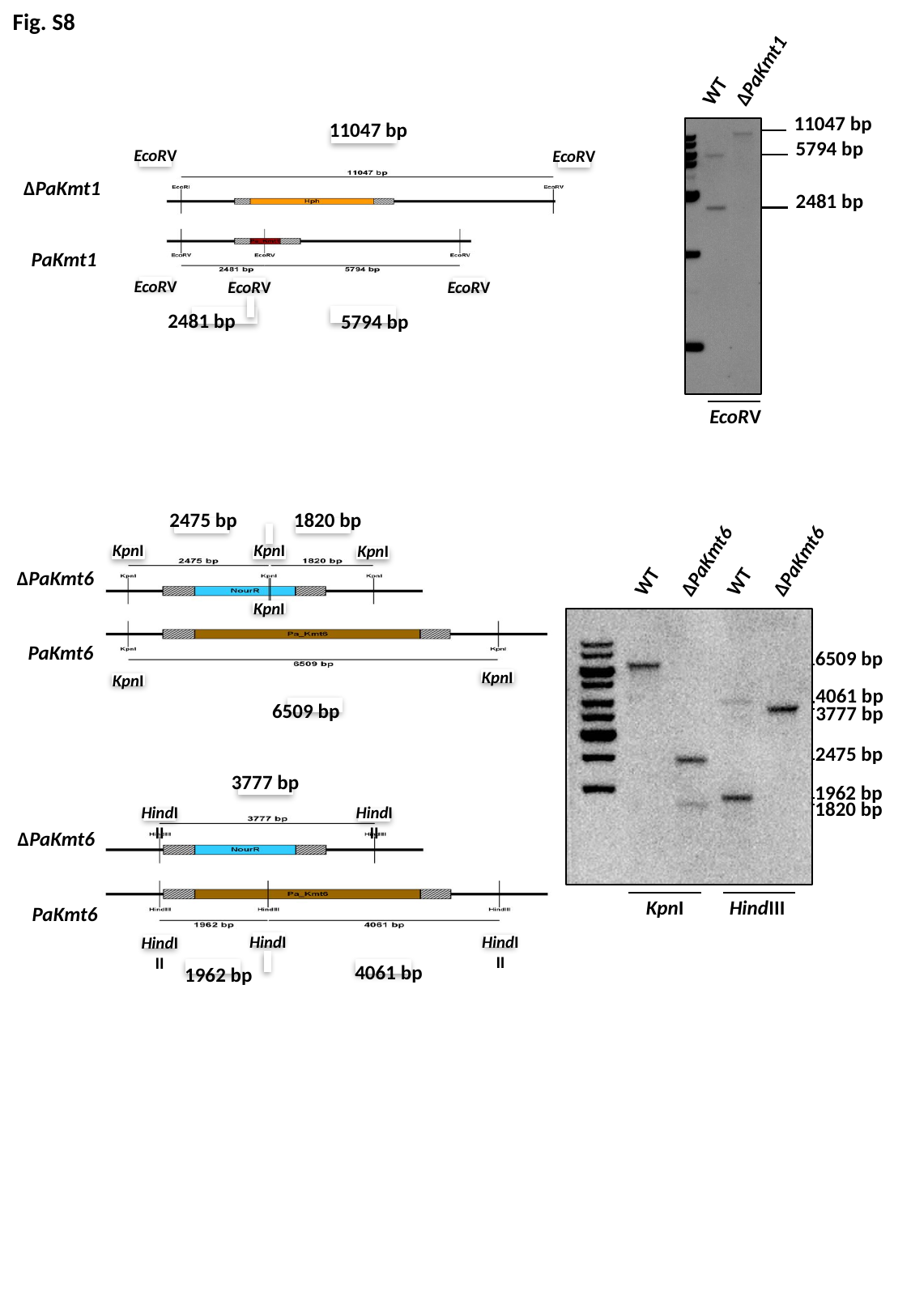

Fig. S8
WT
ΔPaKmt1
11047 bp
5794 bp
2481 bp
EcoRV
11047 bp
EcoRV
EcoRV
ΔPaKmt1
PaKmt1
EcoRV
EcoRV
EcoRV
2481 bp
5794 bp
2475 bp
1820 bp
KpnI
KpnI
KpnI
ΔPaKmt6
KpnI
PaKmt6
KpnI
KpnI
6509 bp
ΔPaKmt6
ΔPaKmt6
WT
WT
6509 bp
4061 bp
3777 bp
2475 bp
3777 bp
HindIII
HindIII
ΔPaKmt6
PaKmt6
HindIII
HindIII
HindIII
4061 bp
1962 bp
1962 bp
1820 bp
KpnI
HindIII
