# Supplementary material for: Loss of EZH2-like or SU(VAR)3–9-like proteins causes simultaneous perturbations in H3K27 and H3K9 tri-methylation and associated developmental defects in the fungus Podospora anserina
Source: Epigenetics Chromatin. 2021 May 7;14:22. doi: 10.1186/s13072-021-00395-7 (PMC8105982; doi:10.1186/s13072-021-00395-7)
Supplement: Supplementary file 9 — Additional file 9: Figure S9. Localization of histone marks on specific genomic regions in the ΔPaKmt1 and ΔPaKmt6 mutant strains. Top panel: Plots of normalized ChIP-seq signal. Bottom panel: Heatmaps divided in K-means built clusters representing the association versus non-association of the indicated histone modifications with the specific genomic regions. Coding sequences or CDS were aligned by their two ends (indicated by START and STOP) ± 1 kbp of surrounding sequence (N = 10,839; Additional file 20: Table S2); repeats were defined as TE bodies, duplications and the rDNA array ± 0.2 kbp surrounding regions (N = 1680; Additional file 21: Table S3). Histone modification levels in the heatmaps were calculated for non-overlapping 10 bp windows within the specific genomic regions and sorted by average value of each row. [file 13072_2021_395_MOESM9_ESM.pptx]

## Slide 1
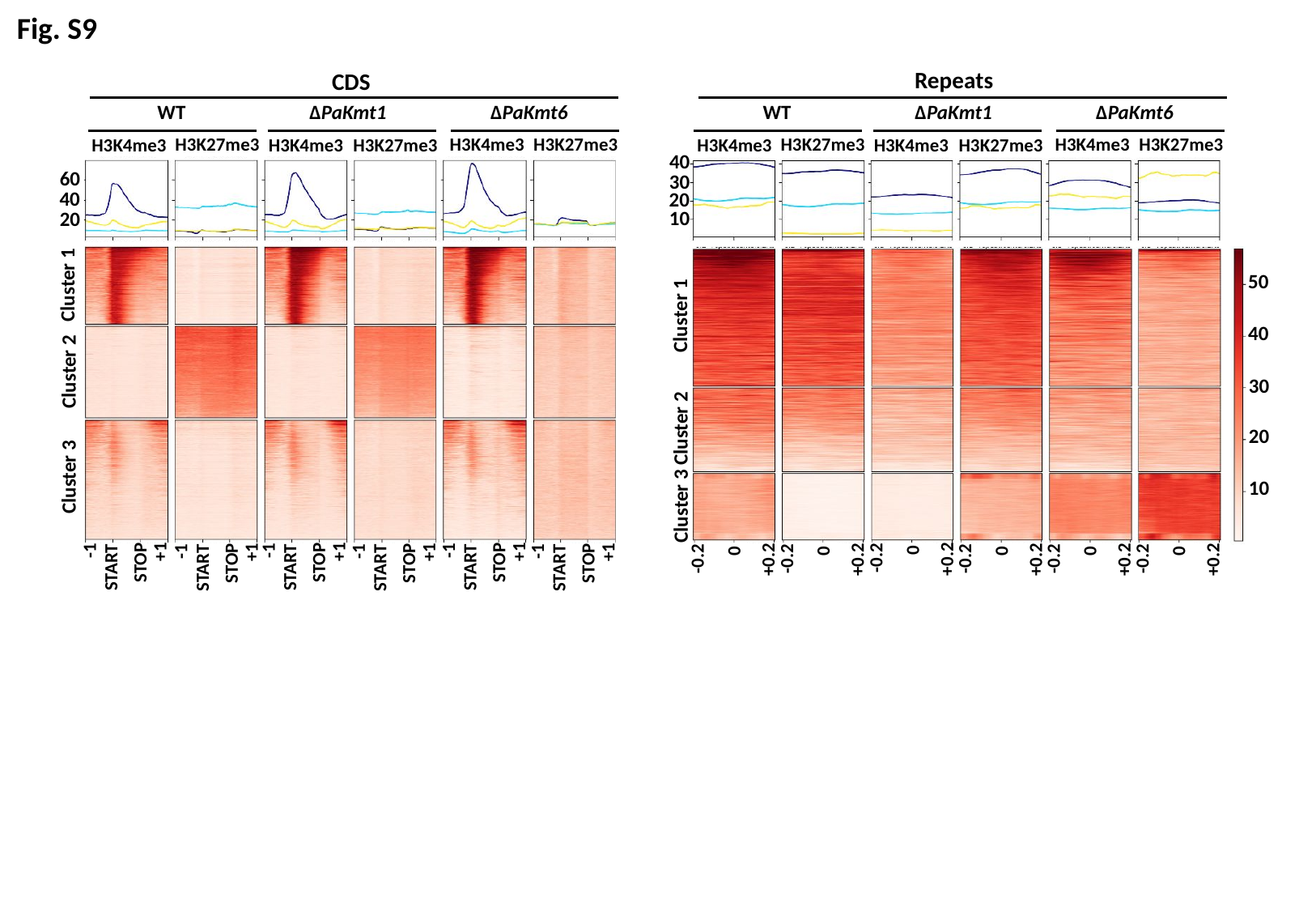

Fig. S9
Repeats
WT
ΔPaKmt1
ΔPaKmt6
H3K4me3
H3K27me3
H3K27me3
H3K4me3
H3K27me3
H3K4me3
40
30
20
10
50
Cluster 1
40
30
Cluster 2
20
10
Cluster 3
0
-0.2
+0.2
0
-0.2
+0.2
0
-0.2
+0.2
0
-0.2
+0.2
0
-0.2
+0.2
0
-0.2
+0.2
CDS
WT
ΔPaKmt1
ΔPaKmt6
H3K4me3
H3K27me3
H3K27me3
H3K4me3
H3K27me3
H3K4me3
60
40
20
Cluster 1
Cluster 2
Cluster 3
-1
+1
STOP
START
-1
+1
STOP
START
-1
+1
STOP
START
-1
+1
STOP
START
-1
+1
STOP
START
-1
+1
STOP
START
