# Supplementary material for: Loss of EZH2-like or SU(VAR)3–9-like proteins causes simultaneous perturbations in H3K27 and H3K9 tri-methylation and associated developmental defects in the fungus Podospora anserina
Source: Epigenetics Chromatin. 2021 May 7;14:22. doi: 10.1186/s13072-021-00395-7 (PMC8105982; doi:10.1186/s13072-021-00395-7)
Supplement: Supplementary file 11 — Additional file 11: Figure S11. Growth features of the ΔPaKmt1 strain. A Experimental procedure to test ability to resume growth of the ΔPaKmt1 strain. To set up this restart test, mycelium implants issued from germination thalli were inoculated onto fresh M2 medium and incubated at 27 °C for 8 days (step 1). Two independent plugs from each location (purple cross), 8-day stationary phase (plug#3); pink cross, 4-day stationary phase (plug#2); and green cross, growing phase (plug#1) were then transferred to fresh M2 solid medium and incubated for 3 days at 27 °C (step 2). The growing phase of each thallus originating from step 2 (green margins) were transplanted again to fresh M2 solid medium and incubated at 27 °C for 3 days (step 3). Growth restart from stationary phase was impaired for ΔPaKmt1 mutants, which resulted in smaller and thinner colonies than the wild-type ones (white arrows), whereas continuous growth (plug#1) was not altered. Complemented ΔPaKmt1-PaKmt1+ strains behaved as wild-type strains. As control experiments (step 3), we then transferred mycelia from growing margins (marked in green, step 2) of thalli deriving from Plug#1, Plug#2 and Plug#3. In this case, ΔPaKmt1 mutants did not show any delay to resume growth (orange arrows), confirming that this defect was not permanent but rather linked to the disability of the ΔPaKmt1 strains to resume growth properly. B Crippled growth test for ΔPaKmt1 strain. Crippled growth (CG) process can be shown using a ‘band test’. Strains were incubated on M2 medium for 7 days at 27 °C. Two 1-mm-wide slices of agar were then inoculated onto fresh M2 media with or without yeast extract (YE) in the following method for 3 days. Picture shows actively growing apical hyphae (right) and resting stationary phase hyphae (left). The surface side of the slice on plate has been orientated at the top. CG is a degenerative process caused by C element production in the stationary phase. When inoculated on yeast extract medium, it [file 13072_2021_395_MOESM11_ESM.pptx]

## Slide 1
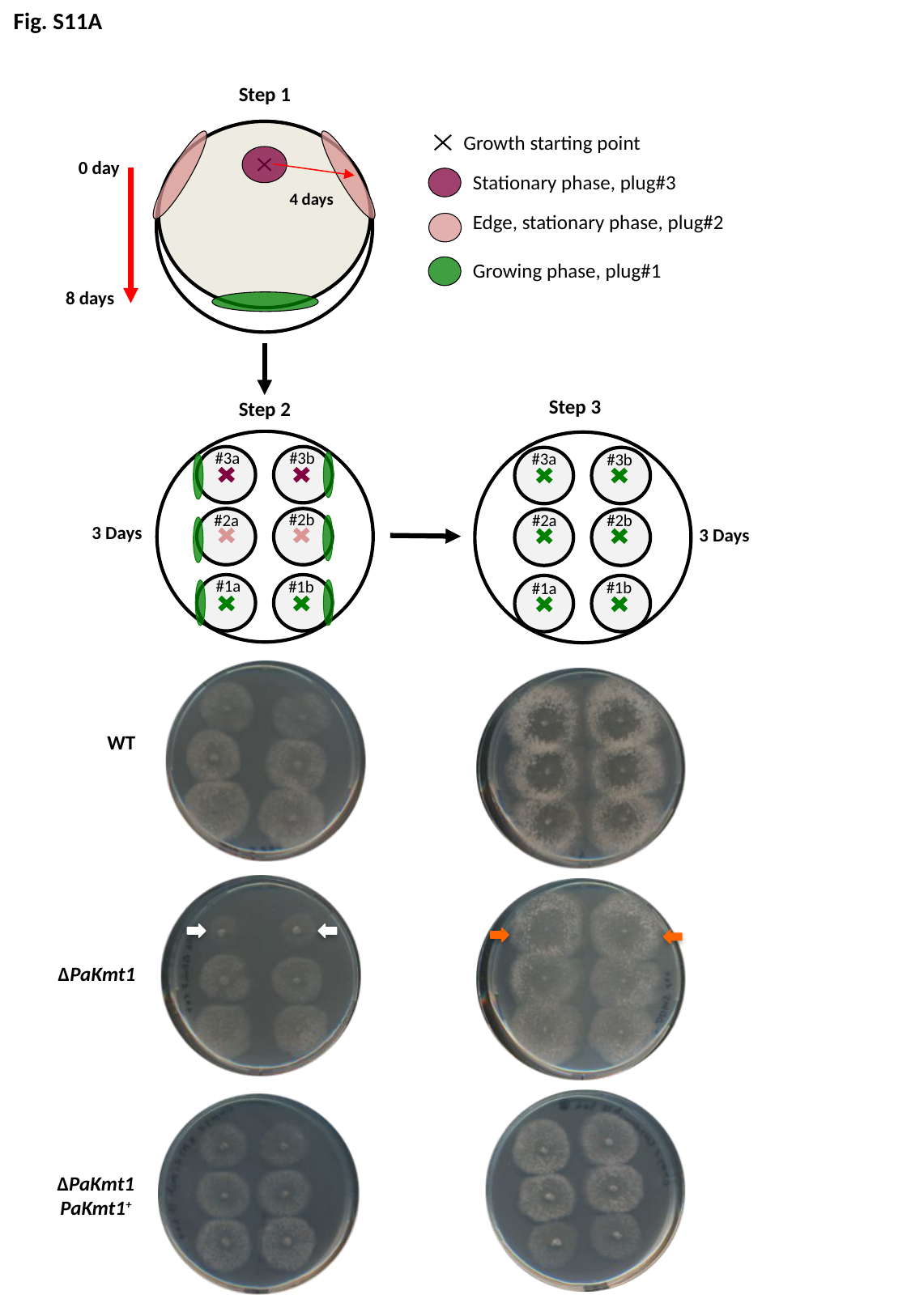

Fig. S11A
Step 1
Growth starting point
0 day
Stationary phase, plug#3
4 days
Edge, stationary phase, plug#2
Growing phase, plug#1
8 days
Step 3
Step 2
#3a
#3b
#3a
#3b
#2b
#2b
#2a
#2a
3 Days
3 Days
#1a
#1b
#1b
#1a
WT
ΔPaKmt1
ΔPaKmt1
PaKmt1+

## Slide 2
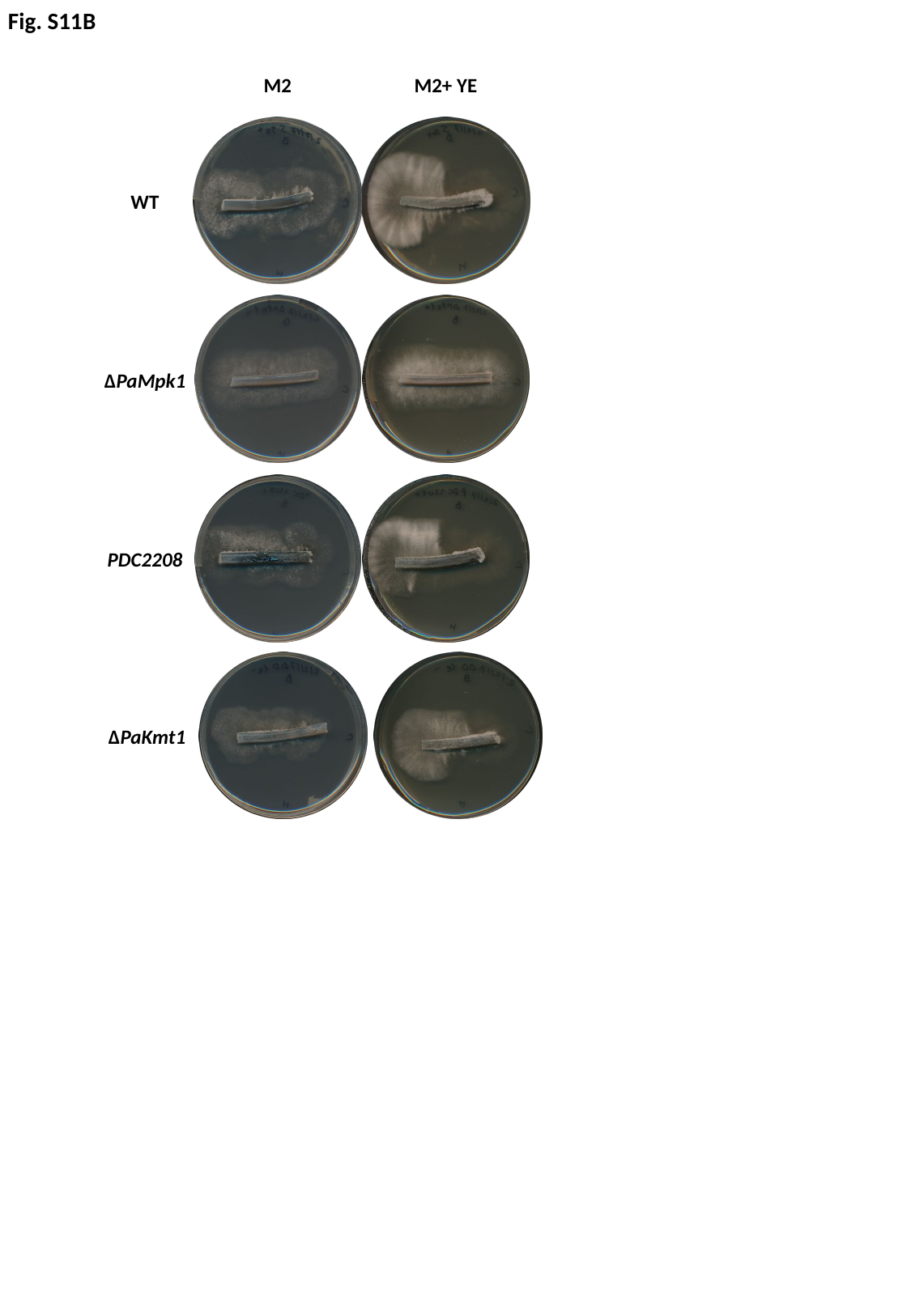

Fig. S11B
M2
M2+ YE
WT
ΔPaMpk1
PDC2208
ΔPaKmt1
