# Supplementary material for: Loss of EZH2-like or SU(VAR)3–9-like proteins causes simultaneous perturbations in H3K27 and H3K9 tri-methylation and associated developmental defects in the fungus Podospora anserina
Source: Epigenetics Chromatin. 2021 May 7;14:22. doi: 10.1186/s13072-021-00395-7 (PMC8105982; doi:10.1186/s13072-021-00395-7)
Supplement: Supplementary file 13 — Additional file 13: Figure S13. Role of PaKmt6 during sexual development. A Heterozygote oriented crosses between wild-type strains (WT) and ΔPaKmt6 mutants. Fertilization of wild-type ascogonia with ΔPaKmt6 male gametes results in normal fruiting body development displaying a single neck (black arrow), while fertilization of ΔPaKmt6 ascogonia with wild-type spermatia results in fewer crippled ΔPaKmt6-like fruiting bodies (two necks are indicated by two black arrows). These features indicate that PaKmt6 is a maternal gene. B Mosaic analyses using the Δmat mutant strain. Wild-type or ΔPaKmt6 mat+ and mat− strains were mixed with or without Δmat mycelium and inoculated onto fresh M2 medium. After a week of incubation on M2 medium, ΔPaKmt6 dikaryon displayed crippled and mis-orientated perithecia, resulting in scattered and reduced ascospore production. The tricaryon ΔPaKmt6 mat+ /ΔPaKmt6 mat−/Δmat showed nearly wild-type restoration of mycelium growth, as well as perithecia and ascospore production. These features confirm that PaKmt6 is a gene expressed in the maternal tissues of the perithecium and not in its zygotic tissues (centrum). [file 13072_2021_395_MOESM13_ESM.pptx]

## Slide 1
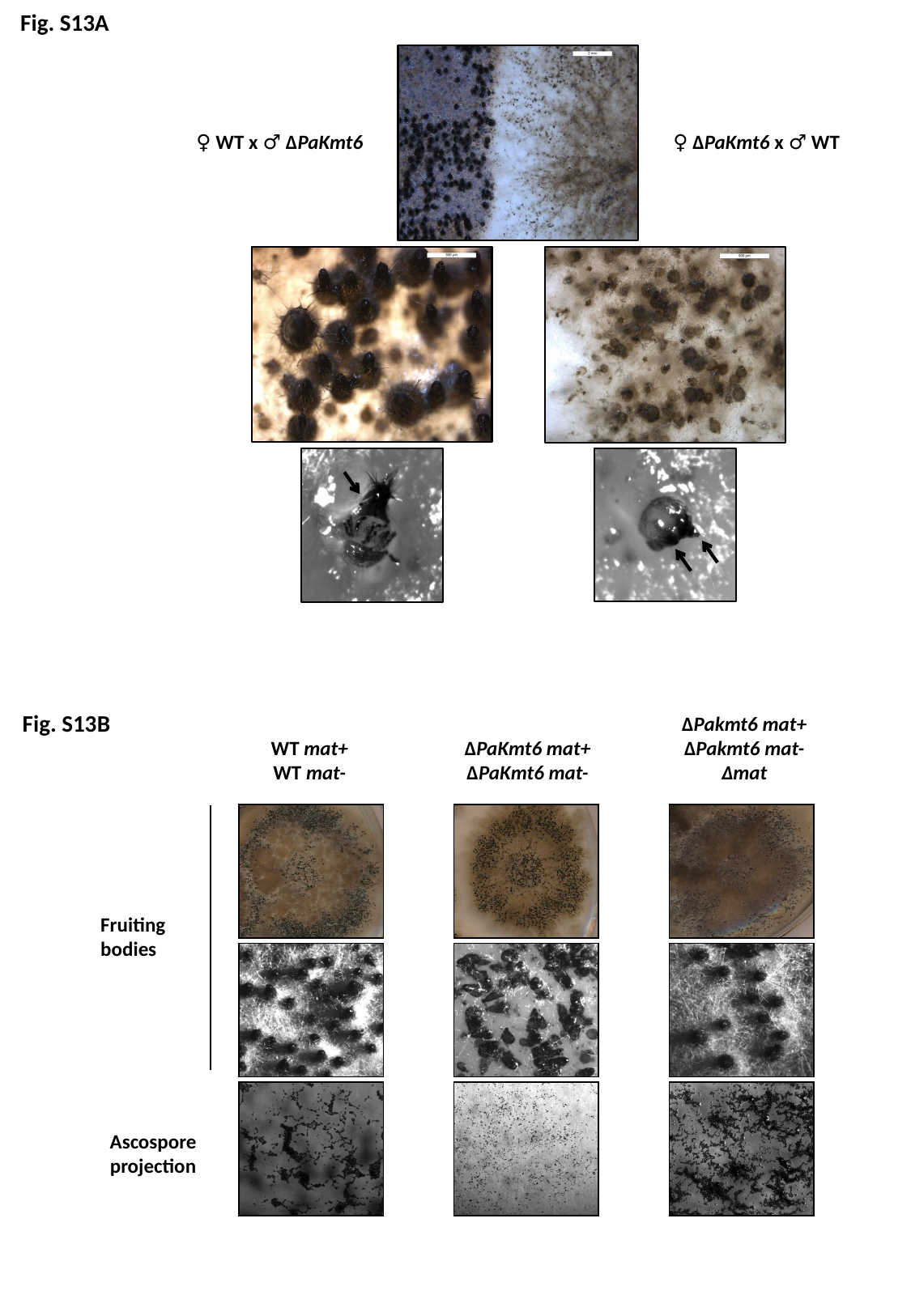

Fig. S13A
♀ WT x ♂ ΔPaKmt6
♀ ΔPaKmt6 x ♂ WT
Fig. S13B
ΔPakmt6 mat+ ΔPakmt6 mat- Δmat
WT mat+
WT mat-
ΔPaKmt6 mat+
ΔPaKmt6 mat-
Fruiting bodies
Ascospore projection
