# Supplementary material for: Loss of EZH2-like or SU(VAR)3–9-like proteins causes simultaneous perturbations in H3K27 and H3K9 tri-methylation and associated developmental defects in the fungus Podospora anserina
Source: Epigenetics Chromatin. 2021 May 7;14:22. doi: 10.1186/s13072-021-00395-7 (PMC8105982; doi:10.1186/s13072-021-00395-7)
Supplement: Supplementary file 15 — Additional file 15: Figure S15. A Tree showing evolution of some HP1 orthologs from fungi, plant and metazoans. The P. anserina heterochromatin protein-1 homolog is marked with a rectangle. Bootstraps are given. Filamentous fungi: Podospora anserina (Pa), Neurospora crassa (Nc), Fusarium graminearum (Fg), Trichoderma reesei (Tr), Epichloë festucae (Ef) Botrytis cinerea (Bc), Magnaporthe oryzae (Mo), Zymoseptoria tritici (Zt), Leptosphaeria maculans (Lm), Aspergillus nidulans (An), Aspergillus fumigatus (Af), Penicillium oxalicum (Po), Ascobolus immersus (Ai), Puccinia graminis (Pg), Pneumocystis jirovecii (Pj), Conidiobolus coronatus (Cc), Mucor circinelloides (Mc); yeasts: Schizosaccharomyces pombe (Sp) and Cryptococcus neoformans (Cn); the worm Caenorhabditis elegans (Ce), the fruit-fly Drosophila melanogaster (Dm), the mouse Mus musculus (Mm) and the model plant Arabidopsis thaliana (At). Accession numbers for proteins used in alignments are listed in Additional file 24: Table S6. B Molecular characterization of knockout ΔPaHP1 mutants by Southern blot hybridization. Replacement by homologous recombination of the wild-type PaHP1 allele by the disrupted ΔPaHP1 allele results in the substitution of a unique 3.4 kb EcoRI/EcoRV fragment by two 1.4 and 2.5 kb EcoRI/EcoRV fragments as revealed by hybridization of the 5′UTR and 3′UTR digoxygenin-labeled probes (dashed rectangles PaHP1 locus). C Vegetative growth of ΔPaHP1 single mutants and ΔPaKmt1ΔPaHP1 double mutants compared to wild-type strains. ΔPaKmt1 and ΔPaHP1 single mutants are impaired in aerial mycelium production. This defect was more pronounced for the single ΔPaHP1 mutants. ΔPaKmt1ΔPaHP1 double mutants show ΔPaKmt1 morphologic phenotype. The strains have been grown on M2 medium 3 days. [file 13072_2021_395_MOESM15_ESM.pptx]

## Slide 1
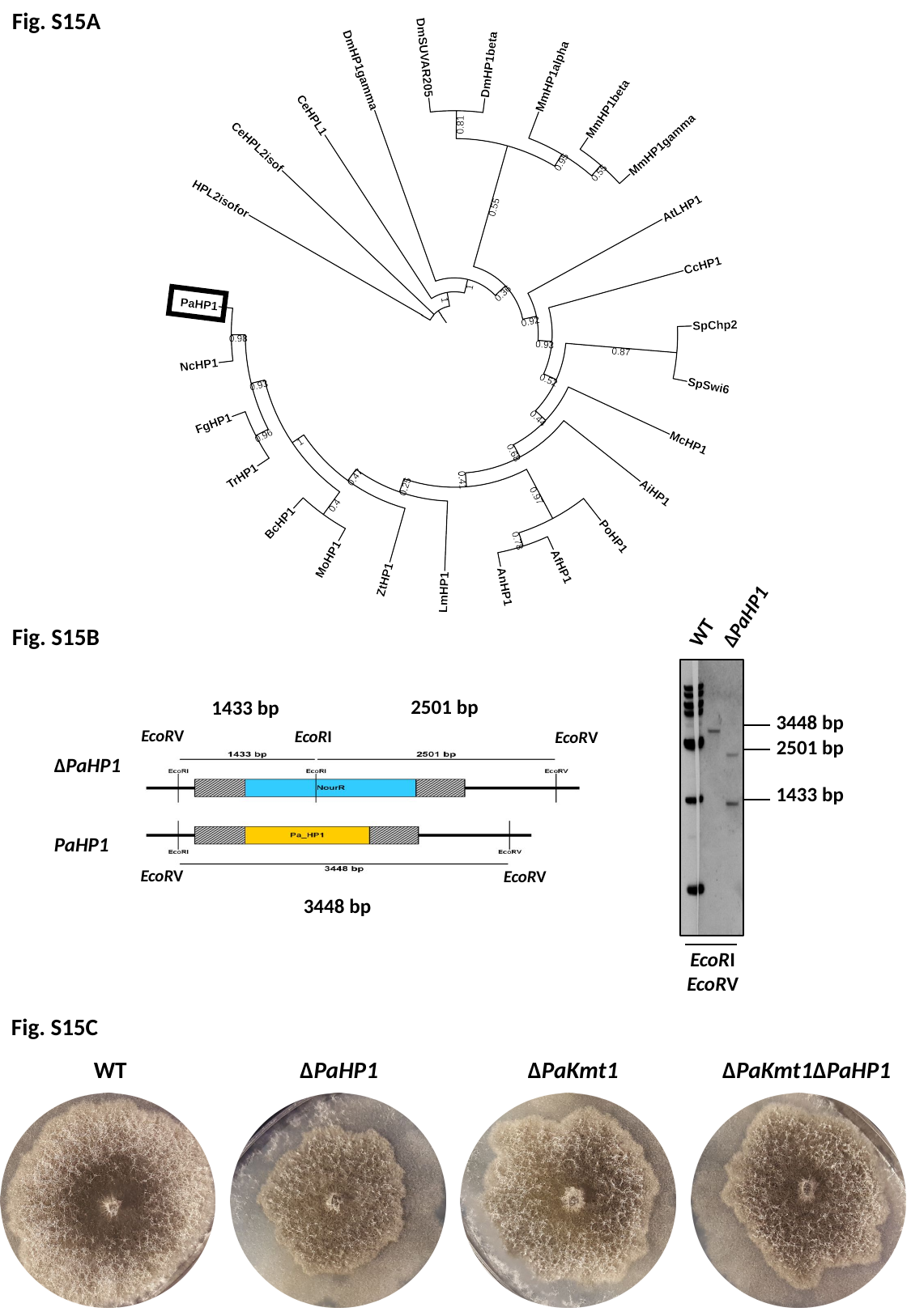

Fig. S15A
ΔPaHP1
WT
2501 bp
1433 bp
3448 bp
EcoRV
EcoRI
EcoRV
2501 bp
ΔPaHP1
1433 bp
PaHP1
EcoRV
EcoRV
3448 bp
EcoRI EcoRV
Fig. S15B
Fig. S15C
WT
ΔPaHP1
ΔPaKmt1
ΔPaKmt1ΔPaHP1
