# Supplementary material for: Loss of EZH2-like or SU(VAR)3–9-like proteins causes simultaneous perturbations in H3K27 and H3K9 tri-methylation and associated developmental defects in the fungus Podospora anserina
Source: Epigenetics Chromatin. 2021 May 7;14:22. doi: 10.1186/s13072-021-00395-7 (PMC8105982; doi:10.1186/s13072-021-00395-7)
Supplement: Supplementary file 17 — Additional file 17: Figure S17. A Relative expression of selected genes in the ΔPaHP1 strain. Caption as in Fig. 5a. The error bars represent the 95% confidence interval. No significant fold change was assayed, except for Pa_1_6263 and Pa_5_10, which both lost the H3K27me3 mark and were up-regulated and down-regulated, respectively. Pa_1_6263: expression ratio = 2.463, p-value = 0.004; Pa_4_1170: expression ratio = 1.911, p-value = 0.032; Pa_1_16300: expression ratio = 0.931, p-value = 0.693; Pa_6_7270: expression ratio = 1.527, p-value = 0.004; Pa_6_7370: expression ratio = 0.766, p-value = 0.009; Pa_5_10: expression ratio = 0.490, p-value = 0.006; Pa_1_1880: expression ratio = 1.313, p-value = 0.013; Pa_7_9210: expression ratio = 0.951, p-value = 0.547; TC1mlr represents quantification of the cDNAs from the members of the Tc1_mariner-like_rainette family: expression ratio = 1.264, p-value = 0.008. Tc1_mariner-like_pelobates and copia_Ty1_nephelobates transcripts could not be quantified as NRT-qPCR controls were too close to RT-qPCR (see Additional file 21: Table S3 for details of analysis). B Vegetative growth kinetics of ΔPaHP1 mutants and ΔPaKmt1 mutants compared to wild-type strains. See “Methods” section for details. C Experimental procedure to test growth resuming capabilities of ΔPaHP1 and double ΔPaHP1ΔPaKmt1 mutants. For description of experimental settings see Additional file 8: Fig. S8A. Growth restart from stationary phase (plug#2 and plug#3) was impaired for ΔPaHP1 and double ΔPaHP1ΔPaKmt1 mutants, which resulted in smaller and thinner colonies than the wild-type ones (white arrows), whereas continuous growth (plug#1) was not altered. Complemented ΔPaHP1-PaHP1+ strains behaved as wild-type strains. As control experiments (step 3), we transferred mycelia from growing margins (marked in green, step 2) of thalli derived from Plug#1, Plug#2 and Plug#3. In this case, neither ΔPaKmt1 mutants nor ΔPaHP1ΔPaKmt1 mutants showed any delay to resume growth (orange [file 13072_2021_395_MOESM17_ESM.pptx]

## Slide 1
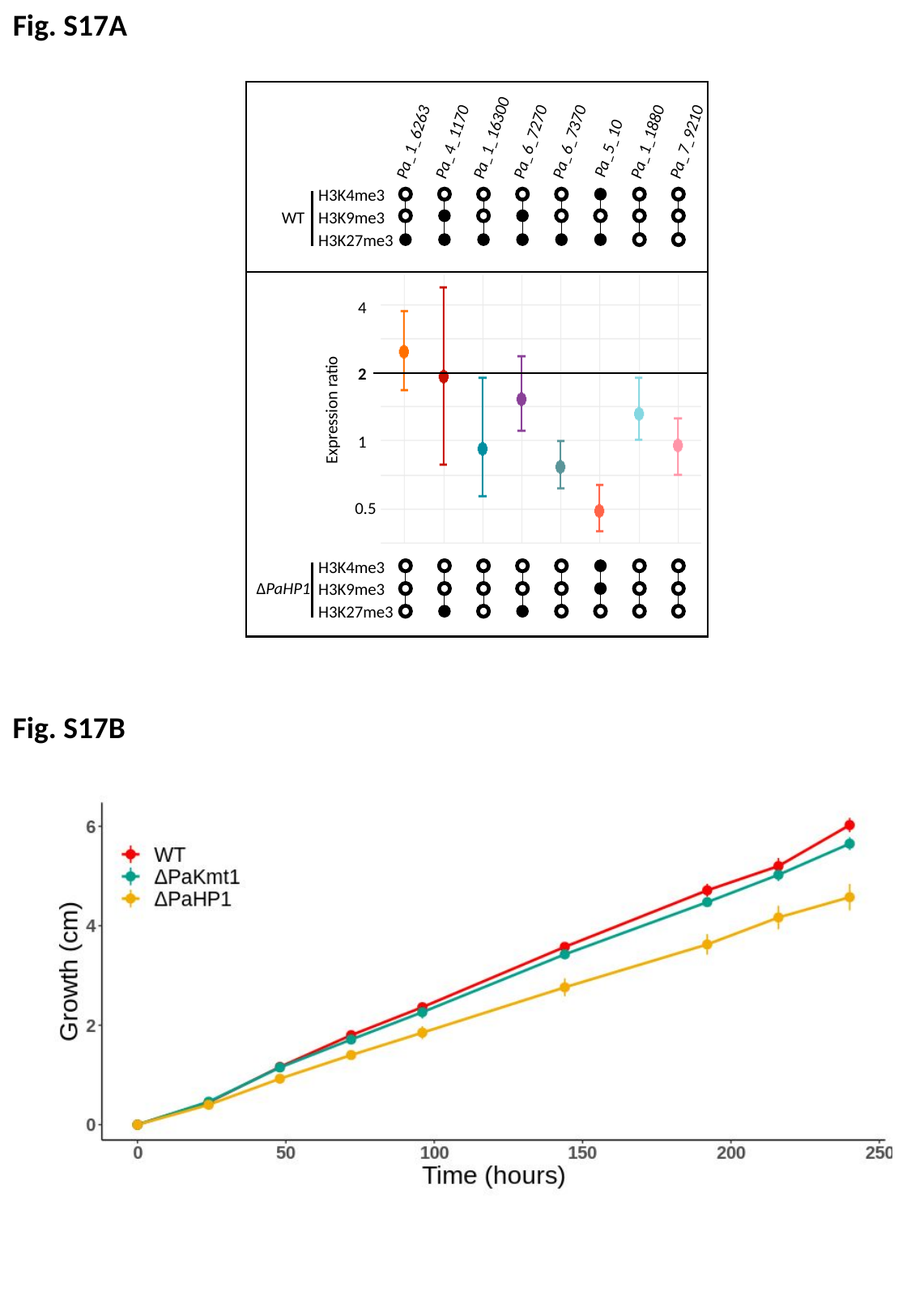

Fig. S17A
Pa_1_16300
Pa_1_6263
Pa_4_1170
Pa_6_7270
Pa_6_7370
Pa_1_1880
Pa_7_9210
Pa_5_10
H3K4me3
H3K9me3
WT
H3K27me3
4
2
Expression ratio
1
0.5
H3K4me3
∆PaHP1
H3K9me3
H3K27me3
Fig. S17B

## Slide 2
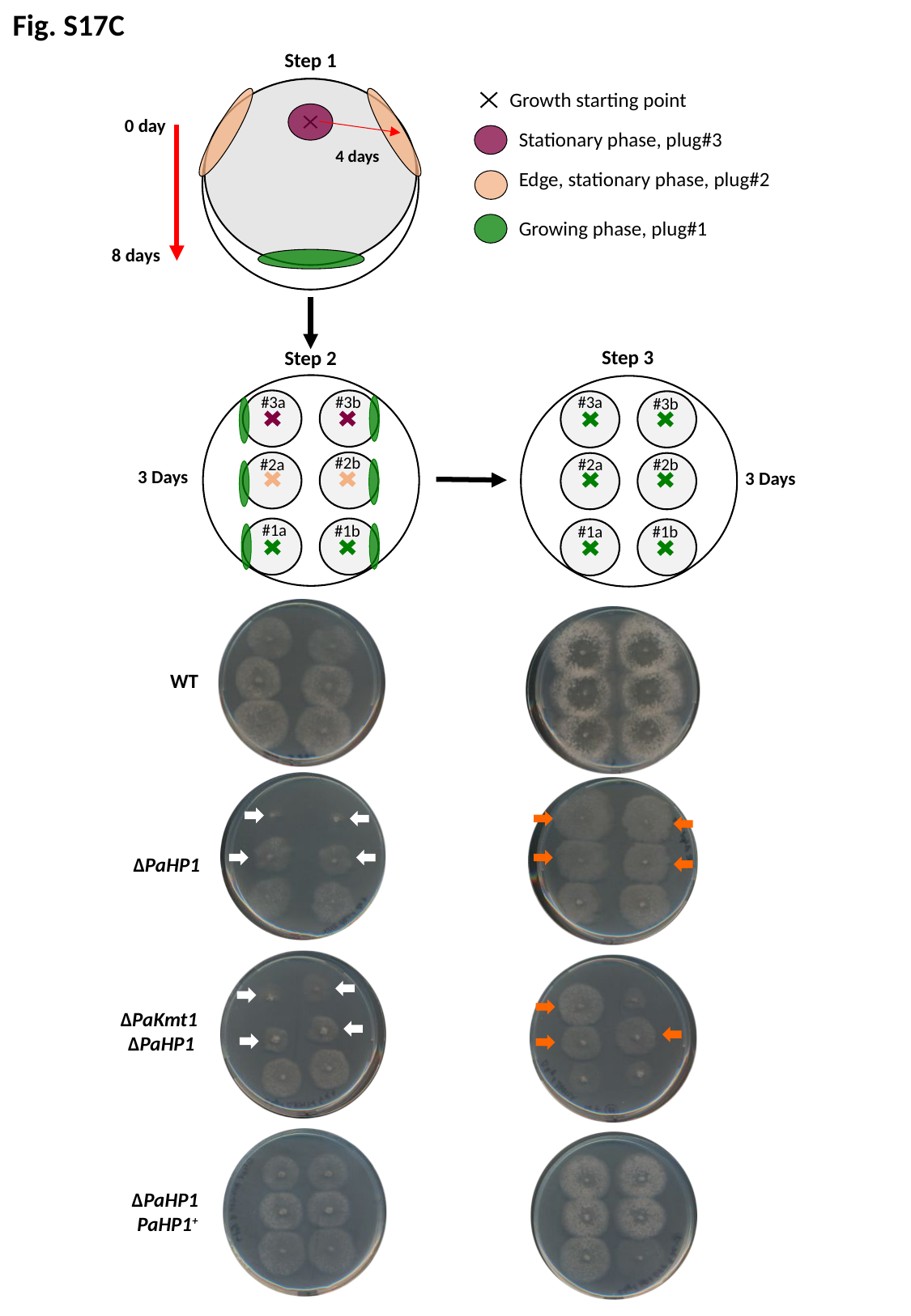

Fig. S17C
Step 1
Growth starting point
0 day
Stationary phase, plug#3
4 days
Edge, stationary phase, plug#2
Growing phase, plug#1
8 days
Step 3
Step 2
#3a
#3b
#3a
#3b
#2b
#2b
#2a
#2a
3 Days
3 Days
#1a
#1b
#1b
#1a
WT
ΔPaHP1
ΔPaKmt1
ΔPaHP1
ΔPaHP1
PaHP1+

## Slide 3
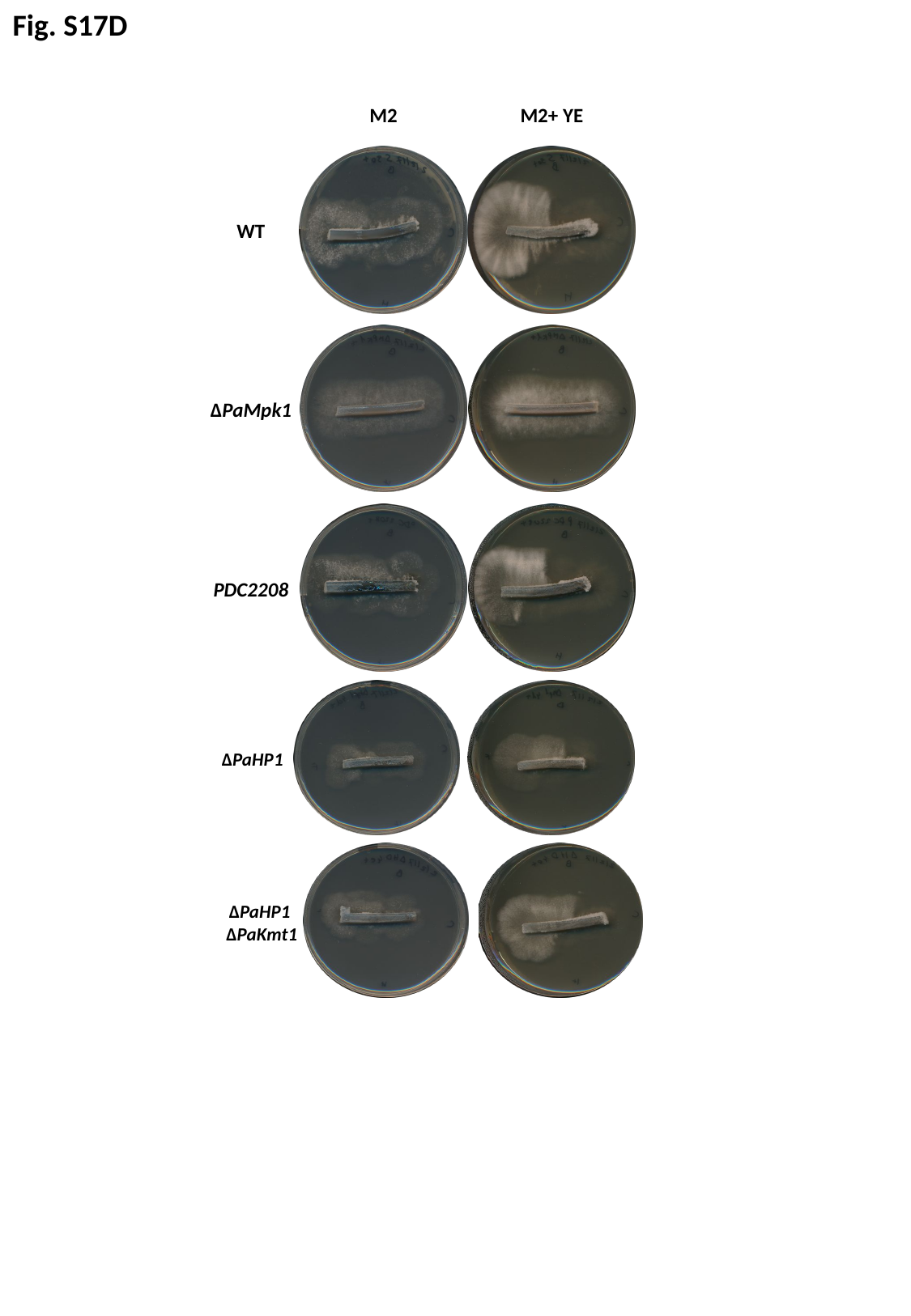

Fig. S17D
M2
M2+ YE
WT
ΔPaMpk1
PDC2208
ΔPaHP1
ΔPaHP1
ΔPaKmt1
