# Supplementary material for: Loss of EZH2-like or SU(VAR)3–9-like proteins causes simultaneous perturbations in H3K27 and H3K9 tri-methylation and associated developmental defects in the fungus Podospora anserina
Source: Epigenetics Chromatin. 2021 May 7;14:22. doi: 10.1186/s13072-021-00395-7 (PMC8105982; doi:10.1186/s13072-021-00395-7)
Supplement: Supplementary file 18 — Additional file 18: Figure S18. Heat map of Spearman’s correlation coefficient comparison. Clustering analysis of histone marks in wild-type background (WT) and in mutant backgrounds ΔPaKmt1, ΔPaKmt6 and ΔPaHP1. Mock = IP performed with GFP antibody in the absence of any GFP tag in the P. anserina genome (see “Methods”). Raw data are given in Additional file 26: Table S8. [file 13072_2021_395_MOESM18_ESM.pptx]

## Slide 1
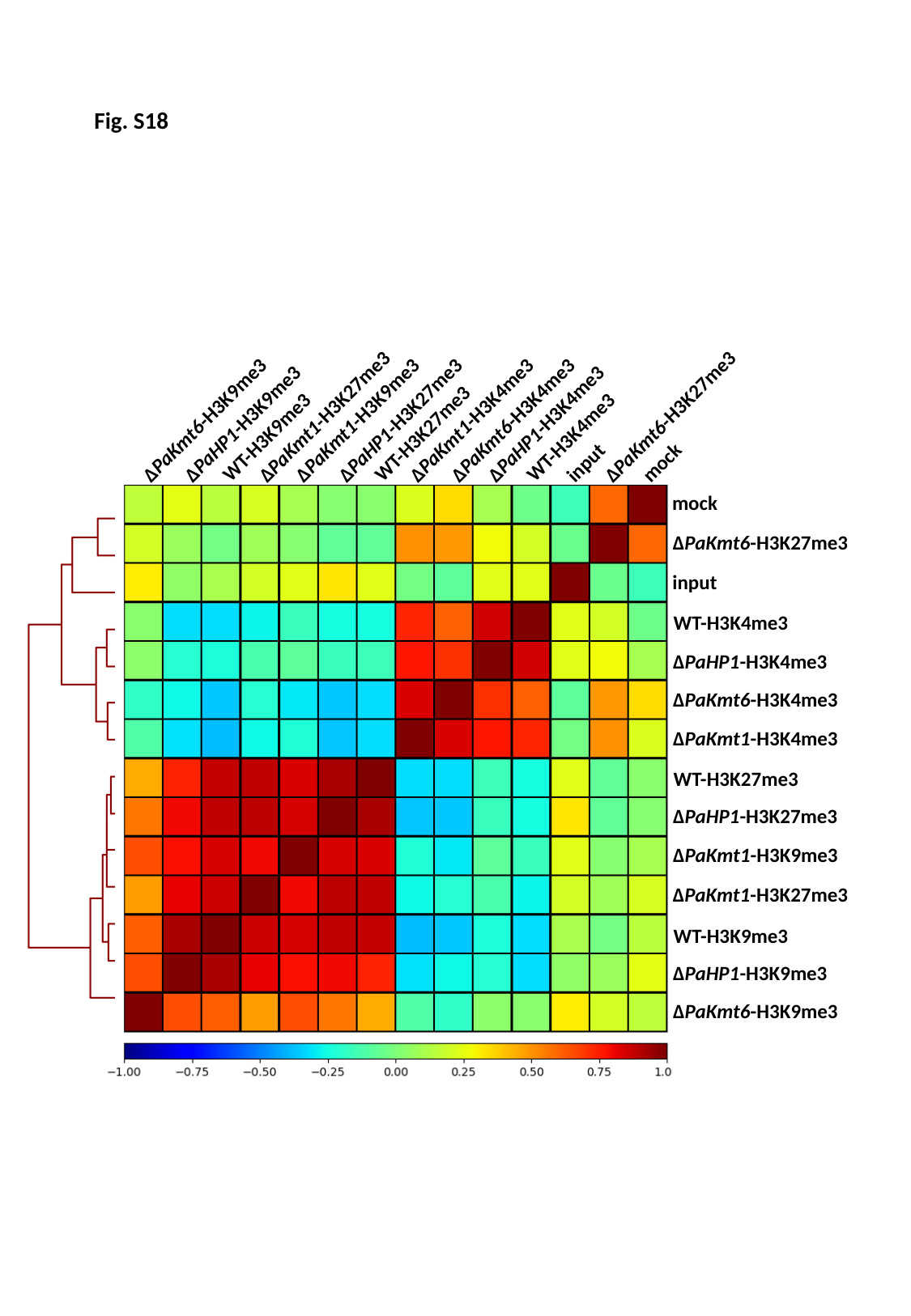

Fig. S18
ΔPaKmt1-H3K27me3
ΔPaKmt6-H3K27me3
ΔPaKmt6-H3K9me3
ΔPaKmt1-H3K9me3
ΔPaKmt1-H3K4me3
ΔPaKmt6-H3K4me3
ΔPaHP1-H3K27me3
ΔPaHP1-H3K9me3
ΔPaHP1-H3K4me3
WT-H3K27me3
WT-H3K9me3
WT-H3K4me3
input
mock
mock
ΔPaKmt6-H3K27me3
input
WT-H3K4me3
ΔPaHP1-H3K4me3
ΔPaKmt6-H3K4me3
ΔPaKmt1-H3K4me3
WT-H3K27me3
ΔPaHP1-H3K27me3
ΔPaKmt1-H3K9me3
ΔPaKmt1-H3K27me3
WT-H3K9me3
ΔPaHP1-H3K9me3
ΔPaKmt6-H3K9me3
