# Supplementary material for: Loss of EZH2-like or SU(VAR)3–9-like proteins causes simultaneous perturbations in H3K27 and H3K9 tri-methylation and associated developmental defects in the fungus Podospora anserina
Source: Epigenetics Chromatin. 2021 May 7;14:22. doi: 10.1186/s13072-021-00395-7 (PMC8105982; doi:10.1186/s13072-021-00395-7)
Supplement: Supplementary file 19 — Additional file 19: Table S1. Main features of significantly enriched peaks. Peak numbers, sizes, and genome coverage of H3K4me3, H3K9me3 and H3K27me3 modifications, in wild-type background (WT) and in mutant backgrounds ΔPaKmt1, ΔPaKmt6 and ΔPaHP1. [file 13072_2021_395_MOESM19_ESM.pptx]

## Slide 1
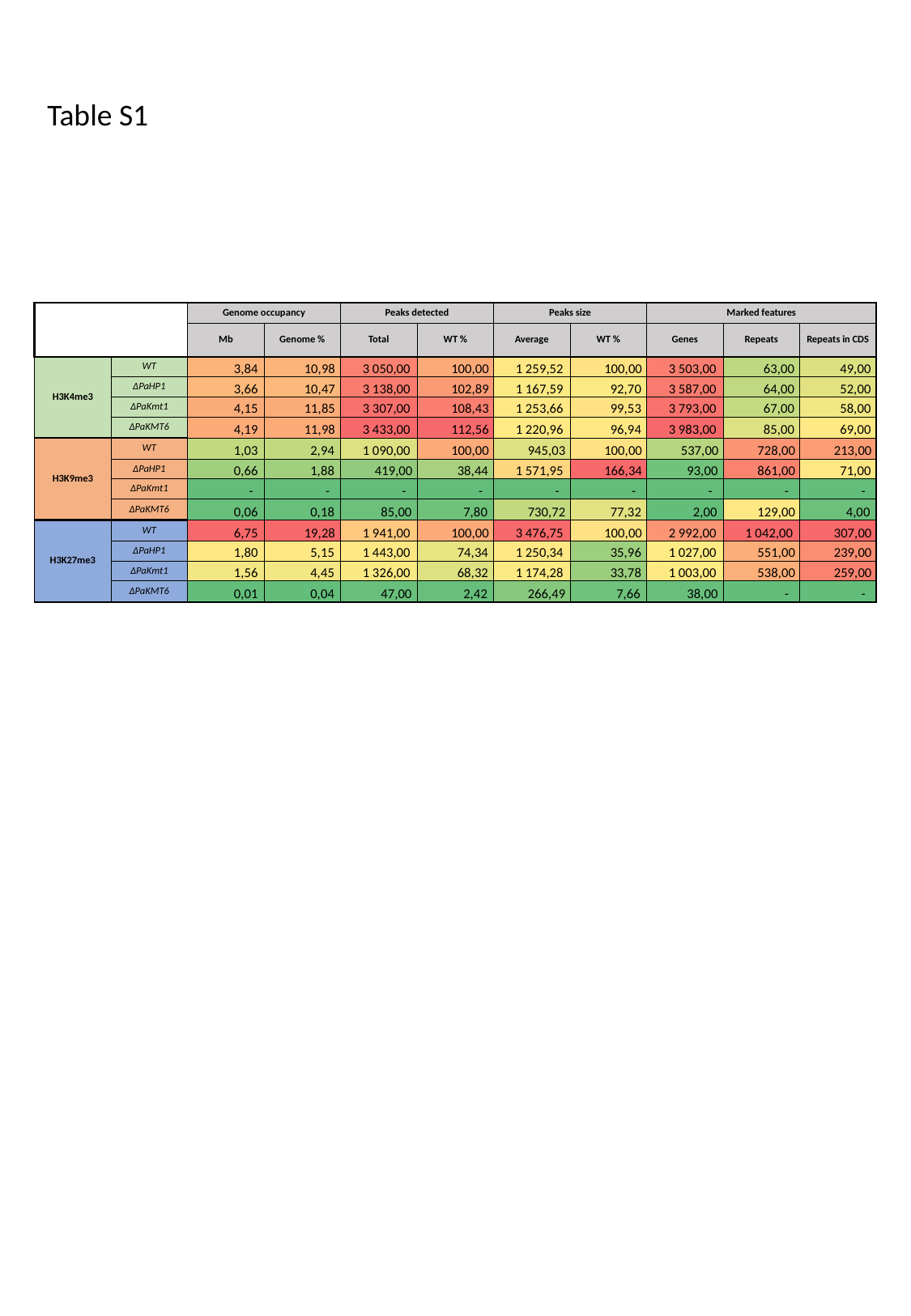

Table S1
| | | Genome occupancy | | Peaks detected | | Peaks size | | Marked features | | |
| --- | --- | --- | --- | --- | --- | --- | --- | --- | --- | --- |
| | | Mb | Genome % | Total | WT % | Average | WT % | Genes | Repeats | Repeats in CDS |
| H3K4me3 | WT | 3,84 | 10,98 | 3 050,00 | 100,00 | 1 259,52 | 100,00 | 3 503,00 | 63,00 | 49,00 |
| | ΔPaHP1 | 3,66 | 10,47 | 3 138,00 | 102,89 | 1 167,59 | 92,70 | 3 587,00 | 64,00 | 52,00 |
| | ΔPaKmt1 | 4,15 | 11,85 | 3 307,00 | 108,43 | 1 253,66 | 99,53 | 3 793,00 | 67,00 | 58,00 |
| | ΔPaKMT6 | 4,19 | 11,98 | 3 433,00 | 112,56 | 1 220,96 | 96,94 | 3 983,00 | 85,00 | 69,00 |
| H3K9me3 | WT | 1,03 | 2,94 | 1 090,00 | 100,00 | 945,03 | 100,00 | 537,00 | 728,00 | 213,00 |
| | ΔPaHP1 | 0,66 | 1,88 | 419,00 | 38,44 | 1 571,95 | 166,34 | 93,00 | 861,00 | 71,00 |
| | ΔPaKmt1 | - | - | - | - | - | - | - | - | - |
| | ΔPaKMT6 | 0,06 | 0,18 | 85,00 | 7,80 | 730,72 | 77,32 | 2,00 | 129,00 | 4,00 |
| H3K27me3 | WT | 6,75 | 19,28 | 1 941,00 | 100,00 | 3 476,75 | 100,00 | 2 992,00 | 1 042,00 | 307,00 |
| | ΔPaHP1 | 1,80 | 5,15 | 1 443,00 | 74,34 | 1 250,34 | 35,96 | 1 027,00 | 551,00 | 239,00 |
| | ΔPaKmt1 | 1,56 | 4,45 | 1 326,00 | 68,32 | 1 174,28 | 33,78 | 1 003,00 | 538,00 | 259,00 |
| | ΔPaKMT6 | 0,01 | 0,04 | 47,00 | 2,42 | 266,49 | 7,66 | 38,00 | - | - |
