# Supplementary material for: Loss of EZH2-like or SU(VAR)3–9-like proteins causes simultaneous perturbations in H3K27 and H3K9 tri-methylation and associated developmental defects in the fungus Podospora anserina
Source: Epigenetics Chromatin. 2021 May 7;14:22. doi: 10.1186/s13072-021-00395-7 (PMC8105982; doi:10.1186/s13072-021-00395-7)
Supplement: Supplementary file 23 — Additional file 23: Table S5. Primers used for PCR experiments. A Primers used for RT-PCR and allele construction experiments. B Primers used for RT-qPCR experiments. [file 13072_2021_395_MOESM23_ESM.docx]

**Table S5**

| **Experiments** | **Name** | **Sequence 5’>3’** |
| --- | --- | --- |
| PaKmt1 RT-PCR | 5s-DIM5 | CCCAGTCGAACAACCTCACCAAC |
|  | 3s-DIM5 | CCCTGTCAAGCCAGCAGTTTTG |
| Δ*PaHP1* allele | HP1L-2_MkF | CCAAACAAACCACCCCTCGTCGGTTCAGGGCAGGGTCGTTAAATAG |
|  | HP1L-3_MkR | GCCATCCCTCTGCTCGAAAACATCGAACTGGATCTCAACAGCGGTAAG |
|  | HP1L-1F | CACTCCTCGCCTCATCTTCC |
|  | Mk_HP1L-2R | CTATTTAACGACCCTGCCCTGAACCGACGAGGGGTGGTTTGTTTGG |
|  | Mk_HP1L-3F | CTTACCGCTGTTGAGATCCAGTTCGATGTTTTCGAGCAGAGGGATGGC |
|  | HP1L-4R | AGGTTGGCAAATGTGGGAGC |
| Δ*PaKmt1* allele | DIM5-1F | TTTGTCCTTGTTTTTGGGTATGC |
|  | Mk_DIM5L-2R | CTATTTAACGACCCTGCCCTGAACCGTTCGTTGACGATGGTGATGG |
|  | Mk_DIM5L-3F | CTTACCGCTGTTGAGATCCAGTTCGATGTGGATGAACAAGAGAAACAAACG |
|  | DIM5-4R | CTCACAACAACAAGACTCCAACG |
|  | DIM5L-2_MkF | CCATCACCATCGTCAACGAAGTTCAGGGCAGGGTCGTTAAATAG |
|  | DIM5L-3_MkR | CGTTTGTTTCTCTTGTTCATCCACATCGAACTGGATCTCAACAGCGGTAAG |
| Δ*PaKmt6* allele | KMT6-1F | ACACCGCGAGTATTGATAAACC |
|  | Mk_KMT6-2R | CTATTTAACGACCCTGCCCTGAACCGGTCAAATCCACCACAATTCTCC |
|  | Mk_KMT6-3F | CTTACCGCTGTTGAGATCCAGTTCGATGACGGAATTGCTTGACACTAAGG |
|  | KMT6-4R | GAGGTGATAGGTGACGATGAGG |
|  | KMT6-2_MkF | GGAGAATTGTGGTGGATTTGACCGGTTCAGGGCAGGGTCGTTAAATAG |
|  | KMT6-2_MkR | CCTTAGTGTCAAGCAATTCCGTCATCGAACTGGATCTCAACAGCGGTAAG |
| *PaHP1-GFP-HA* allele | FC3-BamH1 | AAGGATCCGGAGATGCTTGTCCGTCCCTCC |
|  | FC4-GFP | GCCCTTGCTCACCATGGAGTTGGCAGGGGGTGAGTCG |
|  | FC5-HP1 | CCCCCTGCCAACTCCATGGTGAGCAAGGGCGAGGAGC |
|  | FC6-HindIII | TTAAGCTTCTTGTACAGCTCGTCCATGCCGAGAGTGAT |
|  | FC21-BamH1 | AAGGATCCATGCCACCAGGTATGCTTCC |
| *PaKmt1-mCH-HA* allele | Dim5mChFBamH1 | AAGGATCCGAGCCTTGAGGATGTCTTTAGC |
|  | Dim5mChR | TTGATGATGGCCATCCATAAGAACCCCCTACACTTC |
|  | mChDim5F | AGGGGGTTCTTATGGATGGCCATCATCAAGGAGTTC |
|  | mChDim5RHindIII | TTAAGCTTCTTGTACAGCTCGTCCATGC |
|  | FC22-BamH1 | AAGGATCCATGGAGGAGGCAATGAAACAGC |
| *PaKmt6-GFP-HA* allele | FC73-GA1KMT6 | AAAGCTGGAGCTCCACCGCGGTGGCGAATGGCATGTATGGTCTG |
|  | FC74-GA1KMT6 | CCTTGCTCACCATCTCCTTCTCATCCCTATATCTC |
|  | FC75-GA1GFP | GGATGAGAAGGAGATGGTGAGCAAGGGCGAG |
|  | FC76-GA1GFP | GATATCGAATTCCTGCAGCCCGGGGCCCTTGTACAGCTCGTCCATGC |
| *PaKmt6-HA* allele | FC72-BamH1 | TTGGATCCCTCCTTCTCATCCCTATATCTCC |
|  | FC65-Not1 | TTGCGGCCGCGAATGGCATGTATGGTCTGTGC |

| **Type of gene^a^** | **Gene number** | **Gene name or function** | **Primer name** | **Primer sequence 5’>3’ ^b^** | **Amplicon size** |
| --- | --- | --- | --- | --- | --- |
| HKG | *Pa_2_11200* | *Actin* | FC77-Actin | CTCCCATCAACCCCAAGAGC | 86 bp |
|  |  |  | FC78-Actin | GATGGAGACGTAGAAGGCGG |  |
| HKG | *Pa_5_80* | *80* | FC125-580 | AGCTACTGTTGTCGTGCTGG | 54 bp |
|  |  |  | FC126-580 | TCGATGAAGAATACGGCGGC |  |
| HKG | *Pa_1_16650* | *AS1* | AS1f | CAACATGGCTGACGAATAC/AACGC | 115 bp |
|  |  |  | AS1r | GGAGGTCAGGTCAAGGAGA/GCATC |  |
| HKG | *Pa_3_6780* | *CIT1* | CIT1f | CTCCTCCAAGACCCAG/ACCCTC | 100 bp |
|  |  |  | CIT1r | GACCTTGGAGCCATGCTCC/TTTC |  |
| HKG | *Pa_3_5110* | *GPD* | GPDf | CATTGAGCCCAAGTACGCT/GAG | 113 bp |
|  |  |  | GPDr | GTCGCGCTCAGTGTAGAACTTGA |  |
| HKG | *Pa_2_6460* | *PAH1* | PAH1f | GATCTGGTTCCAGAACCG/ACGTG | 247 bp |
|  |  |  | PAH1r | GCAGTTGAGATGATGAATCA/AAAC |  |
| HKG | *Pa_7_6690* | *PDF2* | PDFf | GCAGACAGGTTCGAAAAG/ATTG | 294 bp |
|  |  |  | PDFr | CAGATGATCAATGGTT/TCTTGC |  |
| HKG | *Pa_4_8980* | *TBP* | TBPf | CACACCCACTCTTCA/GAACATT | 106 bp |
|  |  |  | TBPr | ACGCTTGGGGTTGTA/CTCAGC |  |
| HKG | *Pa_7_8490* | *TIP41* | TIPf | GTTTGCGGAGGTGAAGAAG/AA | 146 bp |
|  |  |  | TIPr | CCGTCTCACCCTCGAGAC |  |
| HKG | *Pa_4_7790* | *UBC* | UBCf | GGCCATCCCCATCCATCAAC | 107 bp |
|  |  |  | UBCr | GGTGATGGTCTTGCCAGTGA/GA |  |
| GoI | *Pa_6_7270* | *7270* | 7270f1 | TAGTCAACATGAAGGGAAGGA | 91 bp |
|  |  |  | 7270r1 | AGTGTAGTAGCGGCTGTCTGT |  |
| GoI | *Pa_6_7370* | *7370* | 7370f1 | CACCATCGCAGACCTCAA/CC | 206 bp |
|  |  |  | 7370r1 | GCTCGGTTTGGTGCCAGG |  |
| GoI | *Pa_1_6263* | *6263* | 6263f1 | TTGTCAACTTTCAGCCT/CC | 227 bp |
|  |  |  | 6263r1 | AAGGGACAAGTAAAACGG/CCT |  |
| GoI | *Pa_1_16300* | *16300* | 16300f1 | CACTTGGACACTCCTG/TCTA | 93 bp |
|  |  |  | 16300r1 | GGTGCCGCTGCCGTCGTA |  |
| GoI | *Pa_4_1170* | *1170* | 1170f4 | TCTGCGAAGGCGAGGAGT | 153 bp |
|  |  |  | 1170r4 | GGGTGATGACGGTGAAAGG |  |
| GoI | *Pa_1_1880* | *1880* | 1880f1 | AACTATTGCCAGCATTGAGG | 129 bp |
|  |  |  | 1880r1 | AGGTTCTTGCGGTAGGTGA |  |
| GoI | *Pa_5_10* | *10* | 10f1 | AGCAGGCCGAGGCATTAA | 91 bp |
|  |  |  | 10r1 | CCCGCAGCAACATAACCAT |  |
| GoI | *Pa_7_9210* | *9210* | 9210f1 | AATGACGCCGTTTACACCT | 173 bp |
|  |  |  | 9210r1 | GAATCCTCACCACCACTCG |  |
| GoI | *Pa_1_5210* | *5210* | 5210f1 | AGACATTGGCATCAAGAGTAGA | 190 bp |
|  |  |  | 5210r2 | GCAACAGAGGCAGTGGAG |  |
| GoI | Copia_Ty1  _  nephelobates |  | cTy1nf2 | CCATAACGCCCACAGCGGT | 137 bp |
|  |  |  | cTy1nr1 | GGGAGGGGAGCCTTTTATAGG |  |
| GoI | Tc1-mariner-like_  pelobates |  | Tc1mlpf2 | ATCCTCCTCGGTATATTTGGC | 152 bp |
|  |  |  | Tc1mlpr2 | CCACATTAAACCCTCCACAAC |  |
| GoI | Tc1_  mariner-like_rainette |  | Tc1mlrf1 | CCCATTCCTCGTCGATTCG | 165 bp |
|  |  |  | Tc1mlrr1 | CTCCTATTTCAGAAGCCAACC |  |

^a^: HKG: housekeeping gene, used as candidate for reference gene; GoI: gene of interest.

^b^: slash (/) indicates two consecutive exons.
